# Plasma microRNA Signature as Predictive Marker of Clinical Response to Therapy During Multiple Sclerosis

**DOI:** 10.1002/acn3.70093

**Published:** 2025-06-11

**Authors:** Fortunata Carbone, Alessandra Colamatteo, Teresa Micillo, Gianmarco Abbadessa, Silvia Garavelli, Clorinda Fusco, Claudia Russo, Francesco Perna, Federica Garziano, Claudia La Rocca, Maria Mottola, Giorgia Teresa Maniscalco, Simona Bonavita, Antonio Luca Spiezia, Alessia Castiello, Roberta Lanzillo, Vincenzo Brescia Morra, Claudio Procaccini, Paola de Candia, Giuseppe Matarese

**Affiliations:** ^1^ Istituto degli Endotipi in Oncologia, Metabolismo e Immunologia Consiglio Nazionale delle Ricerche (IEOMI‐CNR) Napoli Italy; ^2^ Unità di Neuroimmunologia IRCCS Fondazione Santa Lucia Roma Italy; ^3^ Dipartimento di Medicina Molecolare e Biotecnologie Mediche Università di Napoli “Federico II” Napoli Italy; ^4^ IRCCS Istituto Neurologico Mediterraneo Neuromed Pozzilli Italy; ^5^ UOSD Neurologia 2, Dipartimento di Scienze Mediche e Chirurgiche Avanzate Università Della Campania Luigi Vanvitelli Napoli Italy; ^6^ Department of Brain Sciences Imperial College London London UK; ^7^ Azienda Ospedaliera Universitaria “Federico II” Napoli Italy; ^8^ Dipartimento di Medicina Clinica e Chirurgia Università di Napoli “Federico II” Napoli Italy; ^9^ U.O.C. Biochimica Clinica A.O.R.N. Ospedali Dei Colli, Presidio Monaldi Napoli Italy; ^10^ Unità Operativa Complessa di Medicina Trasfusionale Azienda Ospedaliera Specialistica Dei Colli Napoli Italy; ^11^ Dipartimento di Neurologia, Centro Regionale Sclerosi Multipla Azienda Ospedaliera “A. Cardarelli” Napoli Italy; ^12^ Centro di Sclerosi Multipla, Dipartimento di Neuroscienze, Scienze Riproduttive ed Odontostomatologiche Università di Napoli “Federico II” Napoli Italy

**Keywords:** circulating microRNA, dimethyl fumarate (DMF), multiple sclerosis

## Abstract

**Objective:**

Despite the availability of effective therapies for Multiple Sclerosis (MS), the unpredictable nature of disease progression and the variability in individual treatment outcomes call for reliable biomarkers. This pilot study aims to investigate the potential of plasma circulating microRNAs (miRNAs) as predictive biomarkers for clinical responses to dimethyl fumarate (DMF), a widely used oral treatment for MS.

**Methods:**

Peripheral blood samples were collected from nineteen treatment‐naïve people with relapsing–remitting MS (pwRRMS) before and after 3, 6, 12, and 24 months of DMF administration, as well as from nineteen healthy individuals. MiRNAs were quantified by RT‐qPCR after plasma RNA extraction, and peripheral blood immune cells were analyzed by flow cytometry. Pathway enrichment and protein–protein interaction analyses were performed to identify the biological processes and molecular networks associated with mRNAs targeted by the specific DMF‐modulated miRNAs.

**Results:**

We identified a DMF‐modulated miRNA signature with significant changes occurring at early treatment stages. Notably, specific miRNAs were correlated with both clinical and immunological outcomes upon DMF treatment, including lymphocyte count reduction (let‐7b‐5p and miR‐223‐3p) and disease progression over 2 years (miR‐223‐3p, miR‐23a‐3p, miR‐23b‐3p, miR‐27a‐3p, and miR‐27b‐3p), suggesting their potential as predictive biomarkers for treatment response. Moreover, the validated mRNA targets of DMF‐modulated miRNAs were enriched for IL‐6 signaling and NRF2‐dependent antioxidant pathways, highlighting the potential molecular mechanisms underpinning DMF efficacy.

**Interpretation:**

This small exploratory study underscores the potential of plasma circulating miRNAs as candidate biomarkers for predicting therapeutic outcomes in MS and it calls for validation in larger studies, which may enhance our understanding of disease pathophysiology and offer a promising tool for personalized treatment strategies.

## 1 Introduction

Multiple sclerosis (MS) is a heterogeneous, multifactorial, immune‐mediated disease of the central nervous system (CNS) and represents the most common cause of non‐traumatic neurological disability in young adults [1]. Influenced by genetic and environmental factors, MS usually affects young adults, typically between the ages of 20 and 40, and is more common in women [2]. Its clinical course is highly heterogeneous; relapsing–remitting (RR)MS is the most common subtype, characterized by episodes of neurological dysfunction followed by periods of complete and/or partial recovery [3]. The neuropathological substrate of this course is mainly represented by demyelinating lesions, caused by the infiltration of immune cells, including T cells, B cells, and myeloid cells, into the CNS with consequent neuronal myelin injury [2]. Disease worsening can also occur in parallel with relapses since the early stages of the disease [4].

Several disease‐modifying therapies (DMTs) are available for the treatment of people with RRMS (pwRRMS), including oral, self‐injectable, and infusible drugs with different mechanisms of action [5]. Oral drugs are often preferred due to ease of administration and avoidance of injection‐related side effects [6]. Dimethyl fumarate (DMF, Tecfidera, Biogen Inc.), a fumaric acid ester with immunomodulating, anti‐inflammatory, and antioxidant properties, is widely used among pwRRMS [7]. Clinical trials (DEFINE and CONFIRM) demonstrated its efficacy in reducing relapse rates, slowing disability progression, and improving neuroradiological outcomes [8, 9, 10, 11]. DMF exerts its effects by activating nuclear factor erythroid 2–related factor 2 (NRF2), which maintains redox homeostasis and prevents oxidative stress and myelin damage through the modulation of antioxidant genes [12].

A recent study indicates that DMF‐induced NRF2 activation stimulates the cystine/glutamate antiporter solute carrier (SLC)7A11, enhancing GSH production, ROS removal, and regulatory T cell expansion, which promotes immune tolerance [13]. However, DMF also acts via NRF2‐independent pathways, including NF‐κB inhibition and activation of the prostaglandin EP2 receptor and cAMP pathways in immune cells, suppressing T cell proliferation and pro‐inflammatory cytokine production [14]. In addition, DMF reduces neutrophil infiltration into the CNS and clinical manifestations of experimental autoimmune encephalomyelitis (EAE), the mouse model of MS [14]. Despite a favorable safety profile, DMF may reduce absolute lymphocyte counts within 1 year, with severe lymphopenia occurring in ~30% of cases [10, 11, 15]. While studies suggest lymphopenia is not linked to treatment efficacy [16, 17], it is associated with opportunistic infections and progressive multifocal leukoencephalopathy (PML) due to John Cunningham Virus (JCV) reactivation [18].

MS shows significant heterogeneity in radiological, histopathological, clinical, and therapeutic responses. Molecular biomarkers could improve diagnosis, prognosis, and prediction of treatment efficacy and side effects. However, reliable biomarkers for treatment response before therapy initiation remain unavailable. MicroRNAs (miRNAs) are small, non‐coding, single‐stranded RNA molecules able to regulate a diversified range of biological processes, including innate and adaptive immune responses [19, 20]; furthermore, upon release into the extracellular space, miRNAs have been proposed as biomarkers for a plethora of complex, multifactorial diseases, including MS [21]. Specific circulating miRNA signatures can distinguish healthy individuals from people with MS and differentiate MS subtypes or MRI‐based phenotypes [22, 23, 24]. Nonetheless, the modulation and clinical significance of circulating miRNAs in response to immunomodulatory therapies, and in particular DMF, have not yet been fully evaluated. In line with published frameworks for biomarker development, this study represents an initial, hypothesis‐generating step focusing on the discovery and preliminary validation of candidate circulating miRNAs as predictive biomarkers of therapeutic response in MS. Hence, in this small exploratory study, we quantified the miRNA expression profile of a cohort of pwRRMS before and after DMF treatment to assess the potential of circulating miRNAs as candidate biomarkers for predicting disease course and response to DMF treatment.

## 2 Methods

### 2.1 Study Cohort and Sample Collection

This study was conducted in full compliance with ethical standards. The study was approved by the Institutional Review Board (IRB) of the Università degli Studi di Napoli “Federico II” (approval number 262/18) and a written informed consent was obtained from all participants in the study. We obtained peripheral blood from treatment‐naïve pwRRMS (*n* = 19) at the time of enrolment in the study (concomitantly at clinical assessment) and at 4 different time points after treatment with DMF (T1, 3 months, *n* = 11; T2, 6 months, *n* = 15; T3, 12 months, *n* = 10; T4, 24 months, *n* = 13). We also enrolled healthy individuals (*n* = 19) matched to pwRRMS for age, body mass index (BMI), and gender, and who had no history of inflammatory, endocrine, or autoimmune diseases. Demographic and clinical characteristics of healthy controls and pwRRMS are reported in the Table S1. The inclusion criteria for the pwRRMS were the following: subjects with a diagnosis of RRMS according to the revised McDonald criteria, treatment‐naïve, males and females aged 18 to 60 years, and steroid‐free over the past 3 months. The presence of important co‐morbidities (in particular additional autoimmune disorders) was a cause for patient exclusion from the study, and sex was not considered to be a biological variable. Specifically, for each patient, we collected blood samples for at least 3 different time points. All blood samples were collected at 9:00 a.m. in heparinized vacutainers (BD Biosciences) and processed within the following 4 h. Plasma samples were collected from peripheral blood samples and immediately stored at −80°C until use. All samples were collected, processed, and analyzed using the same standardized methods to further reduce bias.

### 2.2 Immunophenotypic Analysis by Flow Cytometry

Whole blood cells were analyzed with a clinical grade hemocytometer to determine absolute lymphocyte numbers in each sample. One hundred μL of blood were incubated for 30 min at room temperature with specific combinations of anti‐human antibodies. Red blood cells were lysed using IOTest 3 Solution (Beckman Coulter Life Sciences) for 15 min, and samples were subsequently washed and resuspended in 300 μL phosphate buffered saline (PBS). Flow cytometry was carried out by using AQUIOS Tetra‐1+ Panel Monoclonal Antibody Reagents consisting of CD45‐FITC (B3821F4A)/CD4‐RD1 (SFCI12T4D11)/CD8‐ECD (SFCI21Thy2D3)/CD3‐PC5 (UCHT1) and AQUIOS Tetra‐2+ Panel Monoclonal Antibody Reagents consisting of CD45‐FITC (B3821F4A)/(CD56 + CD16)‐RD1 (3G8 + N901)/CD19‐ECD (J3‐119)/CD3‐PC5 (UCHT1), PC7‐anti‐HLA‐DR (Immu‐357), and ECD‐anti‐CD45RO (UCHL1) (all from Beckman Coulter Life Sciences). Flow cytometry was carried out on cells gated on CD45+ − Side Scatter (SSC). Immunophenotypic analysis was performed with Cytomics FC500 Flow Cytometer (Beckman Coulter Life Sciences) with CXP software (Version 2.0) and Kaluza Analysis Flow Cytometry software (Version 2.1.1).

### 2.3 miRNA Quantification

RNA from 300 μL plasma samples was purified using the miRCURY RNA Isolation Kit—Biofluid (Qiagen, U.S.A.) according to the manufacturer's instructions. RNA samples were heparinase‐treated (0.5 Units/μl of isolated RNA) for 1 h at 25°C to recover miRNA detectability and then reverse‐transcribed with a miRCURY LNA Universal RT Kit and miRNA profiled by quantitative RT‐PCR, using SYBR Green master mix with locked nucleic acid (LNA)‐based miRNA primers (Qiagen, U.S.A.). Plates were run on the ABI QuantStudio 6 Flex qPCR machine (the same cycling conditions and parameters were maintained throughout the study). In a first sample set (6 from healthy controls and 18 samples from pwRRMS), 179 endogenous miRNAs (miRCURY LNA serum/plasma panel) were quantified and then 46 miRNAs expressed (Ct < 35) in all samples were selected for further analysis of the remaining samples included in the study. To ensure a balanced and unbiased approach, miRNA levels were quantified in both healthy controls and pwRRMS at multiple time points during treatment.

Relative quantitative values were calculated with the ΔCt method, using miRNA global mean as the biological normalization factor; miRNA quantitative data were graphed as log2 fold change to the relative healthy control cohort.

### 2.4 Cytokine and Soluble Factors Measurement

Plasma levels of Ghrelin, Leptin, Resistin, Adiponectin, Serpin E1, and Adipsin were evaluated using Human Magnetic Luminex Assays kits (R&D systems); plasma levels of GM‐CSF, IFNγ, IL1β, IL2, IL4, IL5, IL6, IL8 (CXCL8), IL10, IL12p70, IL17A (CTLA8), MCP1 (CCL2), TNFα, β amiloyd 1–42, BDNF, CNTF, FGF‐21, GDNF, GFAP, kallikrein‐6 (KLK6), MIF, NCAM‐1, Neurogranin (NRGN), NF‐H, NGFβ, S100B, Tau (pT181), Tau (total), TDP‐43, UCHL1, and YKL‐40 (CHI3L1) were evaluated using Human ProcartaPlex immunoassay kits (Thermo Fisher Scientific) according to the manufacturer's instructions. Fluorescence intensity was measured using the Luminex 200 system (Luminex), and data were analyzed with xPONENT Software Version 3.1 (Luminex). Cytokine levels were determined according to a standard curve generated for the specific target and expressed as picograms/mL (pg/mL).

### 2.5 Pathway Analyses

Predefined pathways were identified by importing the list of miRNA‐targeted mRNAs into the DAVID (Database for Annotation, Visualization, and Integrated Discovery) web interface to identify enriched biological processes, molecular functions, and cellular component terms from Gene Ontology (GO) annotations. The functional annotation clustering function in DAVID was used to group related GO terms into clusters, highlighting key functional themes. Additionally, pathway analysis was conducted using the Reactome and KEGG databases to identify biological pathways and networks relevant to the target mRNAs.

Protein–protein interaction (PPI) analysis was conducted using the STRING (Search Tool for the Retrieval of Interacting Genes/Proteins) database, which provided interaction data for the miRNA‐targeted mRNA. The resulting PPI network was imported into Cytoscape, where a connectivity analysis was performed to identify key nodes (hubs) within the network. The major hubs were determined based on their betweenness centrality score. This analysis provided insights into the structural properties of the network and the potential functional significance of the key proteins involved.

### 2.6 Statistical Analysis

Distribution of continuous variables was tested for normality by the Kolmogorov–Smirnov test. Comparisons between groups were performed by either unpaired Student's *t*‐test or non‐parametric Mann–Whitney test (in case of two‐groups) and by either ANOVA or Kruskal‐Wallis (in case of three or more groups) respectively for normally and not‐normally distributed variables. Longitudinal changes were analyzed by paired comparisons. Whisker plots were used to represent the distribution of miRNAs and immunometabolic factors (as either relative or absolute quantities as appropriate). Correlation between miRNAs and either immunometabolic factors or Expanded Disability Status Scale (EDSS) or lymphocyte counts was graphically represented by scatter plots and heatmaps. Any instances of missing samples at specific follow‐up time points were addressed by considering the data from the immediately subsequent time point for analysis, thereby minimizing the impact of loss to follow‐up on the overall study continuity. Graphical representations were drawn using GraphPad Prism software 7, with data presented as the mean ± SEM, unless stated otherwise.

## 3 Results

### 3.1 DMF Treatment Significantly Modulates the Plasma Circulating miRNome

**FIGURE 1:**
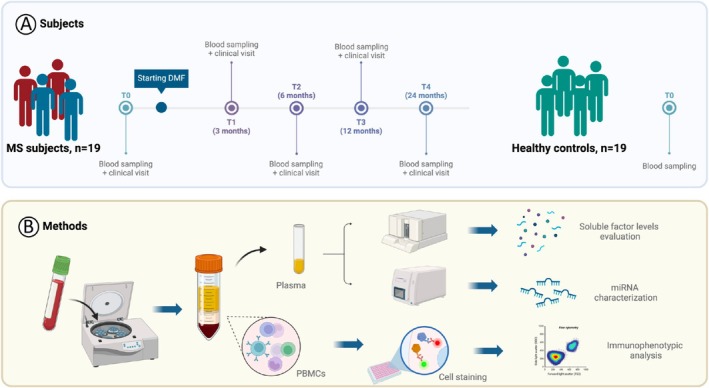
Schematic outline summarizing the study flow. (A) Schematic experimental design summarizing healthy controls and treatment‐naïve pwRRMS enrollment and (B) schematic overview of quantitative analyses of the blood samples performed in parallel is reported. PBMCs, peripheral blood mononuclear cells.

**FIGURE 2:**
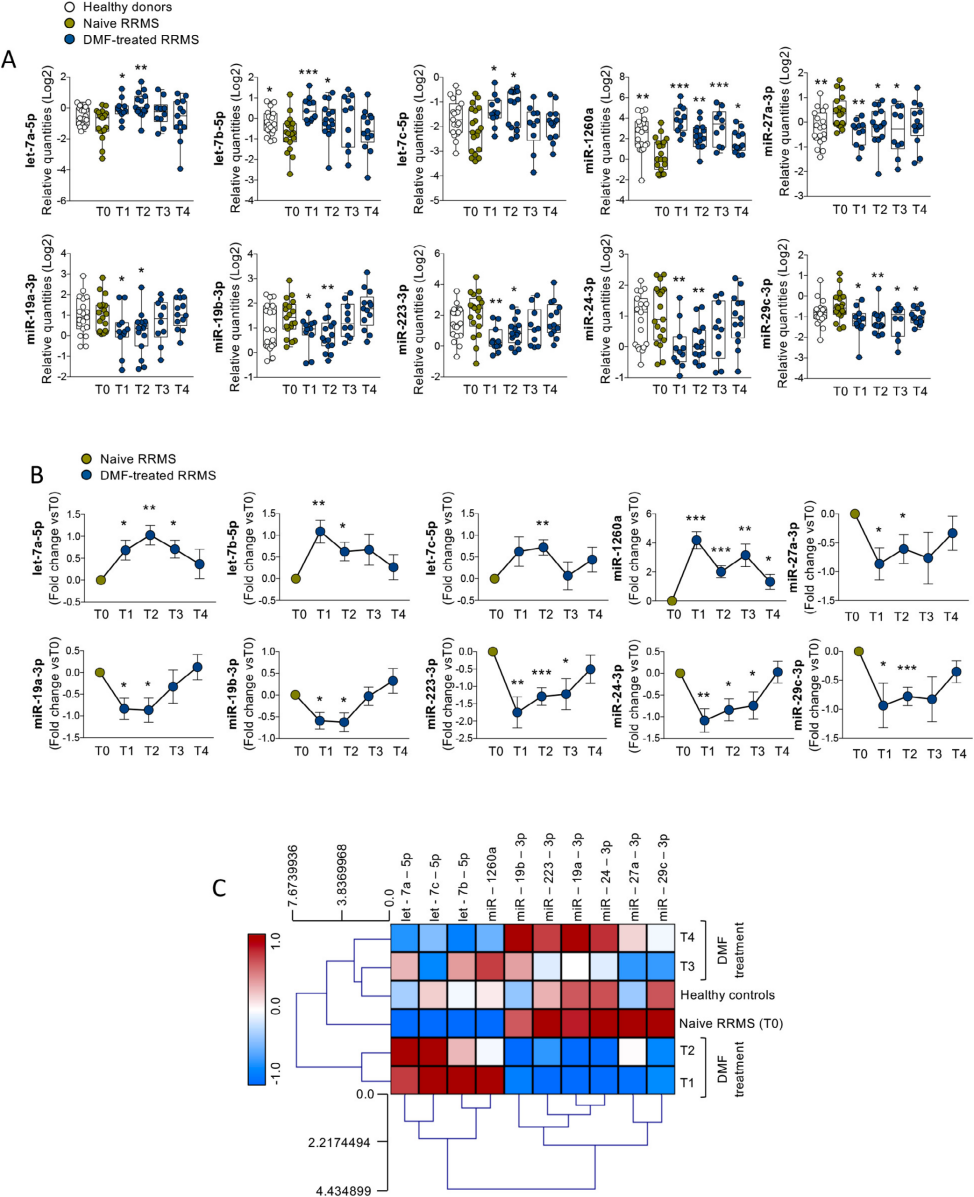
DMF‐dependent modulation of the plasma circulating miRNome. (A) Whisker plots showing blood circulating miRNA levels expressed as relative quantities (Log2) from healthy controls and pwRRMS before (T0) and after 3 (T1), 6 (T2), 12 (T3), and 24 (T4) months of DMF treatment, selected for being significantly different (*t*‐test, *p* < 0.05) in at least two comparisons; asterisks are relative to comparison with the RRMS‐T0 group. (B) Longitudinal behavior of blood circulating miRNAs selected for being significantly different (*t*‐test, *p* < 0.05) in at least two comparisons among different time points; graphs report the fold change versus RRMS‐T0. (C) Hierarchical clustering of healthy controls and pwRRMS before (T0) and after 3 (T1), 6 (T2), 12 (T3), and 24 (T4) months of DMF treatment based on differentially expressed miRNAs, by an ANOVA testing (*p* < 0.05). *p ≤ 0.05, **p ≤ 0.005, ***p ≤ 0.0005.

We collected peripheral blood from the following subjects: (i) pwRRMS treatment‐naïve (*n* = 19) and at four different time points after treatment with DMF (T1, 3 months; T2, 6 months; T3, 12 months; T4, 24 months) and (ii) healthy controls (*n* = 19) sex and age‐matched with pwRRMS. Starting from blood samples, each from a single patient, we then quantified plasma circulating miRNAs, immune cells, and soluble protein factors in parallel, as summarized in Figure 1. The analysis revealed a total of 15 differentially expressed miRNAs in the plasma of pwRRMS upon DMF treatment: (i) Three were differential only after 3 months of DMF treatment (T1 vs disease onset T0) and defined as “transiently modulated”; (ii) Two were differential only after 6 months of DMF treatment (T2 vs disease onset T0) and defined as “delayed”; and (iii) Ten were differential after both 3 and 6 months of DMF treatment (T1 and T2 vs disease onset T0) and thus defined as “persistently modulated”. We decided to strictly focus on this last group, which was composed of all miRNAs being consistently modulated at T1 and T2 (i.e., either up‐regulated or down‐regulated compared with T0 at both time points). In particular, a subset of 4 miRNAs was significantly increased (i.e., let‐7a‐5p, let‐7b‐5p, let‐7c‐5p and miR‐1260a) and another subset of 6 miRNAs was significantly decreased by DMF treatment (i.e., miR‐27a‐3p, miR‐19a‐3p, miR‐19b‐3p, miR‐223‐3p, miR‐24‐3p, miR‐29c‐3p) (Figure 2A). These miRNAs are specifically linked to the response to DMF treatment rather than the disease per se, as shown by the observation that only a minority differed significantly between treatment‐naïve and healthy subjects (Figure 2A). DMF‐dependent changes of the above identified plasma circulating miRNAs were also evident when examining the longitudinal behavior of these miRNAs per single patient (individual fold change) (Figure 2B). In particular, the zenith of miRNA change was consistently occurring at either T1 or T2, while, at later time points, miRNAs tended to return to levels reported before the treatment initiation. While the return to baseline expression at later time points is intriguing, its clinical significance remains uncertain and may reflect a transient response followed by homeostatic adjustment, although this specific aspect should be investigated.

Furthermore, a clustering analysis based on the expression of the DMF‐modulated miRNAs demonstrated that pwRRMS at early time points of DMF treatment (3–6 months T1‐T2) clustered together and far from both healthy controls and pwRRMS before the treatment and at later time points of DMF treatment (12–24 months‐ T3‐T4) (Figure 2C). The correlation among miRNAs belonging to the DMF modulated signature increased with increasing time of DMF treatment. In particular, those miRNAs that were increased by DMF (i.e., let‐7a‐5p, let‐7b‐5p, let‐7c‐5p, and miR‐1260a) were more strongly correlated in a positive way among them and the same was true for those miRNAs significantly decreased by DMF treatment (i.e., miR‐19a‐3p, miR‐19b‐3p, miR‐223‐3p, miR‐24‐3p, miR‐27a‐3p, miR‐29c‐3p); on the other hand, miRNAs belonging to different groups exhibited a more pronounced inverse correlation (e.g., let‐7a‐5p and miR‐19a‐3p) at T3 and T4 (Figure S1A).

### 3.2 The Quantity of Distinct Plasma Circulating miRNAs at Disease Onset Correlates With the Levels of Relevant Soluble Factors Upon DMF Treatment

**FIGURE 3:**
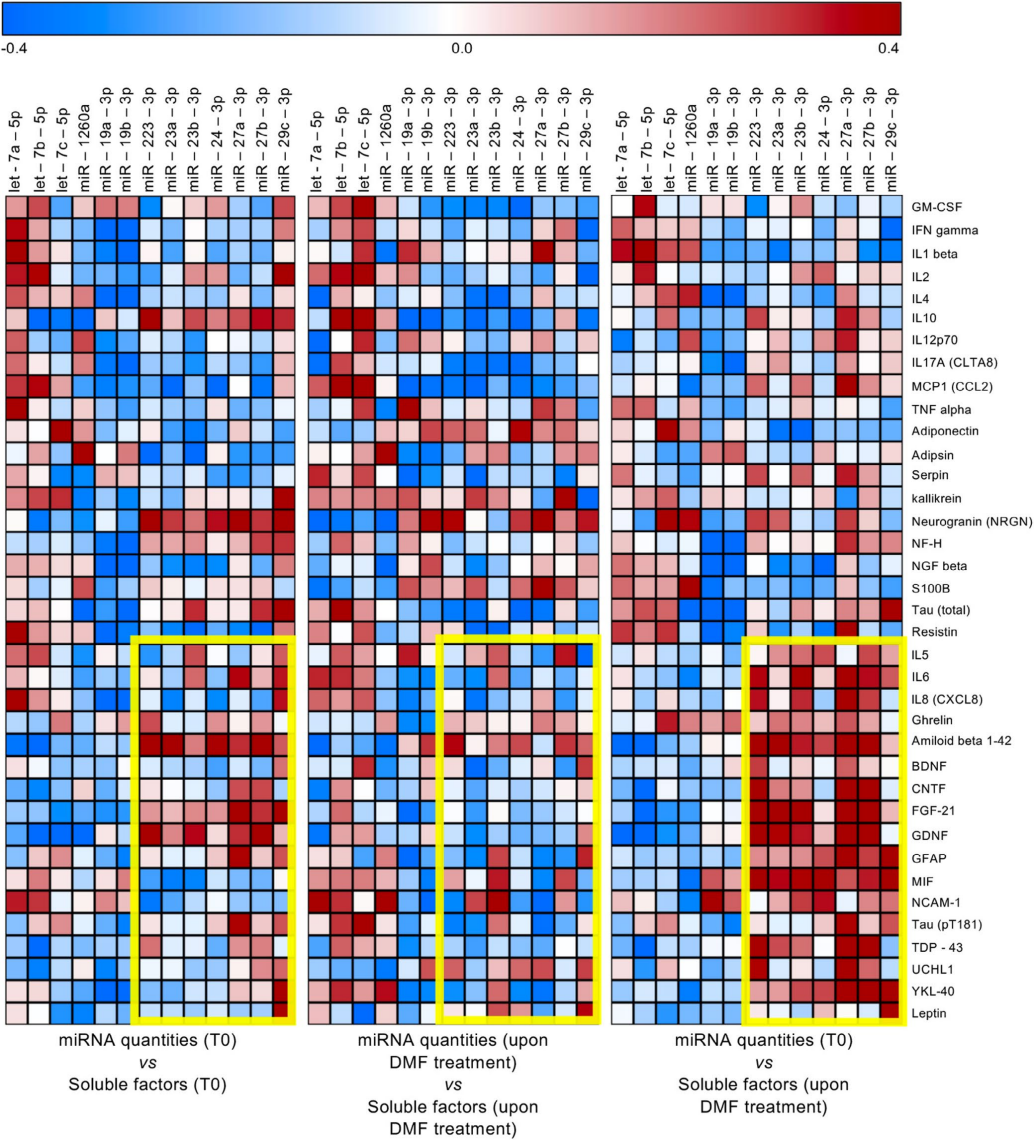
The levels of plasma circulating miRNAs at disease onset correlate with those of soluble factors upon DMF treatment. Heatmap reporting the correlation index (Spearman *r* values, red if positive, blue if negative) of differentially expressed miRNAs and soluble factors in pwRRMS at disease onset (T0) and upon DMF‐treatment.

In healthy controls and pwRRMS before and after DMF treatment, we also evaluated plasma levels of several cytokines (GM‐CSF, IFNγ, IL1β, IL2, IL4, IL5, IL6, IL8, IL10, IL12p70, IL17A, MCP1, TNFα), metabolic factors (ghrelin, leptin, resistin, adiponectin, serpin E1, and adipsin) and markers of neurodegeneration (β amyloid 1–42, BDNF, CNTF, FGF‐21, GDNF, GFAP, kallikrein‐6 (KLK6), MIF, NCAM‐1, neurogranin (NRGN), NF‐H, NGF beta, S100B, Tau (pT181), Tau (total), TDP‐43, UCHL1, YKL‐40 (CHI3L1)), selected for exerting important immunological and neurological functions in humans. In particular, we investigated whether there existed a correlation between plasma circulating miRNAs and those disease‐relevant soluble factors. Intriguingly, the highest positive correlation was found between miRNAs belonging to the identified DMF‐modulated signature (e.g., miR‐223‐3p, miR‐24‐3p, and miR‐27a‐3p), as assessed at disease onset, and the quantities of specific markers of neurodegeneration and brain injury (e.g., GFAP, GDNF, UCHL1, CNTF, YKL40, and TDP‐43), as assessed upon DMF treatment (Figure 3, right panel). This correlation was more pronounced than that revealed between the same parameters evaluated at one single time point (either at disease onset or upon treatment with DMF, Figure 3, left and middle panels).

### 3.3 The Quantity of Plasma Circulating miR‐223‐3p and Let‐7b‐5p at Disease Onset Predicts the Severity of DMF‐Related Lymphopenia

**FIGURE 4:**
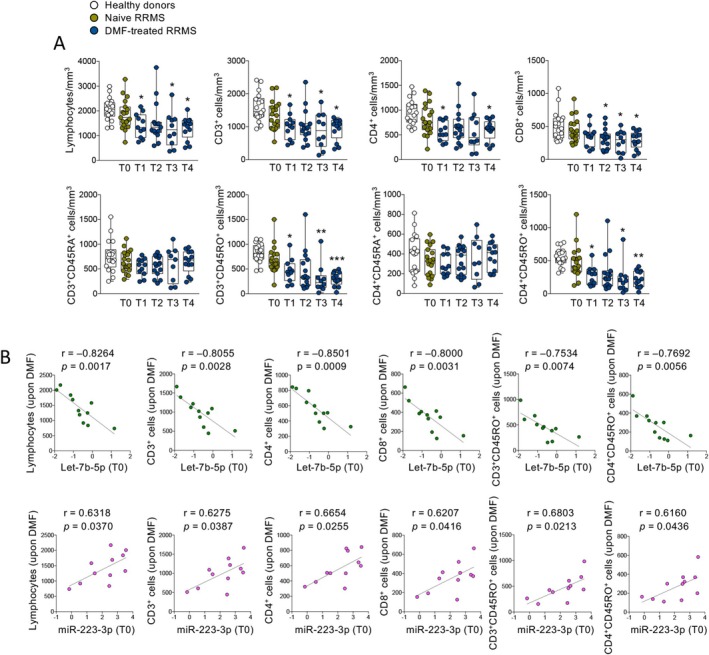
Association of plasma circulating let‐7b‐5p and miR‐223‐3p with DMF‐dependent lymphopenia. (A) Whisker plots showing the number (per microliter) of peripheral blood immune cells from healthy controls and pwRRMS before (T0) and after 3 (T1), 6 (T2), 12 (T3), and 24 (T4) months of DMF treatment, selected for being significantly different (*t*‐test, *p* < 0.05) in at least two comparisons; asterisks are relative to comparison with the RRMS‐T0 group. *p ≤ 0.05; **p ≤ 0.005; and ***p ≤ 0.0005. (B) Scatter plots showing the correlation between plasma circulating let‐7b‐5p (upper panels) and miR‐223‐3p (lower panels) levels at T0 (before starting DMF treatment) and the number (cells/mm^3^) of peripheral blood cell subpopulations at later time points upon DMF treatment in pwRRMS; *r* and *p* values are also reported.

We next evaluated the effect of DMF treatment on the number and percentage of different subsets of peripheral blood immune cells. In order to identify those significantly modulated by disease onset and/or DMF treatment, we selected lymphocyte subpopulations that were significantly differential in at least two comparisons (with all comparisons relative to RRMS onset‐T0) (Figure 4A). Consistent with previous reports [25], we found that the numbers of general lymphocyte populations (total lymphocytes, CD3^+^, CD4^+^, and CD8^+^ T cells) and memory subpopulations (CD45RO^+^) were all decreased by DMF treatment; the percentage of naïve subpopulations (CD45RA^+^) was instead specifically increased by DMF treatment (Figure 4A and Table S2). We then assessed the ability of blood circulating miRNAs (those belonging to the DMF‐modulated signature, but also all the others detected in the study) to predict the reduction in lymphocyte count occurring in DMF‐treated patients. To this aim, we correlated the quantity of circulating miRNAs at disease onset (T0) with the number of lymphocytes reported at later time points upon DMF treatment. Strikingly, the two miRNAs identified as the best predictors of lymphocyte decrease following DMF treatment did actually belong to the DMF‐modulated miRNA signature, i.e., miR‐223‐3p and let‐7b‐5p (Figure 4B). Specifically, we observed that the higher the level of let‐7b‐5p and the lower the level of miR‐223‐3p at disease onset (T0), the lower the blood immune cell count, in particular the number of general lymphocyte populations (total lymphocytes, CD3^+^, CD4^+^, and CD8^+^ T cells) and memory subpopulations (CD3^+^CD45RO^+^ and CD4^+^CD45RO^+^ T cells) reported after DMF treatment (Figure 4B).

In summary, our analysis reveals that the levels of circulating miR‐223‐3p and let‐7b‐5p at disease onset are associated with the reduction in lymphocyte counts induced by DMF treatment.

### 3.4 A Specific Plasma Circulating miRNA Signature Predicts MS Progression and Response to DMF Overtime

**FIGURE 5:**
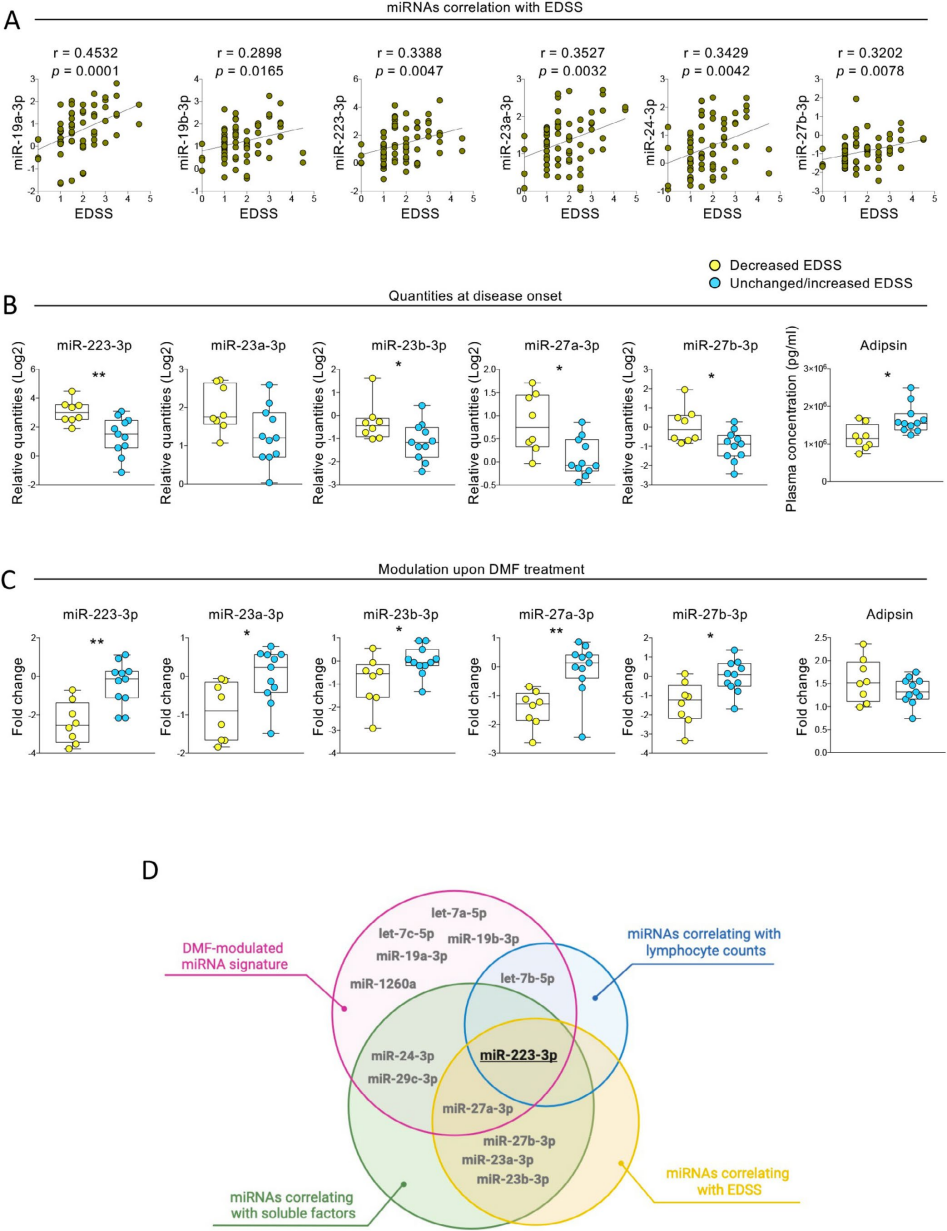
Identification of a specific plasma circulating miRNA signature in the prediction of response to DMF. (A) Scatter plots showing plasma circulating miRNAs positively correlated with EDSS; *r* and *p* values are also reported. (B, C) Whisker plots showing the reported blood circulating miRNAs expressed as relative quantities (Log2) (B) or fold changes (upon DMF treatment) (C), as quantified in pwRRMS stratified on the basis of EDSS change (decreased or unchanged/increased) clinically reported upon DMF treatment. *p ≤ 0.05; **p ≤ 0.005. (D) Venn diagram showing the overlap of the sub‐lists of miRNAs identified in the reported different analyses.

We finally explored the correlation between plasma circulating miRNA levels and the disability of pwRRMS over time during DMF treatment, evaluated as EDSS. Once again, 4 out of 6 of the miRNAs found significantly correlated with EDSS (both at disease onset and upon DMF treatment, Figure 5A) were part of the DMF‐modulated miRNA signature (miR19a‐3p, miR‐19b‐3p, miR‐223‐3p, and miR‐24‐3p).

To assess the ability of plasma circulating miRNAs to predict both clinical response to pharmacological treatment and disease progression, we stratified the patients enrolled in the study based on the change in EDSS over the course of DMF treatment into two groups: (i) good responders (subjects who experienced a decrease in EDSS over the 2 years following treatment initiation, *n* = 8) and (ii) poor responders (subjects who showed no detectable improvement or an increase in EDSS during the follow‐up period, *n* = 11). Among the parameters tested in an unbiased manner, i.e., lymphocyte subpopulations, miRNAs and soluble factors, we identified 5 miRNAs (miR‐223‐3p, miR‐23a‐3p, miR‐23b‐3p, miR‐27a‐3p, and miR‐27b‐3p) and one soluble factor (adipsin) that were able to discriminate between the two groups of subjects (Figure 5B,C). It is important to underline here that both the quantity of these parameters at disease onset (T0) (Figure 5B) and their fold change upon DMF treatment (Figure 5C) were similarly able to significantly discriminate *good* from *poor* DMF responders. Two miRNAs of the DMF‐modulated miRNA group were included in those 5 molecules (i.e., miR‐223‐3p and miR‐27a‐3p) and the other 3 miRNAs belonged to the miRNA cluster containing miR‐27a‐3p, known to be quantitatively and functionally linked in vivo (i.e., miR‐23a‐3p, miR‐23b‐3p, and miR‐27b‐3p) (Figure 5B,C). The overlap for the miRNAs identified across the different analytical settings described (DMF‐modulation, correlation with lymphopenia and soluble factors, and prediction of DMF response) is shown in Figure 5D. Notably, one miRNA, i.e., miR‐223‐3p, consistently appears in all groups of identified miRNAs and is predicted to target key factors involved in different biological processes such as cytokine‐mediated signaling pathway, response to oxidative stress and interleukin (IL)‐6 family signaling (Figure S1B).

In summary, these findings suggest that specific plasma circulating miRNAs may help distinguishing clinical outcomes in pwRRMS undergoing DMF treatment. The consistent presence of miR‐223‐3p across various analytical frameworks highlights its potential as a candidate biomarker not only for predicting treatment response but also for understanding the underlying molecular mechanisms driving both disease progression and therapeutic efficacy.

### 3.5 Validated mRNA Targets of the Identified Plasma Circulating miRNAs Are Enriched for IL‐6 Signaling Pathway

**FIGURE 6:**
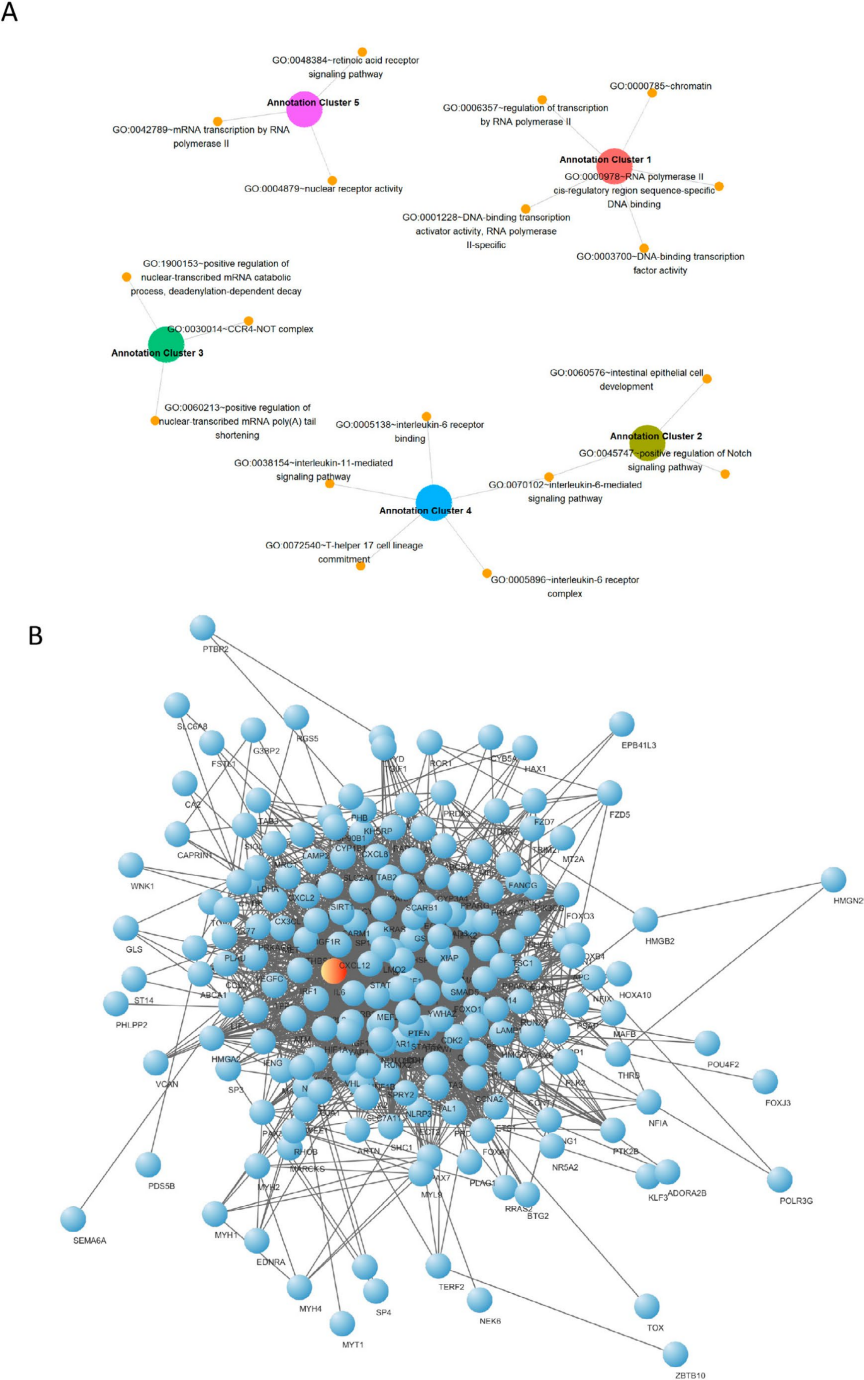
Functional Annotation Clustering and Protein–Protein Interaction network of miRNA‐targeted mRNAs. (A) Gene Ontology (GO) enrichment analysis (DAVID) of mRNA targets identified for miR‐223‐3p, miR‐23a‐3p, miR‐27b‐3p, miR‐27a‐3p, and miR‐23b‐3p. The top 5 enriched annotation clusters are displayed based on their enrichment scores. GO terms are grouped into clusters, each annotated with relevant biological processes. (B) Protein–Protein Interaction (PPI) network analysis using STRING and Cytoscape of the mRNA targets of the above‐mentioned miRNAs. IL‐6 (the major hub based on the betweenness centrality score) is highlighted in red.

After identifying the miRNAs associated with a good clinical response to DMF, we explored their biological implications by importing the cumulative list of their targeted mRNAs (*n* = 211, with strong experimental evidence from miRTarBase, https://miRTarBase.cuhk.edu.cn/) into the DAVID web interface. Gene ontology (GO) analysis of these 211 targets revealed significant enrichment in pathways related to the positive regulation of Notch signaling, retinoic acid receptor signaling (clusters 2 and 5), and IL‐6‐dependent signaling (clusters 2 and 4) (Figure 6A; File S1). Pathway analysis highlighted the enrichment of several terms, and beyond general ones like infection‐ and cancer‐related molecular cascades, we specifically observed the enrichment of “Nuclear events mediated by NFE2L2” and “KEAP1‐NFE2L2 pathway” (File S2). Since NRF2 (NFE2L2) is a direct target of DMF, the enrichment of NRF2‐related pathways by these miRNAs suggests they may play a key role in regulating the therapeutic response to DMF. Furthermore, a de novo network analysis performed in Cytoscape identified IL‐6 (known to be involved in the response of pwRRMS to DMF) [26, 27] as the major hub based on the betweenness centrality score (Figure 6B; File S3).

These results highlight the involvement of key signaling pathways, especially IL‐6, in the response to DMF. These findings provide a deeper understanding of the pathways involved in DMF response and suggest that the identified miRNAs may contribute to the regulation of biological pathways relevant to DMF response; however, further functional studies are needed to determine whether DMF directly exerts its effects through modulation of these miRNAs.

## 4 Discussion

The standard option treatment for pwRRMS is based on several DMTs, and, among them, DMF has shown clinical efficacy possibly through its immunomodulatory and antioxidant action in the CNS [28]. Despite the beneficial effects of DMF, clinical and radiological outcomes remain highly variable among pwRRMS and the prediction of disease progression is still highly challenging [29]. Thereby, there remains an unmet medical need to identify objective, tractable, and easily accessible biomarkers of MS activity, able to anticipate either disease progression or response to therapy, thus allowing “tailored” therapeutic decisions based on the unique molecular characteristics of each patient [30, 31, 32]. In particular, it would be relevant to predict who will either optimally or sub‐optimally respond to DMTs, including DMF, in order to anticipate disability worsening. This approach could prove useful in aiding the therapeutic choice in a more selective and powerful way, allowing physicians to undertake therapeutic paths as early as possible, improving patient outcomes.

Changes in plasma circulating miRNAs as possible tractable biomarkers of individual response to DMTs, such as DMF, remain to be unambiguously defined, and their connection with the immunometabolic derangement that characterizes MS pathogenesis is still poorly explored. Despite the observed alteration in the levels of plasma circulating miRNAs and immunometabolic factors, which may be instigated by either the inflammatory imbalance or the neurodegenerative process, our results align with a framework where this composite signature of molecules and their complex interrelation may function as a rheostat of the heterogeneous (i.e., neurological and immune) pathogenetic nature of MS.

In summary, our study supports the hypothesis that the DMF‐modulated miRNA signature quantified at disease onset, before initiation of DMF treatment, may anticipate specific molecular responses to the therapeutic treatment. Notwithstanding the clinical changes followed in the study are short‐term and may be confounded by the variability of outcomes, our findings suggest the potential of a specific plasma circulating miRNA signature, particularly miR‐223‐3p, miR‐23a‐3p, miR‐23b‐3p, miR‐27a‐3p, and miR‐27b‐3p as predictive biomarkers for MS progression and response to DMF treatment. These miRNAs not only show a significant correlation with changes in disability over time, as assessed by EDSS, but also distinguish good responders from poor responders to DMF therapy. Given that DMF primarily suppresses disease activity rather than directly improving clinical disability, the observed EDSS reductions in some patients may reflect an indirect effect of this drug in early inflammatory control, especially in the context of early‐stage MS. Importantly, their predictive capacity both at disease onset and throughout treatment may become instrumental in forecasting the course of MS, allowing for earlier interventions and more personalized therapeutic strategies. The ability to anticipate clinical outcomes based on miRNA profiles could have profound implications for improving patient management, optimizing treatment decisions, and ultimately enhancing long‐term prognosis in MS.

The gene ontology and pathway analyses of miRNA targets associated with a positive response to DMF highlight key biological processes, such as Notch signaling, retinoic acid receptor signaling, and IL‐6 dependent pathways, which may be crucial in modulating therapeutic outcomes. The enrichment of NRF2‐related pathways, given its direct interaction with DMF, further supports the role of these miRNAs in monitoring treatment response. At the biological level, our findings open the hypothesis that DMF effects may also be mediated through the modulation of miRNAs influencing pathways related to their mRNA targets. The actual existence of this proposed regulatory mechanism will have to be tested in further studies employing animal models of therapeutic mechanisms.

Our effort allowed us to unveil an intriguing, unexpected pattern, indicating that the highest correlation subsisted between the miRNA levels at the time of treatment initiation and relevant clinical parameters *upon* DMF treatment. Despite further experimental work being required to elucidate the aetio‐pathological significance of these findings, our pathway analysis has unveiled the potential link between the dysregulation of the identified miRNA signature and biological processes highly relevant for MS pathogenesis and DMF efficacy, such as the IL‐6 signaling pathway and the NRF‐2 dependent antioxidant response.

In conclusion, the identification of novel biomarkers has the potential to improve the efficacy of MS treatment, preventing therapeutic failures and unnecessary side effects, and providing valuable insights into the development of personalized medicine, with significant benefit for the patients and the healthcare system.

## Author Contributions

Giuseppe Matarese and Paola de Candia conceived and designed the study. Maria Mottola, Giorgia Teresa Maniscalco, Antonio Luca Spiezia, Roberta Lanzillo, Vincenzo Brescia Morra, Gianmarco Abbadessa, Simona Bonavita, Alessia Castiello were responsible for the recruitment, acquisition, and analysis of related clinical data of MS and healthy control subjects belonging to the study cohort; Silvia Garavelli performed the microRNA profiling. Fortunata Carbone, Alessandra Colamatteo, Teresa Micillo, Clorinda Fusco, Francesco Perna, Claudia Russo, Federica Garziano, Claudia La Rocca, Claudio Procaccini performed the immunometabolic factor quantification and immunophenotypic analysis. Paola de Candia and Gianmarco Abbadessa conducted statistical and bio‐informatic analyses, and Fortunata Carbone helped with data analysis. Paola de Candia and Fortunata Carbone wrote the manuscript, and Paola de Candia, Fortunata Carbone, and Claudio Procaccini edited it. Paola de Candia and Giuseppe Matarese concurred in the final data interpretation, and all authors contributed to its editing and gave final approval of the version to be published.

## Conflicts of Interest

G.M. reports receiving research grant support from Merck, Biogen, and Novartis, and advisory board fees from Merck, Biogen, Novartis, and Roche. R.L. received personal compensation for speaking or consultancy from Biogen, Alexion, Sanofi, Merck, Bristol Myers Squibb, Janssen, Novartis, Amgen, and Roche. V.B.M. has received research grants from the Italian MS Society and Roche, and honoraria from Bayer, Biogen, Merck, Mylan, Novartis, Roche, Sanofi‐Genzyme, and Teva. S.B. reports receiving payment or honoraria for lectures, presentations, and speakers bureaus from Roche, Novartis, Biogen, Merck‐Serono, Alexion, Horizon, Janssen‐Cilag, BMS, Viatris, and support for attending meetings and/or travel from Roche, Novartis, Biogen, Merck‐Serono, Alexion, Horizon, Janssen‐Cilag, BMS, Viatris. G.A. reports receiving payment or honoraria for lectures, presentations, and speakers bureaus from Lundbeck. None of the other authors declare any conflicts of interest.

## Supporting information


**Figure S1.** (A) Heatmap reporting the correlation index (Spearman *r* values, red if positive, blue if negative) of differentially expressed miRNAs, in healthy controls and pwRRMS before (T0) and after 3, 6 12, and 24 months of DMF‐treatment (T1, T2, T3, and T4 respectively). (B) STRING analysis showing the functional network of proteins encoded by selected transcripts targeted by the hit miRNA of Figure 5D (miR‐223‐3p). Cytokine‐mediated signaling pathway (GO:0019221) is reported in red, response to oxidative stress (GO:0006979) is reported in blue, and interleukin 6 family signaling (HSA: 6783589) is reported in yellow.

**File S1.** Supplementary File 1.

**File S2.** Supplementary File 2.

**File S3.** Supplementary File 3.

**Table S1.** Demographic and clinical data of pwRRMS and healthy controls.

**Table S2.** Absolute number (cells/mm3) and percentage (of the lymphocyte count, indicated in parentheses) of different immune cell subpopulations in peripheral blood of healthy controls and pwRRMS before (T0) and after (T1, 3 months; T2, 6 months, T3, 12 months; T4, 24 months) DMF treatment. Data are presented as mean ± SD.

## Data Availability

The data that support the findings of this study are available from the corresponding authors upon reasonable request.

## References

[1] D. Jakimovski , S. Bittner , R. Zivadinov , et al., “Multiple Sclerosis,” Lancet 403 (2024): 183–202. 37949093 10.1016/S0140-6736(23)01473-3

[2] M. Filippi , A. Bar‐Or , F. Piehl , et al., “Multiple Sclerosis,” Nature Reviews Disease Primers 4, no. 43 (2018). 10.1038/s41572-018-0041-4 30410033

[3] C. Confavreux , S. Vukusic , T. Moreau , and P. Adeleine , “Relapses and Progression of Disability in Multiple Sclerosis,” New England Journal of Medicine 343 (2000): 1430–1438. 11078767 10.1056/NEJM200011163432001

[4] G. Giovannoni , V. Popescu , J. Wuerfel , et al., “Smouldering Multiple Sclerosis: The ‘Real MS’,” Therapeutic Advances in Neurological Disorders 15 (2022): 17562864211066751. 35096143 10.1177/17562864211066751 PMC8793117

[5] S. L. Hauser and B. A. C. Cree , “Treatment of Multiple Sclerosis: A Review,” American Journal of Medicine 133 (2020): 1380–1390.e2. 32682869 10.1016/j.amjmed.2020.05.049 PMC7704606

[6] B. Narapureddy and D. Dubey , “Clinical Evaluation of Dimethyl Fumarate for the Treatment of Relapsing‐Remitting Multiple Sclerosis: Efficacy, Safety, Patient Experience and Adherence,” Patient Preference and Adherence 13 (2019): 1655–1666. 31631980 10.2147/PPA.S187529 PMC6778444

[7] G. Bresciani , F. Manai , S. Davinelli , P. Tucci , L. Saso , and M. Amadio , “Novel Potential Pharmacological Applications of Dimethyl Fumarate‐An Overview and Update,” Frontiers in Pharmacology 14 (2023): 1264842. 37745068 10.3389/fphar.2023.1264842 PMC10512734

[8] M. Mirabella , L. Prosperini , M. Lucchini , et al., “Safety and Efficacy of Dimethyl Fumarate in Multiple Sclerosis: An Italian, Multicenter, Real‐World Study,” CNS Drugs 32 (2018): 963–970. 30022464 10.1007/s40263-018-0543-3

[9] R. H. Thomas and R. A. Wakefield , “Oral Disease‐Modifying Therapies for Relapsing‐Remitting Multiple Sclerosis,” American Journal of Health‐System Pharmacy 72 (2015): 25–38. 25511835 10.2146/ajhp140023

[10] R. J. Fox , D. H. Miller , J. T. Phillips , et al., “Placebo‐Controlled Phase 3 Study of Oral BG‐12 or Glatiramer in Multiple Sclerosis,” New England Journal of Medicine 367 (2012): 1087–1097. 22992072 10.1056/NEJMoa1206328

[11] R. Gold , L. Kappos , D. L. Arnold , et al., “Placebo‐Controlled Phase 3 Study of Oral BG‐12 for Relapsing Multiple Sclerosis,” New England Journal of Medicine 367 (2012): 1098–1107. 22992073 10.1056/NEJMoa1114287

[12] A. Hammer , A. Waschbisch , K. Kuhbandner , et al., “The NRF2 Pathway as Potential Biomarker for Dimethyl Fumarate Treatment in Multiple Sclerosis,” Annals of Clinical Translational Neurology 5 (2018): 668–676. 29928650 10.1002/acn3.553 PMC5989754

[13] C. Procaccini , S. Garavelli , F. Carbone , et al., “Signals of Pseudo‐Starvation Unveil the Amino Acid Transporter SLC7A11 as Key Determinant in the Control of Treg Cell Proliferative Potential,” Immunity 54 (2021): 1543–1560. e6. 34004141 10.1016/j.immuni.2021.04.014

[14] S. K. Yadav , D. Soin , K. Ito , and S. Dhib‐Jalbut , “Insight Into the Mechanism of Action of Dimethyl Fumarate in Multiple Sclerosis,” Journal of Molecular Medicine (Berlin, Germany) 97 (2019): 463–472. 30820593 10.1007/s00109-019-01761-5

[15] E. E. Longbrake and A. H. Cross , “Dimethyl Fumarate Associated Lymphopenia in Clinical Practice,” Multiple Sclerosis 21 (2015): 796–797. 25432948 10.1177/1352458514559299 PMC4426020

[16] E. E. Longbrake , R. T. Naismith , B. J. Parks , G. F. Wu , and A. H. Cross , “Dimethyl Fumarate‐Associated Lymphopenia: Risk Factors and Clinical Significance,” Multiple Sclerosis Journal: Experimental, Translational and Clinical 1, no. 16 (2015): 1–8. 10.1177/2055217315596994 PMC4636217 26550483

[17] G. Boffa , N. Bruschi , M. Cellerino , et al., “Fingolimod and Dimethyl‐Fumarate‐Derived Lymphopenia Is not Associated With Short‐Term Treatment Response and Risk of Infections in a Real‐Life MS Population,” CNS Drugs 34 (2020): 425–432. 32193826 10.1007/s40263-020-00714-8

[18] R. J. Gieselbach , A. H. Muller‐Hansma , M. T. Wijburg , et al., “Progressive Multifocal Leukoencephalopathy in Patients Treated With Fumaric Acid Esters: A Review of 19 Cases,” Journal of Neurology 264 (2017): 1155–1164. 28536921 10.1007/s00415-017-8509-9

[19] D. P. Bartel , “MicroRNAs: Target Recognition and Regulatory Functions,” Cell 136 (2009): 215–233. 19167326 10.1016/j.cell.2009.01.002 PMC3794896

[20] K. Chandan , M. Gupta , and M. Sarwat , “Role of Host and Pathogen‐Derived MicroRNAs in Immune Regulation During Infectious and Inflammatory Diseases,” Frontiers in Immunology 10 (2020): 3081. 32038627 10.3389/fimmu.2019.03081 PMC6992578

[21] M. P. Mycko and S. E. Baranzini , “microRNA and Exosome Profiling in Multiple Sclerosis,” Multiple Sclerosis 26 (2020): 599–604. 31965891 10.1177/1352458519879303 PMC7160025

[22] K. Regev , B. C. Healy , A. Paul , et al., “Identification of MS‐Specific Serum miRNAs in an International Multicenter Study,” Neurology Neuroimmunology & Neuroinflammation 5 (2018): e491. 30175165 10.1212/NXI.0000000000000491 PMC6117192

[23] R. Gandhi , B. Healy , T. Gholipour , et al., “Circulating microRNAs as Biomarkers for Disease Staging in Multiple Sclerosis,” Annals of Neurology 73 (2013): 729–740. 23494648 10.1002/ana.23880

[24] C. C. Hemond , B. C. Healy , S. Tauhid , et al., “MRI Phenotypes in MS: Longitudinal Changes and miRNA Signatures,” Neurology Neuroimmunology & Neuroinflammation 6 (2019): e530. 30800720 10.1212/NXI.0000000000000530 PMC6384020

[25] E. E. Longbrake , M. J. Ramsbottom , C. Cantoni , L. Ghezzi , A. H. Cross , and L. Piccio , “Dimethyl Fumarate Selectively Reduces Memory T Cells in Multiple Sclerosis Patients,” Multiple Sclerosis 22 (2016): 1061–1070. 26459150 10.1177/1352458515608961 PMC4829494

[26] R. Li , A. Rezk , M. Ghadiri , et al., “Dimethyl Fumarate Treatment Mediates an Anti‐Inflammatory Shift in B Cell Subsets of Patients With Multiple Sclerosis,” Journal of Immunology 198 (2017): 691–698. 10.4049/jimmunol.1601649 27974457

[27] H. Wilms , J. Sievers , U. Rickert , M. Rostami‐Yazdi , U. Mrowietz , and R. Lucius , “Dimethylfumarate Inhibits Microglial and Astrocytic Inflammation by Suppressing the Synthesis of Nitric Oxide, IL‐1beta, TNF‐Alpha and IL‐6 in an In‐Vitro Model of Brain Inflammation,” Journal of Neuroinflammation 7 (2010): 30. 20482831 10.1186/1742-2094-7-30 PMC2880998

[28] A. J. Thompson , S. E. Baranzini , J. Geurts , B. Hemmer , and O. Ciccarelli , “Multiple Sclerosis,” Lancet 391 (2018): 1622–1636. 29576504 10.1016/S0140-6736(18)30481-1

[29] D. Rotstein and X. Montalban , “Reaching an Evidence‐Based Prognosis for Personalized Treatment of Multiple Sclerosis,” Nature Reviews. Neurology 15 (2019): 287–300. 30940920 10.1038/s41582-019-0170-8

[30] V. K. Harris and S. A. Sadiq , “Biomarkers of Therapeutic Response in Multiple Sclerosis: Current Status,” Molecular Diagnosis & Therapy 18 (2014): 605–617. 25164543 10.1007/s40291-014-0117-0 PMC4245485

[31] D. Paolicelli , A. Manni , M. D'Onghia , et al., “Lymphocyte Subsets as Biomarkers of Therapeutic Response in Fingolimod Treated Relapsing Multiple Sclerosis Patients,” Journal of Neuroimmunology 303 (2017): 75–80. 28043652 10.1016/j.jneuroim.2016.12.012

[32] S. Medina , N. Villarrubia , S. Sainz de la Maza , et al., “Optimal Response to Dimethyl Fumarate Associates in MS With a Shift From an Inflammatory to a Tolerogenic Blood Cell Profile,” Multiple Sclerosis 24 (2018): 1317–1327. 28653862 10.1177/1352458517717088

