# Supplementary material for: Plasma microRNA Signature as Predictive Marker of Clinical Response to Therapy During Multiple Sclerosis
Source: Ann Clin Transl Neurol. 2025 Jun 11;12(8):1595–607. doi: 10.1002/acn3.70093 (PMC12343307; doi:10.1002/acn3.70093)
Supplement: Supplementary file 1 — Figure S1. (A) Heatmap reporting the correlation index (Spearman r values, red if positive, blue if negative) of differentially expressed miRNAs, in healthy controls and pwRRMS before (T0) and after 3, 6 12, and 24 months of DMF‐treatment (T1, T2, T3, and T4 respectively). (B) STRING analysis showing the functional network of proteins encoded by selected transcripts targeted by the hit miRNA of Figure 5D (miR‐223‐3p). Cytokine‐mediated signaling pathway (GO:0019221) is reported in red, response to oxidative stress (GO:0006979) is reported in blue, and interleukin 6 family signaling (HSA: 6783589) is reported in yellow. [file ACN3-12-1595-s005.pdf]

A

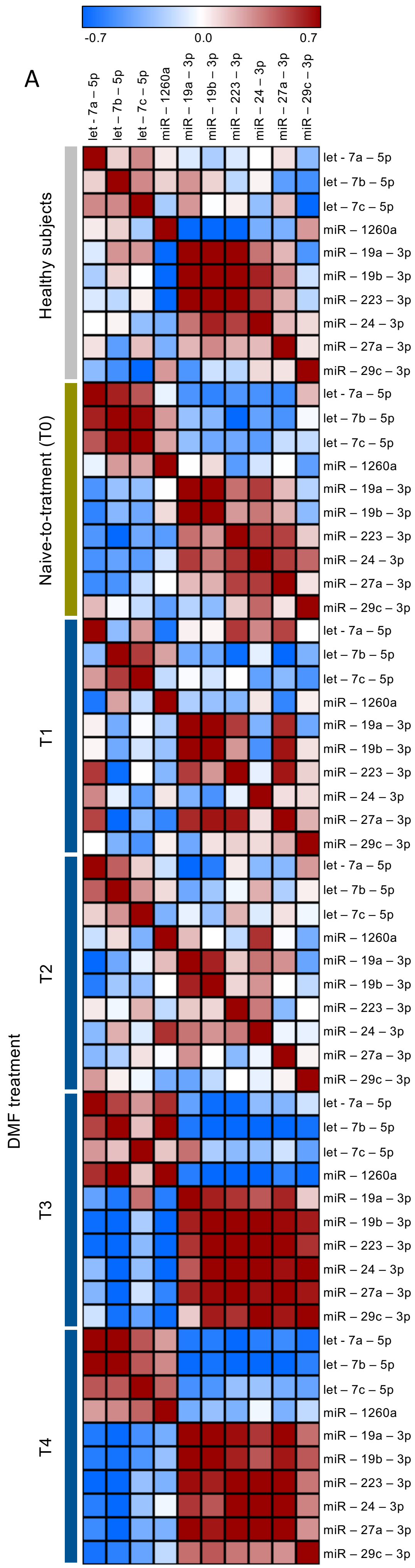

B

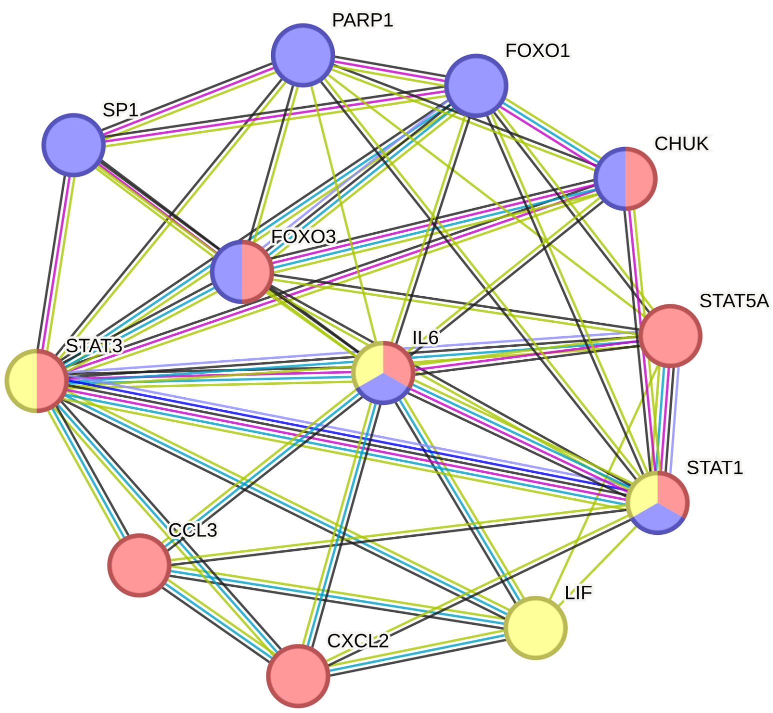

Supplementary Figure 1. (A) Heatmap reporting the correlation index (Spearman r values, red if positive, blue if negative) of differentially expressed miRNAs, in healthy controls and pwRRMS before (T0) and after 3, 6 12 and 24 months of DMF-treatment (T1, T2, T3 and T4 respectively). (**B**) STRING analysis showing the functional network of proteins encoded by selected transcripts targeted by the hit miRNA of **Figure 5D** (miR-223-3p). Cytokine-mediated signaling pathway (GO:0019221) is reported in red, response to oxidative stress (GO:0006979) is reported in blue, and interleukin 6 family signaling (HSA: 6783589) is reported in yellow.
