# Supplementary material for: Plasma microRNA Signature as Predictive Marker of Clinical Response to Therapy During Multiple Sclerosis
Source: Ann Clin Transl Neurol. 2025 Jun 11;12(8):1595–607. doi: 10.1002/acn3.70093 (PMC12343307; doi:10.1002/acn3.70093)
Supplement: Supplementary file 3 — File S2. Supplementary File 2. [file ACN3-12-1595-s006.pdf]

Annotation Cluster 1Enrichment Score: 15.9097684860275
CategoryTermCount%PValueGenesList TotalPop HitsPop TotalFold EnrichmentBonferroniBenjaminiFDR
REACTOME_PATHWAYR-HSA-74160~Gene expression (Transcription)17318.287526427061313.8431217155898595E-17RB1, ZNF175, CCNK, CCNT2, CCNT1, EHMT2, UBE2D1, PRDM1, GLS, CCND3, SESN3, CCND1, MYC, CHEK2, ZNF208, NAMPT, SESN2, CDC27, EP300, RNF111, ZNF682, MEF2C, [illegible]
REACTOME_PATHWAYR-HSA-73857~RNA Polymerase II Transcription15916.8076109936575056.846431751410375E-17RB1, ZNF175, CCNK, CCNT2, CCNT1, EHMT2, UBE2D1, PRDM1, GLS, CCND3, SESN3, CCND1, MYC, CHEK2, ZNF208, NAMPT, SESN2, CDC27, EP300, RNF111, ZNF682, MEF2C, [illegible]
REACTOME_PATHWAYR-HSA-212436~Generic Transcription Pathway14715.539112050739967.088365765862132E-16RB1, ZNF175, CCNK, CCNT2, CCNT1, EHMT2, UBE2D1, PRDM1, GLS, CCND3, SESN3, CCND1, MYC, CHEK2, ZNF208, NAMPT, SESN2, CDC27, EP300, RNF111, ZNF682, MEF2C, [illegible]

Annotation Cluster 2Enrichment Score: 8.584630178577942
CategoryTermCount%PValueGenesList TotalPop HitsPop TotalFold EnrichmentBonferroniBenjaminiFDR
KEGG_PATHWAYhsa05161:Hepatitis B343.59408033826638467.698464947038366E-10RB1, CXCL8, SRC, PIK3R1, MAPK9, NRAS, MYC, E2F1, PTK2B, EP300, JAK1, STAT5A, MAP2K4, STAT5B, SMAD4, SMAD3, CHUK, APAF1, STAT1, STAT3, MAPK14, YWHAZ, TGFBR1, CCNA2, IL6, CREB1, CDK2, FAS, [illegible]
KEGG_PATHWAYhsa05163:Human cytomegalovirus infection404.22832980972515852.935647318837443E-9RB1, GSK3B, CXCL8, SRC, GNAI3, ITPR3, PIK3R1, EGFR, CX3CL1, CRKL, NRAS, CCND1, MYC, GNA12, CCL3, E2F1, PTK2B, IL6R, PRKACB, JAK1, CHUK, STAT3, TSC1, MAPK14, GNG12, IL6, [illegible]
KEGG_PATHWAYhsa05167:Kaposi sarcoma-associated herpesvirus infection363.80549682875264277.798327110414688E-9RB1, GSK3B, CXCL8, SRC, ITPR3, PIK3R1, HIF1A, CXCL2, PIK3CG, MAPK9, PREX1, NRAS, ZFP36, CCND1, MYC, E2F1, EP300, RPS27A, JAK1, MAP2K4, CHUK, STAT1, STAT3, [illegible]

Annotation Cluster 3Enrichment Score: 7.6512400840340495
CategoryTermCount%PValueGenesList TotalPop HitsPop TotalFold EnrichmentBonferroniBenjaminiFDR
REACTOME_PATHWAYR-HSA-1643685~Disease17918.9217758985200854.953540708199701E-12RB1, CCNK, CCNT2, CCNT1, KDM1A, CTNND1, UBE2D1, IGF1R, PPP1CB, CCND3, GJA1, CCND1, MPRIP, RPS18, CDH1, MYC, CDC27, CYP1B1, EP300, TRIM27, PRKACB, IL6R, MAP2K4, ALG9, TPM3, [illegible]
REACTOME_PATHWAYR-HSA-5663205~Infectious disease11011.6279069767441851.7475135515137837E-9RB1, CCNK, CCNT2, CCNT1, KDM1A, CTNND1, IGF1R, PPP1CB, GJA1, RPS18, CDH1, EP300, TRIM27, PRKACB, IL6R, MAP2K4, RPS5, RPL22, MED4, RUNX1, EEF1A1, NUP93, ADAM17, [illegible]
REACTOME_PATHWAYR-HSA-9824446~Viral Infection Pathways848.8794926004228342.9006966915063605E-6RB1, ITGB1, GSK3B, CCNK, CCNT2, CCNT1, KDM1A, CLTA, IGF1R, RPS4X, PPP1CB, GJA1, MED14, XPO1, MED13, AP1G1, RPS18, EP300, IL6R, JAK1, HSP90AA1, PDCD6IP, PARP1, [illegible]
REACTOME_PATHWAYR-HSA-9679506~SARS-CoV Infections495.1797040169133199.890108489503807E-6RB1, ITGB1, GSK3B, KDM1A, RPS4X, GJA1, RPS18, EP300, IL6R, JAK1, HSP90AA1, NUP133, CHUK, RPS5, YWHAZ, RUNX1, NUP93, EEF1A1, FKBP4, SOS1, DDX5, RPN2, ATP1A1, GANAB, [illegible]

Annotation Cluster 4Enrichment Score: 6.3529626239736325
CategoryTermCount%PValueGenesList TotalPop HitsPop TotalFold EnrichmentBonferroniBenjaminiFDR
KEGG_PATHWAYhsa05220:Chronic myeloid leukemia232.4312896405919666.113351814321562E-10RB1, STAT5A, STAT5B, SMAD4, SMAD3, CHUK, SHC1, PTPN11, PIK3R1, TGFBR1, CRKL, RUNX1, NRAS, CCND1, MYC, MDM2, E2F1, GRB2, KRAS, RAF1, SOS1, TP53, [illegible]
KEGG_PATHWAYhsa05161:Hepatitis B343.59408033826638467.698464947038366E-10RB1, CXCL8, SRC, PIK3R1, MAPK9, NRAS, MYC, E2F1, PTK2B, EP300, JAK1, STAT5A, MAP2K4, STAT5B, SMAD4, SMAD3, CHUK, APAF1, STAT1, STAT3, MAPK14, YWHAZ, TGFBR1, CCNA2, IL6, CREB1, CDK2, FAS, [illegible]
KEGG_PATHWAYhsa05215:Prostate cancer252.64270613107822433.1794288886066588E-9RB1, GSK3B, PTEN, PIK3R1, FOXO1, EGFR, HSP90B1, IGF1R, NRAS, CCND1, PLAU, E2F1, EP300, HSP90AA1, CHUK, IGF1, ZEB1, CREB1, CDK2, MDM2, GRB2, KRAS, RAF1, SOS1, [illegible]
KEGG_PATHWAYhsa05225:Hepatocellular carcinoma323.3826638477801273.443841569198815E-8RB1, SMARCD1, GSK3B, SHC1, LRP5, PTEN, PIK3R1, EGFR, IGF1R, NRAS, CCND1, MYC, DPF1, E2F1, SMAD2, SMAD4, SMAD3, PHF10, FZD5, FZD7, FZD9, WNT9B, TGFBR1, APC, GRB2, KRAS, RAF1, [illegible]
KEGG_PATHWAYhsa05219:Bladder cancer151.58562367864693446.942939827570999E-8RB1, CXCL8, SRC, THBS1, EGFR, NRAS, RPS6KA5, CCND1, CDH1, MYC, MDM2, E2F1, KRAS, RAF1, [illegible]
KEGG_PATHWAYhsa05205:Proteoglycans in cancer353.6997885835095147.580793417923135E-8ITGB1, DDX5, SRC, ITPR3, PIK3R1, THBS1, HIF1A, EGFR, IGF1R, PPP1CB, NRAS, CCND1, PLAU, MYC, FLNA, PRKACB, VAV3, SMAD2, FZD5, FZD7, STAT3, FZD9, WNT9B, RRAS2, PTPN11, IGF1, MAPK14, [illegible]
KEGG_PATHWAYhsa05226:Gastric cancer293.06553911205074 9.569185552174364E-8RB1, GSK3B, ABCB1, SHC1, LRP5, PIK3R1, EGFR, NRAS, RXRA, CCND1, CDH1, MYC, E2F1, SMAD2, SMAD4, SMAD3, FZD5, FZD7, FZD9, WNT9B, TGFBR1, APC, CDK2, GRB2, KRAS, RAF1, SOS1, MET, [illegible]
KEGG_PATHWAYhsa01522:Endocrine resistance232.4312896405919669.57861943056722E-8RB1, NOTCH2, NOTCH1, SHC1, SRC, PIK3R1, IGF1, MAPK14, EGFR, IGF1R, MAPK9, NRAS, CCND1, SP1, CARM1, MDM2, E2F1, GRB2, KRAS, RAF1, SOS1, TP53, PRKACB, [illegible]
KEGG_PATHWAYhsa05212:Pancreatic cancer192.008456659619456.37121592489735E-7RB1, SMAD2, SMAD4, SMAD3, CHUK, STAT1, STAT3, PIK3R1, EGFR, TGFBR1, MAPK9, RAD51, CCND1, E2F1, KRAS, RAF1, TP53, JAK1, [illegible]
KEGG_PATHWAYhsa05224:Breast cancer272.8541226215644829.417734782000627E-7RB1, NOTCH2, GSK3B, NOTCH1, SHC1, LRP5, PTEN, PIK3R1, EGFR, IGF1R, NRAS, CCND1, MYC, E2F1, HES1, FZD5, FZD7, FZD9, WNT9B, IGF1, APC, SP1, GRB2, KRAS, RAF1, SOS1, [illegible]
KEGG_PATHWAYhsa05223:Non-small cell lung cancer181.9027484143763214 1.3824957988054916E-6RB1, STAT5A, RET, STAT5B, STAT3, PIK3R1, FOXO3, EGFR, NRAS, RXRA, CCND1, E2F1, GRB2, KRAS, RAF1, SOS1, MET, [illegible]
KEGG_PATHWAYhsa05210:Colorectal cancer192.008456659619454.210962693987519E-6SMAD2, GSK3B, SMAD4, SMAD3, PIK3R1, EGFR, TGFBR1, MSH6, MAPK9, NRAS, APC, CCND1, MYC, PMAIP1, GRB2, KRAS, RAF1, SOS1, [illegible]
KEGG_PATHWAYhsa05213:Endometrial cancer151.58562367864693449.364830183846266E-6GSK3B, PTEN, PIK3R1, FOXO3, EGFR, NRAS, APC, CCND1, CDH1, MYC, GRB2, KRAS, RAF1, SOS1, [illegible]
KEGG_PATHWAYhsa05214:Glioma171.7970401691331923 1.1365885100757654E-5RB1, SHC1, PTEN, PIK3R1, IGF1, EGFR, IGF1R, NRAS, CCND1, MDM2, E2F1, GRB2, KRAS, CALM3, RAF1, SOS1, [illegible]
KEGG_PATHWAYhsa05165:Human papillomavirus infection424.4397463002114171.3559898165705055E-5RB1, ITGB1, NOTCH2, GSK3B, NOTCH1, PTEN, PIK3R1, THBS2, THBS1, FOXO1, EGFR, LFNG, CCND3, NRAS, CCND1, PPP2R5E, E2F1, EP300, HES1, PRKACB, JAK1, FZD5, [illegible]
KEGG_PATHWAYhsa05218:Melanoma151.58562367864693441.1736293855390783E-4RB1, PTEN, PIK3R1, IGF1, EGFR, IGF1R, NRAS, CCND1, CDH1, MDM2, E2F1, KRAS, RAF1, [illegible]
KEGG_PATHWAYhsa05207:Chemical carcinogenesis - receptor activation272.8541226215644826.792334476712036E-4RB1, NOTCH2, SRC, GNAI3, XIAP, PIK3R1, CYP3A4, EGFR, [illegible]

Annotation Cluster 5Enrichment Score: 5.8409208190390585
CategoryTermCount%PValueGenesList TotalPop HitsPop TotalFold EnrichmentBonferroniBenjaminiFDR
REACTOME_PATHWAYR-HSA-2173793~Transcriptional activity of SMAD2/SMAD3:SMAD4 heterotrimer171.79704016913319232.696796878215734E-8TGIF1, SMAD2, CCNK, [illegible]
REACTOME_PATHWAYR-HSA-9006936~Signaling by TGFB family members262.7484143763213538.071575613015086E-8ITGB1, CCNK, CCNT2, BMPR2, CCNT1, UBE2D1, FSTL1, [illegible]
REACTOME_PATHWAYR-HSA-170834~Signaling by TGF-beta Receptor Complex222.3255813953488373 1.4167183966896169E-7ITGB1, TGIF1, SMAD2, CCNK, SMAD4, CCNT2, [illegible]
REACTOME_PATHWAYR-HSA-2173796~SMAD2/SMAD3:SMAD4 heterotrimer regulates transcription131.3742071881606766 7.217919770723371E-7TGIF1, SMAD2, CCNK, [illegible]
REACTOME_PATHWAYR-HSA-2173795~Downregulation of SMAD2/3:SMAD4 transcriptional activity101.0570824524312896 4.7112561938971116E-5SMAD2, TGIF1, PPM1A, [illegible]
KEGG_PATHWAYhsa04350:TGF-beta signaling pathway171.79704016913319238.586896418932655E-4TGIF1, SMAD2, SMAD4, BMPR2, SMAD3, CUL1, RGMB, THBS1, SMAD5, [illegible]

Annotation Cluster 6Enrichment Score: 5.446384930657536
CategoryTermCount%PValueGenesList TotalPop HitsPop TotalFold EnrichmentBonferroniBenjaminiFDR
REACTOME_PATHWAYR-HSA-449147~Signaling by Interleukins626.5539112050739961.2891086262785787E-8ITGB1, CXCL8, INPPL1, CXCL2, LMNB1, CRKL, RPS6KA5, CCND1, [illegible]
REACTOME_PATHWAYR-HSA-1280215~Cytokine Signaling in Immune system838.7737843551797036.087705862221435E-7ITGB1, EIF4A1, CXCL8, INPPL1, UBE2D1, ADAR, [illegible]
REACTOME_PATHWAYR-HSA-168256~Immune System15115.9619450317124740.005835821945909 6845EIF4A1, INPPL1, UBE2D1, CRKL, CDC20, NPEPPS, MT2A, HERC3, [illegible]

Annotation Cluster 7Enrichment Score: 5.067090510528449
CategoryTermCount%PValueGenesList TotalPop HitsPop TotalFold EnrichmentBonferroniBenjaminiFDR
KEGG_PATHWAYhsa05161:Hepatitis B343.59408033826638467.698464947038366E-10RB1, CXCL8, SRC, PIK3R1, MAPK9, NRAS, MYC, E2F1, PTK2B, EP300, JAK1, STAT5A, [illegible]
KEGG_PATHWAYhsa05160:Hepatitis C252.64270613107822433.538332290641505E-5RB1, GSK3B, SCARB1, PIK3R1, EGFR, NRAS, RXRA, CCND1, MYC, E2F1, LDLR, JAK1, CHUK, [illegible]
KEGG_PATHWAYhsa05162:Measles212.21987315010570853.023978736418619E-4STAT5A, GSK3B, STAT5B, CHUK, APAF1, STAT1, STAT3, TNFAIP3, ADAR, PIK3R1, MAPK9, [illegible]
KEGG_PATHWAYhsa05169:Epstein-Barr virus infection262.7484143763213536.544198626761708E-4RB1, PSMD14, TNFAIP3, PIK3R1, MAPK9, CCND3, CCND1, MYC, E2F1, HES1, [illegible]

Annotation Cluster 8Enrichment Score: 4.822068298531145
CategoryTermCount%PValueGenesList TotalPop HitsPop TotalFold EnrichmentBonferroniBenjaminiFDR
REACTOME_PATHWAYR-HSA-8939211~ESR-mediated signaling343.59408033826638461.408423905651618E-6FOXA1, DDX5, CCNT1, KDM1A, SHC1, SRC, GNAI3, GATA3, PIK3R1, [illegible]
REACTOME_PATHWAYR-HSA-9006931~Signaling by Nuclear Receptors414.3340380549682871.6149697192957574E-6FOXA1, DDX5, CCNT1, KDM1A, SHC1, SRC, GNAI3, GATA3, [illegible]
REACTOME_PATHWAYR-HSA-9009391~Extra-nuclear estrogen signaling181.9027484143763214 2.6051204677746397E-6HSP90AA1, SHC1, SRC, GNAI3, PIK3R1, GNG12, FOXO3, [illegible]
REACTOME_PATHWAYR-HSA-9018519~Estrogen-dependent gene expression181.90274841437632140.008689628767926338FOXA1, DDX5, HSP90AA1, CCNT1, KDM1A, GATA3, [illegible]

Annotation Cluster 9Enrichment Score: 3.8021486727224234
CategoryTermCount%PValueGenesList TotalPop HitsPop TotalFold EnrichmentBonferroniBenjaminiFDR
REACTOME_PATHWAYR-HSA-447115~Interleukin-12 family signaling141.4799154334038054 2.7073049034732258E-5STAT1, STAT3, SOD2, LMNB1, CNN2, RAP1B, MTAP, IFNG, [illegible]
REACTOME_PATHWAYR-HSA-8950505~Gene and protein expression by JAK-STAT signaling after Interleukin-12 stimulation101.0570824524312896 3.4690189612193 5E-4CNN2, [illegible]
REACTOME_PATHWAYR-HSA-9020591~Interleukin-12 signaling111.16279069767441874.176477425555969E-4CNN2, RAP1B, MTAP, IFNG, HNRNPF, TCP1, HNRNPA2B1, SOD2, [illegible]

Annotation Cluster 10Enrichment Score: 3.4402330394749403
CategoryTermCount%PValueGenesList TotalPop HitsPop TotalFold EnrichmentBonferroniBenjaminiFDR
KEGG_PATHWAYhsa01521:EGFR tyrosine kinase inhibitor resistance212.2198731501057085 4.551728348003391E-8GSK3B, SHC1, SRC, STAT3, PTEN, PIK3R1, IGF1, FOXO3, EGFR, [illegible]
REACTOME_PATHWAYR-HSA-5683057~MAPK family signaling cascades373.9112050739957716 3.160823047735688E-4RET, PSMD14, SHC1, SRC, PIK3R1, ABHD17C, FOXO3, [illegible]
REACTOME_PATHWAYR-HSA-5684996~MAPK1/MAPK3 signaling282.95983086680761080.01441547073737291 2RET, PSMD14, SHC1, SRC, PIK3R1, ABHD17C, EGFR, RAP1B, [illegible]
REACTOME_PATHWAYR-HSA-5673001~RAF/MAP kinase cascade242.53699788583509500.083610822328746 25RET, DUSP5, PSMD14, SHC1, SRC, PIK3R1, ABHD17C, EGFR, RAP1B, [illegible]

Annotation Cluster 11Enrichment Score: 3.4040588116331145
CategoryTermCount%PValueGenesList TotalPop HitsPop TotalFold EnrichmentBonferroniBenjaminiFDR
REACTOME_PATHWAYR-HSA-9679506~SARS-CoV Infections495.1797040169133199.890108489503807E-6RB1, ITGB1, GSK3B, KDM1A, RPS4X, GJA1, RPS18, EP300, IL6R, JAK1, [illegible]
REACTOME_PATHWAYR-HSA-9678108~SARS-CoV-1 Infection242.5369978858350951.3490077975424213E-5RB1, GSK3B, NPM1, DDX5, SMAD4, SMAD3, RPS5, YWHAZ, RUNX1, [illegible]
REACTOME_PATHWAYR-HSA-9692914~SARS-CoV-1-host interactions181.9027484143763214 5.614531346009656E-5NPM1, SMAD4, SMAD3, RPS5, YWHAZ, RUNX1, RPS4X, [illegible]
REACTOME_PATHWAYR-HSA-9694516~SARS-CoV-2 Infection313.27695560253699800.004344428643180914RB1, GSK3B, DDX5, RPN2, RPS4X, GJA1, GANAB, RPS15A, RPS18, [illegible]
REACTOME_PATHWAYR-HSA-9705683~SARS-CoV-2-host interactions232.43128964059196600.005739381976126924HSP90AA1, SEC24A, NUP133, CHUK, STAT1, RPS5, NUP153, [illegible]
REACTOME_PATHWAYR-HSA-9705671~SARS-CoV-2 activates/modulates innate and adaptive immune responses151.58562367864693440.020152099826278587HSP90AA1, [illegible]

Annotation Cluster 12Enrichment Score: 3.311721925860129
CategoryTermCount%PValueGenesList TotalPop HitsPop TotalFold EnrichmentBonferroniBenjaminiFDR
REACTOME_PATHWAYR-HSA-1059683~Interleukin-6 signaling70.7399577167019027 1.719262205793012E-5IL6, STAT1, STAT3, PTPN11, IL6ST, IL6R, JAK1673111109110.[illegible]
REACTOME_PATHWAYR-HSA-6783589~Interleukin-6 family signaling90.9513742071881607 5.374458738214156E-5IL6, STAT1, STAT3, LIF, PTPN11, LIFR, IL6ST, IL6R, [illegible]
REACTOME_PATHWAYR-HSA-112411~MAPK1 (ERK2) activation50.52854122621564480.001316725859199062 7IL6, PTPN11, IL6ST, IL6R, JAK16739110919.[illegible]
REACTOME_PATHWAYR-HSA-110056~MAPK3 (ERK1) activation50.52854122621564480.0020898663279894 22IL6, PTPN11, IL6ST, IL6R, JAK167310110918.[illegible]
REACTOME_PATHWAYR-HSA-112409~RAF-independent MAPK1/3 activation60.63424947145877380.010866749304413302DUSP5, IL6, PTPN11, IL6ST, IL6R, [illegible]

Annotation Cluster 13Enrichment Score: 3.2628729427209873
CategoryTermCount%PValueGenesList TotalPop HitsPop TotalFold EnrichmentBonferroniBenjaminiFDR
KEGG_PATHWAYhsa05163:Human cytomegalovirus infection404.22832980972515852.935647318837443E-9RB1, GSK3B, CXCL8, SRC, GNAI3, ITPR3, PIK3R1, EGFR, CX3CL1, [illegible]
KEGG_PATHWAYhsa04062:Chemokine signaling pathway343.59408033826638466.296422260658155E-8GSK3B, CXCL8, SHC1, SRC, GNAI3, PIK3R1, FOXO3, CXCL2, CX3CL1, [illegible]
REACTOME_PATHWAYR-HSA-372790~Signaling by GPCR434.5454545454545460.586057903178083 5ITGB1, CXCL8, SHC1, SRC, GNAI3, LPAR1, ITPR3, PIK3R1, CXCL2, EGFR, [illegible]
REACTOME_PATHWAYR-HSA-418594~G alpha (i) signalling events192.00845665961945 0.6531469116936222CXCL8, SRC, LPAR1, GNAI3, ITPR3, GNG12, CXCL2, CX3CL1, RGS5, [illegible]
REACTOME_PATHWAYR-HSA-388396~GPCR downstream signalling373.9112050739957716 0.6853085604019762ITGB1, CXCL8, SHC1, SRC, GNAI3, LPAR1, ITPR3, PIK3R1, CXCL2, [illegible]

Annotation Cluster 14Enrichment Score: 3.116749722416506
CategoryTermCount%PValueGenesList TotalPop HitsPop TotalFold EnrichmentBonferroniBenjaminiFDR
REACTOME_PATHWAYR-HSA-9006335~Signaling by Erythropoietin111.16279069767441878.876092703196064E-7STAT5A, STAT5B, NRAS, SHC1, RAPGEF1, GRB2, KRAS, PIK3R1, [illegible]
REACTOME_PATHWAYR-HSA-512988~Interleukin-3, Interleukin-5 and GM-CSF signaling131.37420718816067662.050757017187814E-5STAT5A, STAT5B, SHC1, INPPL1, PTPN11, [illegible]
REACTOME_PATHWAYR-HSA-9027284~Erythropoietin activates RAS70.73995771670190279.546181478707433E-5NRAS, SHC1, RAPGEF1, GRB2, KRAS, SOS1, CRKL67314110918[illegible]
REACTOME_PATHWAYR-HSA-8875555~MET activates RAP1 and RAC150.52854122621564480.003127770552924061RAP1B, RAPGEF1, GRB2, MET, CRKL67311110917.[illegible]
REACTOME_PATHWAYR-HSA-8875656~MET receptor recycling40.42283298097251587 0.019281221658774085GGA3, GRB2, MET, CRKL67310110916.591976225854383 0.[illegible]
REACTOME_PATHWAYR-HSA-8875878~MET promotes cell motility70.73995771670190270.035576827575425865RAP1B, ITGB1, SRC, RAPGEF1, GRB2, MET, CRKL67341110912.[illegible]
REACTOME_PATHWAYR-HSA-912631~Regulation of signaling by CBL50.52854122621564480.04085540953232024RAPGEF1, GRB2, PIK3R1, RPS27A, CRKL67322110913.[illegible]

Annotation Cluster 15Enrichment Score: 2.907104892107409
CategoryTermCount%PValueGenesList TotalPop HitsPop TotalFold EnrichmentBonferroniBenjaminiFDR
REACTOME_PATHWAYR-HSA-9006925~Intracellular signaling by second messengers373.9112050739957716 1.2415394626832412E-4PHLPP2, GSK3B, PSMD14, KDM1A, SRC, [illegible]
REACTOME_PATHWAYR-HSA-1257604~PIP3 activates AKT signaling333.48837209302325 61.749608099081863E-4PHLPP2, GSK3B, PSMD14, KDM1A, SRC, ATN1, PTEN, XIAP, [illegible]
REACTOME_PATHWAYR-HSA-6807070~PTEN Regulation181.90274841437632140.00468069114680524 9PHC2, PSMD14, KDM1A, PTEN, ATN1, XIAP, WWP2, BMI1, RPTOR, [illegible]
REACTOME_PATHWAYR-HSA-2219528~PI3K/AKT Signaling in Cancer131.37420718816067660.023140308125239345GSK3B, CHUK, SRC, PTEN, PTPN11, PIK3R1, FOXO3, FOXO1, [illegible]

Annotation Cluster 16Enrichment Score: 2.7527691843885176
CategoryTermCount%PValueGenesList TotalPop HitsPop TotalFold EnrichmentBonferroniBenjaminiFDR
REACTOME_PATHWAYR-HSA-9700206~Signaling by ALK in cancer161.6913319238900635 3.436558720931204E-4RB1, STAT5A, NPM1, SHC1, TPM3, STAT1, STAT3, CUL1, PIK3R1, [illegible]
REACTOME_PATHWAYR-HSA-9725370~Signaling by ALK fusions and activated point mutants161.6913319238900635 3.436558720931204E-4RB1, STAT5A, NPM1, SHC1, TPM3, [illegible]
REACTOME_PATHWAYR-HSA-9725371~Nuclear events stimulated by ALK signaling in cancer60.63424947145877380.0467137689683470 7RB1, STAT5A, NPM1, STAT3, CUL1, [illegible]

Annotation Cluster 17Enrichment Score: 2.62390151578515
CategoryTermCount%PValueGenesList TotalPop HitsPop TotalFold EnrichmentBonferroniBenjaminiFDR
REACTOME_PATHWAYR-HSA-9843745~Adipogenesis171.79704016913319238.357231366806488E-4SMAD4, HNRNPU, NR2F2, SLC2A4, MED4, MED13L, CCND3, MED14, RXRA, [illegible]
REACTOME_PATHWAYR-HSA-381340~Transcriptional regulation of white adipocyte differentiation141.47991543340380540.001531457624851599 2NR2F2, SLC2A4, MED4, [illegible]
REACTOME_PATHWAYR-HSA-1989781~PPARA activates gene expression161.69133192389006350.004613486494277280 5ABCA1, HMGCS1, FHL2, HMGCR, MED4, MED13L, [illegible]
REACTOME_PATHWAYR-HSA-400206~Regulation of lipid metabolism by PPARalpha161.69133192389006350.005410000100931693 4ABCA1, HMGCS1, FHL2, HMGCR, MED4, [illegible]

Annotation Cluster 18Enrichment Score: 2.5821786321356806
CategoryTermCount%PValueGenesList TotalPop HitsPop TotalFold EnrichmentBonferroniBenjaminiFDR
REACTOME_PATHWAYR-HSA-186763~Downstream signal transduction131.37420718816067664.435813429401424E-8STAT5A, STAT5B, STAT1, SRC, STAT3, PTPN11, PIK3R1, [illegible]
KEGG_PATHWAYhsa04917:Prolactin signaling pathway192.00845665961945 1.7120257510929604E-7STAT5A, GSK3B, STAT5B, SHC1, STAT1, SRC, STAT3, PIK3R1, FOXO3, [illegible]
REACTOME_PATHWAYR-HSA-9006335~Signaling by Erythropoietin111.16279069767441878.876092703196064E-7STAT5A, STAT5B, NRAS, SHC1, RAPGEF1, GRB2, KRAS, PIK3R1, [illegible]
REACTOME_PATHWAYR-HSA-9669938~Signaling by KIT in disease101.0570824524312896 9.574584476470295E-7STAT5A, STAT5B, NRAS, STAT1, SRC, STAT3, GRB2, KRAS, PIK3R1, [illegible]
REACTOME_PATHWAYR-HSA-9670439~Signaling by phosphorylated juxtamembrane, extracellular and kinase domain KIT mutants101.0570824524312896 9.574584476470295E-[illegible]
REACTOME_PATHWAYR-HSA-1236394~Signaling by ERBB4161.6913319238900635 1.204112240516704E-6YAP1, STAT5A, SHC1, SRC, PIK3R1, EGFR, ADAM17, ITCH, NRAS, [illegible]
KEGG_PATHWAYhsa05223:Non-small cell lung cancer181.9027484143763214 1.3824957988054916E-6RB1, STAT5A, RET, STAT5B, STAT3, PIK3R1, FOXO3, EGFR, NRAS, RXRA, [illegible]
REACTOME_PATHWAYR-HSA-9665348~Signaling by ERBB2 ECD mutants90.9513742071881607 1.452112958396732E-6HSP90AA1, NRAS, SHC1, ERBIN, GRB2, KRAS, PIK3R1, SOS1, [illegible]
REACTOME_PATHWAYR-HSA-9703648~Signaling by FLT3 ITD and TKD mutants90.9513742071881607 1.452112958396732E-6STAT5A, STAT5B, NRAS, GRB2, PTPN11, KRAS, [illegible]
REACTOME_PATHWAYR-HSA-9682385~FLT3 signaling in disease111.16279069767441873.017399754027774E-6STAT5A, STAT5B, NRAS, ZMYM2, GRB2, PTPN11, KRAS, PIK3R1, [illegible]
REACTOME_PATHWAYR-HSA-2428933~SHC-related events triggered by IGF1R70.73995771670190273.473176103338751E-6NRAS, SHC1, GRB2, KRAS, IGF1, SOS1, IGF1R6739110[illegible]
KEGG_PATHWAYhsa05211:Renal cell carcinoma171.79704016913319233.672274780136535 8E-6EGLN3, PTPN11, PIK3R1, ETS1, HIF1A, CRKL, RAP1B, NRAS, RAPGEF1, EP300, [illegible]
REACTOME_PATHWAYR-HSA-9674555~Signaling by CSF3 (G-CSF)111.16279069767441876.183937083619650 5E-6STAT5A, STAT5B, SHC1, STAT1, STAT3, UBE2D1, GRB2, PTPN11, [illegible]
REACTOME_PATHWAYR-HSA-186797~Signaling by PDGF151.58562367864693446.593286077130303 6E-6STAT5A, STAT5B, STAT1, SRC, STAT3, PTPN11, PIK3R1, THBS2, THBS1, [illegible]
REACTOME_PATHWAYR-HSA-1236382~Constitutive Signaling by Ligand-Responsive EGFR Cancer Variants90.9513742071881607 7.243826355762666E-6HSP90AA1, NRAS, SHC1, [illegible]
REACTOME_PATHWAYR-HSA-5637815~Signaling by Ligand-Responsive EGFR Variants in Cancer90.9513742071881607 7.243826355762666E-6HSP90AA1, NRAS, SHC1, GRB2, [illegible]
REACTOME_PATHWAYR-HSA-9680350~Signaling by CSF1 (M-CSF) in myeloid cells111.16279069767441878.637212400504945E-6SHC1, STAT1, SRC, STAT3, INPPL1, GRB2, [illegible]
REACTOME_PATHWAYR-HSA-9607240~FLT3 Signaling121.26849894291754759.927349422355088E-6STAT5A, STAT5B, NRAS, ABL2, GRB2, PTPN11, KRAS, PIK3R1, FOXO3, [illegible]
KEGG_PATHWAYhsa05214:Glioma171.7970401691331923 1.1365885100757654E-5RB1, SHC1, PTEN, PIK3R1, IGF1, EGFR, IGF1R, NRAS, CCND1, MDM2, E2F1, GRB2, KRAS, [illegible]
REACTOME_PATHWAYR-HSA-5637810~Constitutive Signaling by EGFRvIII80.8456659619450317 1.218263695534884 5E-5HSP90AA1, NRAS, SHC1, GRB2, KRAS, PIK3R1, SOS1, [illegible]
REACTOME_PATHWAYR-HSA-5637812~Signaling by EGFRvIII in Cancer80.8456659619450317 1.218263695534884 5E-5HSP90AA1, NRAS, SHC1, GRB2, KRAS, PIK3R1, SOS1, [illegible]
KEGG_PATHWAYhsa04935:Growth hormone synthesis, secretion and action222.3255813953488373 1.4513003287174237E-5STAT5A, MAP2K4, GSK3B, STAT5B, SHC1, STAT1, [illegible]
KEGG_PATHWAYhsa04012:ErbB signaling pathway181.9027484143763214 1.4739964753791436E-5STAT5A, MAP2K4, GSK3B, STAT5B, SHC1, SRC, PIK3R1, EGFR, CRKL, MAPK9, [illegible]
REACTOME_PATHWAYR-HSA-1059683~Interleukin-6 signaling70.7399577167019027 1.719262205793012E-5IL6, STAT1, STAT3, PTPN11, IL6ST, IL6R, JAK1673111109110.[illegible]
REACTOME_PATHWAYR-HSA-9634285~Constitutive Signaling by Overexpressed ERBB270.7399577167019027 1.719262205793012E-5HSP90AA1, NRAS, SHC1, ERBIN, GRB2, KRAS, [illegible]
REACTOME_PATHWAYR-HSA-512988~Interleukin-3, Interleukin-5 and GM-CSF signaling131.37420718816067662.050757017187814E-5STAT5A, STAT5B, SHC1, INPPL1, PTPN11, [illegible]
REACTOME_PATHWAYR-HSA-9673767~Signaling by PDGFRA transmembrane, juxtamembrane and kinase domain mutants70.73995771670190273.262399889424193 5E-5NRAS, [illegible]
REACTOME_PATHWAYR-HSA-9673770~Signaling by PDGFRA extracellular domain mutants70.73995771670190273.262399889424193 5E-5NRAS, STAT1, STAT3, GRB2, KRAS, [illegible]
REACTOME_PATHWAYR-HSA-1433557~Signaling by SCF-KIT121.26849894291754753.593368921477439E-5STAT5A, STAT5B, NRAS, STAT1, SRC, STAT3, GRB2, PTPN11, KRAS, [illegible]
KEGG_PATHWAYhsa05221:Acute myeloid leukemia151.58562367864693445.197617726359378E-5STAT5A, STAT5B, CHUK, STAT3, PIK3R1, RUNX1, CCNA2, NRAS, CCND1, MYC, [illegible]
REACTOME_PATHWAYR-HSA-6783589~Interleukin-6 family signaling90.9513742071881607 5.374458738214156E-5IL6, STAT1, STAT3, LIF, PTPN11, LIFR, IL6ST, IL6R, [illegible]
REACTOME_PATHWAYR-HSA-6806834~Signaling by MET161.6913319238900635 6.645310736838493E-5ITGB1, SHC1, SRC, STAT3, PTPN11, PIK3R1, CRKL, RAP1B, NRAS, GGA3, [illegible]
REACTOME_PATHWAYR-HSA-1643713~Signaling by EGFR in Cancer90.9513742071881607 7.487249632014922E-5HSP90AA1, NRAS, SHC1, GRB2, KRAS, PIK3R1, RPS27A, SOS1, [illegible]
REACTOME_PATHWAYR-HSA-9664565~Signaling by ERBB2 KD Mutants90.9513742071881607 7.487249632014922E-5HSP90AA1, NRAS, SHC1, ERBIN, GRB2, KRAS, PIK3R1, SOS1, [illegible]
REACTOME_PATHWAYR-HSA-9703465~Signaling by FLT3 fusion proteins80.8456659619450317 7.702093460483068E-5STAT5A, STAT5B, NRAS, ZMYM2, GRB2, KRAS, PIK3R1, [illegible]
REACTOME_PATHWAYR-HSA-8983432~Interleukin-15 signaling70.73995771670190279.546181478707433E-5STAT5A, STAT5B, SHC1, STAT3, GRB2, SOS1, JAK167314110918.[illegible]
REACTOME_PATHWAYR-HSA-9027284~Erythropoietin activates RAS70.73995771670190279.546181478707433E-5NRAS, SHC1, RAPGEF1, GRB2, KRAS, SOS1, CRKL67314110918[illegible]
REACTOME_PATHWAYR-HSA-1227990~Signaling by ERBB2 in Cancer90.9513742071881607 1.024560491422758 4E-4HSP90AA1, NRAS, SHC1, ERBIN, GRB2, KRAS, PIK3R1, SOS1, [illegible]
REACTOME_PATHWAYR-HSA-1227986~Signaling by ERBB2121.26849894291754751.5827183591346188E-4DIAPH1, HSP90AA1, NRAS, SHC1, SRC, ERBIN, GRB2, KRAS, PIK3R1, [illegible]
REACTOME_PATHWAYR-HSA-9665686~Signaling by ERBB2 TMD/JMD mutants80.8456659619450317 2.221576424129309E-4HSP90AA1, NRAS, SHC1, ERBIN, GRB2, KRAS, SOS1, [illegible]
REACTOME_PATHWAYR-HSA-5654736~Signaling by FGFR1121.26849894291754752.2911947850214656E-4TGFBR3, NRAS, SHC1, SRC, GIPC1, SPRY2, GRB2, PTPN11, KRAS, [illegible]
REACTOME_PATHWAYR-HSA-451927~Interleukin-2 family signaling111.16279069767441872.3595718326285264E-4STAT5A, STAT5B, SHC1, STAT1, STAT3, INPPL1, PTK2B, GRB2, [illegible]
REACTOME_PATHWAYR-HSA-8851805~MET activates RAS signaling60.63424947145877382.7353393441911246E-4NRAS, SHC1, GRB2, KRAS, SOS1, MET67311110918.[illegible]
REACTOME_PATHWAYR-HSA-177929~Signaling by EGFR121.26849894291754752.7357147797705725E-4ADAM17, NRAS, SHC1, SRC, SPRY2, GRB2, PTPN11, KRAS, PIK3R1, [illegible]
REACTOME_PATHWAYR-HSA-9034015~Signaling by NTRK3 (TRKC)70.73995771670190273.3625607077770515E-4NRAS, SHC1, SRC, GRB2, KRAS, PIK3R1, SOS167317110916.[illegible]
REACTOME_PATHWAYR-HSA-5655302~Signaling by FGFR1 in disease101.0570824524312896 3.4690189612193 5E-4STAT5A, STAT5B, NRAS, ZMYM2, STAT1, STAT3, GRB2, KRAS, [illegible]
KEGG_PATHWAYhsa04722:Neurotrophin signaling pathway192.00845665961945 3.5706045147442834E-4BEX3, GSK3B, SHC1, PTPN11, PIK3R1, FOXO3, MAPK14, CRKL, RAP1B, [illegible]
REACTOME_PATHWAYR-HSA-210993~Tie2 Signaling70.73995771670190274.787273166922458 6E-4NRAS, SHC1, GRB2, PTPN11, KRAS, PIK3R1, SOS167318110916.[illegible]
REACTOME_PATHWAYR-HSA-1839117~Signaling by cytosolic FGFR1 fusion mutants70.73995771670190274.787273166922458 6E-4STAT5A, STAT5B, ZMYM2, STAT1, STAT3, GRB2, [illegible]
REACTOME_PATHWAYR-HSA-2871796~FCERI mediated MAPK activation90.9513742071881607 4.991142964200937E-4VAV3, MAP2K4, MAPK9, NRAS, SHC1, GRB2, KRAS, SOS1, [illegible]
REACTOME_PATHWAYR-HSA-5654741~Signaling by FGFR3101.0570824524312896 5.21925057421673E-4NRAS, SHC1, SRC, SPRY2, GRB2, PTPN11, KRAS, PIK3R1, RPS27A, [illegible]
REACTOME_PATHWAYR-HSA-9006115~Signaling by NTRK2 (TRKB)80.8456659619450317 5.338794068977403E-4NRAS, SHC1, SRC, GRB2, PTPN11, KRAS, PIK3R1, [illegible]
REACTOME_PATHWAYR-HSA-187037~Signaling by NTRK1 (TRKA)181.9027484143763214 5.356079664559164E-4MEF2C, SHC1, SRC, STAT3, CLTA, PIK3R1, MAPK14, F3, CRKL, [illegible]
KEGG_PATHWAYhsa04625:C-type lectin receptor signaling pathway171.79704016913319236.265256990486126E-4CHUK, STAT1, SRC, RRAS2, PTPN11, ITPR3, PIK3R1, MAPK14, [illegible]
REACTOME_PATHWAYR-HSA-5654743~Signaling by FGFR4101.0570824524312896 6.333541681356125E-4NRAS, SHC1, SRC, SPRY2, GRB2, PTPN11, KRAS, PIK3R1, RPS27A, [illegible]
REACTOME_PATHWAYR-HSA-9034864~Activated NTRK3 signals through RAS50.52854122621564487.682371149105483E-4NRAS, SHC1, GRB2, KRAS, SOS167381109110.[illegible]
REACTOME_PATHWAYR-HSA-187687~Signalling to ERKs90.9513742071881607 7.737308830665493E-4NRAS, SHC1, SRC, RAPGEF1, GRB2, KRAS, MAPK14, SOS1, [illegible]
REACTOME_PATHWAYR-HSA-190236~Signaling by FGFR151.58562367864693448.688370766114459E-4SHC1, NCBP2, SRC, PTPN11, PIK3R1, TIAL1, TGFBR3, NRAS, GIPC1, [illegible]
REACTOME_PATHWAYR-HSA-167044~Signalling to RAS70.73995771670190279.006132695023671E-4NRAS, SHC1, SRC, GRB2, KRAS, MAPK14, SOS167320110915.[illegible]
REACTOME_PATHWAYR-HSA-9671555~Signaling by PDGFR in disease70.73995771670190279.006132695023671E-4NRAS, STAT1, STAT3, GRB2, KRAS, PIK3R1, SOS167320110915[illegible]
REACTOME_PATHWAYR-HSA-180336~SHC1 events in EGFR signaling60.63424947145877380.0010174842564112446NRAS, SHC1, GRB2, KRAS, SOS1, EGFR67314110917.[illegible]
REACTOME_PATHWAYR-HSA-166520~Signaling by NTRKs192.008456659619450.0011817183943875608MEF2C, SHC1, SRC, STAT3, CLTA, PTPN11, PIK3R1, MAPK14, F3, CRKL, [illegible]
REACTOME_PATHWAYR-HSA-2179392~EGFR Transactivation by Gastrin50.52854122621564480.0013167258591990627NRAS, GRB2, KRAS, SOS1, EGFR6739110919.[illegible]
REACTOME_PATHWAYR-HSA-8985947~Interleukin-9 signaling50.52854122621564480.0013167258591990627STAT5A, STAT5B, STAT1, STAT3, JAK16739110919.[illegible]
REACTOME_PATHWAYR-HSA-9026519~Activated NTRK2 signals through RAS50.52854122621564480.0013167258591990627NRAS, SHC1, GRB2, KRAS, SOS16739110919.[illegible]
REACTOME_PATHWAYR-HSA-5654738~Signaling by FGFR2131.37420718816067660.0013472529977468562SHC1, NCBP2, SRC, PTPN11, PIK3R1, TIAL1, NRAS, HNRNPF, SPRY2, [illegible]
KEGG_PATHWAYhsa04915:Estrogen signaling pathway192.008456659619450.0020579605273387674HSP90AA1, SHC1, SRC, GNAI3, ITPR3, PIK3R1, EGFR, HSP90B1, NRAS, [illegible]
REACTOME_PATHWAYR-HSA-9020958~Interleukin-21 signaling50.52854122621564480.0020898663279894 22STAT5A, STAT5B, STAT1, STAT3, JAK167310110918.[illegible]
REACTOME_PATHWAYR-HSA-9702518~STAT5 activation downstream of FLT3 ITD mutants50.52854122621564480.0020898663279894 22STAT5A, STAT5B, GRB2, PTPN11, [illegible]
REACTOME_PATHWAYR-HSA-8853659~RET signaling90.95137420718816070.002376447801957501 6RET, SHC1, ARTN, SRC, GRB2, PTPN11, PIK3R1, SOS1, PRKACB67340110913[illegible]
REACTOME_PATHWAYR-HSA-5655291~Signaling by FGFR4 in disease50.52854122621564480.003127770552924061NRAS, GRB2, KRAS, PIK3R1, SOS167311110917.[illegible]
REACTOME_PATHWAYR-HSA-9028731~Activated NTRK2 signals through FRS2 and FRS350.52854122621564480.003127770552924061NRAS, GRB2, PTPN11, KRAS, SOS1673111[illegible]
REACTOME_PATHWAYR-HSA-5654708~Downstream signaling of activated FGFR370.73995771670190270.003174855867957192NRAS, SHC1, GRB2, PTPN11, KRAS, PIK3R1, [illegible]
REACTOME_PATHWAYR-HSA-9705462~Inactivation of CSF3 (G-CSF) signaling70.73995771670190270.003174855867957192STAT5A, STAT5B, STAT1, STAT3, UBE2D1, RPS27A, [illegible]
KEGG_PATHWAYhsa05235:PD-L1 expression and PD-1 checkpoint pathway in cancer141.47991543340380540.0032102990240891 4CHUK, STAT1, STAT3, PTEN, PTPN11, PIK3R1, [illegible]
REACTOME_PATHWAYR-HSA-881907~Gastrin-CREB signalling pathway via PKC and MAPK60.63424947145877380.0035556385857211 78NRAS, CREB1, GRB2, KRAS, SOS1, [illegible]
KEGG_PATHWAYhsa04912:GnRH signaling pathway141.47991543340380540.004286851854277 7MAP2K4, SRC, ITPR3, MAPK14, EGFR, MAPK9, NRAS, PTK2B, GRB2, KRAS, [illegible]
REACTOME_PATHWAYR-HSA-1226099~Signaling by FGFR in disease111.16279069767441870.004305274039816318STAT5A, STAT5B, NRAS, ZMYM2, NCBP2, STAT1, STAT3, GRB2, [illegible]
REACTOME_PATHWAYR-HSA-9020558~Interleukin-2 signaling50.52854122621564480.0044688495995210 11STAT5A, STAT5B, SHC1, PTK2B, JAK167312110916.[illegible]
REACTOME_PATHWAYR-HSA-5654716~Downstream signaling of activated FGFR470.73995771670190270.004785873606934951 5NRAS, SHC1, GRB2, PTPN11, KRAS, PIK3R1, [illegible]
REACTOME_PATHWAYR-HSA-8876384~Listeria monocytogenes entry into host cells60.63424947145877380.0058193652737754354CDH1, SRC, CTNND1, GRB2, RPS27A, [illegible]
REACTOME_PATHWAYR-HSA-179812~GRB2 events in EGFR signaling50.52854122621564480.006149142520478656 5NRAS, GRB2, KRAS, SOS1, EGFR67313110916.[illegible]
REACTOME_PATHWAYR-HSA-112412~SOS-mediated signalling40.42283298097251587 0.0064473297467959 7NRAS, GRB2, KRAS, SOS16737110919.[illegible]
REACTOME_PATHWAYR-HSA-9645135~STAT5 Activation40.42283298097251587 0.0064473297467959 7STAT5A, STAT5B, GRB2, PTPN116737110919.[illegible]
REACTOME_PATHWAYR-HSA-1250347~SHC1 events in ERBB4 signaling50.52854122621564480.0082018058261435 1NRAS, SHC1, GRB2, KRAS, SOS167314110915.[illegible]
REACTOME_PATHWAYR-HSA-2424491~DAP12 signaling70.73995771670190270.0082268364846693 4VAV3, NRAS, SHC1, GRB2, KRAS, PIK3R1, SOS167330110913.[illegible]
REACTOME_PATHWAYR-HSA-5654696~Downstream signaling of activated FGFR270.73995771670190270.0082268364846693 4NRAS, SHC1, GRB2, PTPN11, KRAS, PIK3R1, [illegible]
REACTOME_PATHWAYR-HSA-1250196~SHC1 events in ERBB2 signaling60.63424947145877380.008940739783121772NRAS, SHC1, GRB2, KRAS, SOS1, EGFR67322110914.[illegible]
REACTOME_PATHWAYR-HSA-5654687~Downstream signaling of activated FGFR170.73995771670190270.009691026524495876NRAS, SHC1, GRB2, PTPN11, KRAS, PIK3R1, [illegible]
REACTOME_PATHWAYR-HSA-1839124~FGFR1 mutant receptor activation70.73995771670190270.009691026524495876STAT5A, STAT5B, ZMYM2, STAT1, STAT3, GRB2, [illegible]
REACTOME_PATHWAYR-HSA-1295596~Spry regulation of FGF signaling50.52854122621564480.013540335449876394SRC, SPRY2, GRB2, PTPN11, RPS27A67316110915.[illegible]
REACTOME_PATHWAYR-HSA-1963640~GRB2 events in ERBB2 signaling50.52854122621564480.013540335449876394NRAS, GRB2, KRAS, SOS1, EGFR67316110915.[illegible]
REACTOME_PATHWAYR-HSA-2428924~IGF1R signaling cascade90.95137420718816070.01373327395901062 3NRAS, SHC1, GRB2, PTPN11, KRAS, IGF1, PIK3R1, SOS1, [illegible]
REACTOME_PATHWAYR-HSA-2404192~Signaling by Type 1 Insulin-like Growth Factor 1 Receptor (IGF1R)90.95137420718816070.015293596314724094NRAS, SHC1, GRB2, [illegible]
REACTOME_PATHWAYR-HSA-8854691~Interleukin-20 family signaling60.63424947145877380.015528448909151915STAT5A, STAT5B, STAT1, STAT3, PTPN11, JAK1673251109[illegible]
REACTOME_PATHWAYR-HSA-180292~GAB1 signalosome50.52854122621564480.016875311517048 54SRC, GRB2, PTPN11, PIK3R1, EGFR67317110914.847041342539987 0.[illegible]
KEGG_PATHWAYhsa04660:T cell receptor signaling pathway151.58562367864693440.017215360323410 79VAV3, GSK3B, CHUK, PTPN11, PIK3R1, MAPK14, MAPK9, NRAS, IFNG, [illegible]
KEGG_PATHWAYhsa04072:Phospholipase D signaling pathway171.79704016913319230.020371140973173593CXCL8, SHC1, LPAR1, RRAS2, TSC1, PTPN11, PIK3R1, EGFR, [illegible]
REACTOME_PATHWAYR-HSA-5654704~SHC-mediated cascade:FGFR350.52854122621564480.020680646640305958NRAS, SHC1, GRB2, KRAS, SOS167318110914.[illegible]
REACTOME_PATHWAYR-HSA-912526~Interleukin receptor SHC signaling60.63424947145877380.021362178804103 81SHC1, INPPL1, GRB2, PIK3R1, SOS1, JAK167327110913.[illegible]
REACTOME_PATHWAYR-HSA-2219528~PI3K/AKT Signaling in Cancer131.37420718816067660.023140308125239345GSK3B, CHUK, SRC, PTEN, PTPN11, PIK3R1, FOXO3, FOXO1, [illegible]
KEGG_PATHWAYhsa04664:Fc epsilon RI signaling pathway101.0570824524312896 0.024520863027942215VAV3, MAP2K4, MAPK9, NRAS, GRB2, KRAS, PIK3R1, RAF1, MAPK14, [illegible]
KEGG_PATHWAYhsa04540:Gap junction121.26849894291754750.0248699304500182 1GJA1, NRAS, SRC, GNAI3, LPAR1, GRB2, KRAS, ITPR3, RAF1, SOS1, PRKACB, EGFR544928[illegible]
REACTOME_PATHWAYR-HSA-2586552~Signaling by Leptin40.42283298097251587 0.025338765823633494STAT5A, STAT5B, STAT3, PTPN1167311110915.992705659867622 1.[illegible]
REACTOME_PATHWAYR-HSA-9020956~Interleukin-27 signaling40.42283298097251587 0.025338765823633494STAT1, STAT3, IL6ST, JAK167311110915.992705659867622 1.0[illegible]
REACTOME_PATHWAYR-HSA-5654706~FRS-mediated FGFR3 signaling50.52854122621564480.029759623437987177NRAS, GRB2, PTPN11, KRAS, SOS167320110914.[illegible]
REACTOME_PATHWAYR-HSA-5654719~SHC-mediated cascade:FGFR450.52854122621564480.029759623437987177NRAS, SHC1, GRB2, KRAS, SOS167320110914.[illegible]
REACTOME_PATHWAYR-HSA-9013420~RHOU GTPase cycle70.73995771670190270.031944725564628504ITSN2, SRC, PTK2B, WWP2, GRB2, PIK3R1, PAK267340110912.[illegible]
REACTOME_PATHWAYR-HSA-9842663~Signaling by LTK40.42283298097251587 0.032295404565034186SHC1, GRB2, PIK3R1, SOS167312110915.4933135215453195 1.00.[illegible]
REACTOME_PATHWAYR-HSA-8984722~Interleukin-35 Signalling40.42283298097251587 0.032295404565034186STAT1, STAT3, IL6ST, JAK167312110915.4933135215453195 1[illegible]

REACTOME_PATHWAYR-HSA-8865999~MET activates PTPN1130.31712473572938690.032427014712059644GRB2, PTPN11, MET6735110919.8879643387815751.00.177716170797321470.158026900864785
REACTOME_PATHWAYR-HSA-5654688~SHC-mediated cascade:FGFR150.52854122621564480.03505274230523328NRAS, SHC1, GRB2, KRAS, SOS167321110913.9237953725323711.00.190427593777333150.169330018471362
REACTOME_PATHWAYR-HSA-2428928~IRS-related events triggered by IGF1R80.84566596194503170.03621165544695014NRAS, GRB2, PTPN11, KRAS, IGF1, PIK3R1, SOS1, IGF1R67352110912.5353754714824551.00.191325606370623740.17012853976753098
REACTOME_PATHWAYR-HSA-5654712~FRS-mediated FGFR4 signaling50.52854122621564480.04085540953232024NRAS, GRB2, PTPN11, KRAS, SOS167322110913.74544103741726351.00.202912500062204570.1804317152890117
REACTOME_PATHWAYR-HSA-5655332~Signaling by FGFR3 in disease50.52854122621564480.04085540953232024NRAS, GRB2, KRAS, PIK3R1, SOS167322110913.74544103741726351.00.202912500062204570.1804317152890117
REACTOME_PATHWAYR-HSA-8851907~MET activates PI3K/AKT signaling30.31712473572938690.04671197541775407GRB2, PIK3R1, MET6736110918.2399702823179781.00.220928515270841730.1964517265045396
REACTOME_PATHWAYR-HSA-2871809~FCERI mediated Ca+2 mobilization60.63424947145877380.04671376896834707VAV3, SHC1, GRB2, CALM3, ITPR3, SOS167333110912.99635282993381051.00.220928515270841730.1964517265045396
REACTOME_PATHWAYR-HSA-5654693~FRS-mediated FGFR1 signaling50.52854122621564480.04716881168506142NRAS, GRB2, PTPN11, KRAS, SOS167323110913.58259577492086031.00.220928515270841730.1964517265045396
REACTOME_PATHWAYR-HSA-5654699~SHC-mediated cascade:FGFR250.52854122621564480.04716881168506142NRAS, SHC1, GRB2, KRAS, SOS167323110913.58259577492086031.00.220928515270841730.1964517265045396
REACTOME_PATHWAYR-HSA-2172127~DAP12 interactions70.73995771670190270.05269466888126982VAV3, NRAS, SHC1, GRB2, KRAS, PIK3R1, SOS167345110912.5635463100544821.00.238063430365839660.2116882551608538
REACTOME_PATHWAYR-HSA-982772~Growth hormone receptor signaling50.52854122621564480.05399102796228333STAT5A, STAT5B, ADAM17, STAT1, STAT367324110913.43332095096582451.00.240530723544054050.2138821956038106
REACTOME_PATHWAYR-HSA-1170546~Prolactin receptor signaling40.422832980972515870.05839769524137099STAT5A, STAT5B, CUL1, PTPN1167315110914.3946508172362551.00.256210647729863130.2278249325745638
REACTOME_PATHWAYR-HSA-354194~GRB2:SOS provides linkage to MAPK signaling for Integrins 40.422832980972515870.05839769524137099RAP1B, SRC, GRB2, SOS167315110914.3946508172362551.00.256210647729863130.2278249325745638
REACTOME_PATHWAYR-HSA-5654700~FRS-mediated FGFR2 signaling50.52854122621564480.06131723349595063NRAS, GRB2, PTPN11, KRAS, SOS167325110913.29598811292719151.00.264335237931548340.23504939506781075
REACTOME_PATHWAYR-HSA-1266695~Interleukin-7 signaling60.63424947145877380.0640818383863155STAT5A, STAT5B, STAT3, PIK3R1, BRWD1, JAK167336110912.74665676077265971.00.270203956018211070.24026791472825348
REACTOME_PATHWAYR-HSA-2730905~Role of LAT2/NTAL/LAB on calcium mobilization40.422832980972515870.06874939365563379SHC1, GRB2, PIK3R1, SOS167316110914.1199851411589891.00.279168993992516050.24823971133437225
REACTOME_PATHWAYR-HSA-1433559~Regulation of KIT signaling40.422832980972515870.06874939365563379SRC, GRB2, SOCS6, SOS167316110914.1199851411589891.00.279168993992516050.24823971133437225
REACTOME_PATHWAYR-HSA-391160~Signal regulatory protein family interactions40.422832980972515870.06874939365563379SRC, PTK2B, GRB2, PTPN1167316110914.1199851411589891.00.279168993992516050.24823971133437225
REACTOME_PATHWAYR-HSA-983705~Signaling by the B Cell Receptor (BCR)121.26849894291754750.07653292377751417NRAS, PSMD14, PSMC3, CHUK, CUL1, GRB2, KRAS, CALM3, ITPR3, PIK3R1, RPS27A, SOS1673112110911.7657079176395671.00.3056026665683 [illegible]
REACTOME_PATHWAYR-HSA-354192~Integrin signaling50.52854122621564480.07744908492582908RAP1B, SHC1, SRC, GRB2, SOS167327110913.05184084530295461.00.30713790429396290.27310993105423936
KEGG_PATHWAYhsa05231:Choline metabolism in cancer111.16279069767441870.08182227410883602MAPK9, NRAS, SP1, TSC1, GRB2, KRAS, PIK3R1, RAF1, SOS1, HIF1A, EGFR5449988401.80555555555555560.99999999999863050.202335701090842560.137004 [illegible]
REACTOME_PATHWAYR-HSA-375165~NCAM signaling for neurite out-growth80.84566596194503170.08543373492088528NRAS, RPS6KA5, CREB1, ARTN, SRC, GRB2, KRAS, SOS167363110912.0926908653505981.00.32901480581754260.29256307891796496
REACTOME_PATHWAYR-HSA-5654732~Negative regulation of FGFR3 signaling50.52854122621564480.09547596552699736SRC, SPRY2, GRB2, PTPN11, RPS27A67329110912.84136906286826861.00.356594500556651760.31708720447651495
KEGG_PATHWAYhsa04662:B cell receptor signaling pathway101.05708245243128960.10550316176542117VAV3, GSK3B, NRAS, CHUK, INPPL1, GRB2, KRAS, PIK3R1, RAF1, SOS15449188401.78571428571428580.99999999999999970.24746697502330410.167563 [illegible]
REACTOME_PATHWAYR-HSA-74751~Insulin receptor signalling cascade70.73995771670190270.10676659430616071NRAS, SHC1, GRB2, PTPN11, KRAS, PIK3R1, SOS167354110912.1362885917120681.00.38228617341441430.33993248311114393
KEGG_PATHWAYhsa04217:Necroptosis151.58562367864693440.11015175279230233STAT5A, STAT5B, HSP90AA1, PARP1, STAT1, STAT3, XIAP, TNFAIP3, MAPK9, IFNG, CHMP2B, FAS, NLRP3, SLC25A5, JAK154415988401.53301886792452850.99999999999999990.25 [illegible]
REACTOME_PATHWAYR-HSA-5654733~Negative regulation of FGFR4 signaling50.52854122621564480.11527711227781075SRC, SPRY2, GRB2, PTPN11, RPS27A67331110912.6580549297799931.00.39931500304309430.35507467956688304
REACTOME_PATHWAYR-HSA-5655253~Signaling by FGFR2 in disease60.63424947145877380.11664535080095066NRAS, NCBP2, GRB2, KRAS, PIK3R1, SOS167343110912.299526590414321.00.39931500304309430.35507467956688304
REACTOME_PATHWAYR-HSA-74749~Signal attenuation30.31712473572938690.11946576925456481SHC1, GRB2, SOS167310110914.9439821693907881.00.40279022980186930.3581648840881019
REACTOME_PATHWAYR-HSA-388841~Costimulation by the CD28 family80.84566596194503170.12314489064174251PPP2R5E, SRC, BTLA, HLA-DRA, GRB2, PTPN11, PIK3R1, PAK267369110911.91071774662445871.00.413585456360736030.36776410170334345
REACTOME_PATHWAYR-HSA-983695~Antigen activates B Cell Receptor (BCR) leading to generation of second messengers50.52854122621564480.12579816867966498GRB2, CALM3, ITPR3, PIK3R1, SOS167332110912.5749907132243681.00.41844189313216780.372 [illegible]
REACTOME_PATHWAYR-HSA-202733~Cell surface interactions at the vascular wall131.37420718816067660.12666155853515762ITGB1, SLC16A1, SHC1, SRC, PTPN11, SLC7A11, PIK3R1, SLC7A5, NRAS, OLR1, GRB2, KRAS, SOS1673137110911.5637899805858941.00.41 [illegible]
REACTOME_PATHWAYR-HSA-5654726~Negative regulation of FGFR1 signaling50.52854122621564480.1367063617439312SRC, SPRY2, GRB2, PTPN11, RPS27A67333110912.49696069161150861.00.443655664610922760.39450281544456545
REACTOME_PATHWAYR-HSA-5654727~Negative regulation of FGFR2 signaling50.52854122621564480.14798052872760584SRC, SPRY2, GRB2, PTPN11, RPS27A67334110912.42352067126999371.00.46207253384674040.4108792698544876
REACTOME_PATHWAYR-HSA-112399~IRS-mediated signalling60.63424947145877380.16347782318872692NRAS, GRB2, PTPN11, KRAS, PIK3R1, SOS167348110912.05999257057949461.00.48761973766964500.43359608525615867
KEGG_PATHWAYhsa04650:Natural killer cell mediated cytotoxicity121.26849894291754750.17470779046825363VAV3, NRAS, IFNG, SHC1, FAS, PTK2B, GRB2, PTPN11, KRAS, PIK3R1, RAF1, SOS154413088401.50000000000000021.00.36665648131166390.248268 [illegible]
REACTOME_PATHWAYR-HSA-76009~Platelet Aggregation (Plug Formation)50.52854122621564480.20905380818988RAP1B, SHC1, SRC, GRB2, SOS167339110912.11281289290204551.00.56290476515425310.5005402441446648
REACTOME_PATHWAYR-HSA-199418~Negative regulation of the PI3K/AKT network101.05708245243128960.24563436822295032PHLPP2, PPP2R5E, SRC, PTEN, GRB2, PTPN11, PIK3R1, MET, EGFR, IER3673113110911.4584018198792881.00.60986298012947400.5422959332830465
REACTOME_PATHWAYR-HSA-1963642~PI3K events in ERBB2 signaling30.31712473572938690.25249314408876566GRB2, PIK3R1, EGFR67316110913.0899888558692421.00.61803759704213400.5495648800011128
REACTOME_PATHWAYR-HSA-8863795~Downregulation of ERBB2 signaling40.422832980972515870.25565850921996913HSP90AA1, ERBIN, RPS27A, EGFR67329110912.2730952502946151.00.61965901605343580.5510066611300314
REACTOME_PATHWAYR-HSA-182971~EGFR downregulation40.422832980972515870.2892126635230309SPRY2, GRB2, RPS27A, EGFR67331110912.12644394382399461.00.66919847421196090.5950576161342365
REACTOME_PATHWAYR-HSA-5654710~PI-3K cascade:FGFR330.31712473572938690.2984861943627842GRB2, PTPN11, PIK3R167318110912.74665676077265971.00.68152381400619890.60601742491838
KEGG_PATHWAYhsa04370:VEGF signaling pathway60.63424947145877380.30770857745310765NRAS, SRC, KRAS, PIK3R1, RAF1, MAPK145446088401.6251.00.56864811292401520.3850407285002736
REACTOME_PATHWAYR-HSA-389356~CD28 co-stimulation40.422832980972515870.3230587362190035SRC, GRB2, PIK3R1, PAK267333110911.9975685532892071.00.71291203849663340.6339281311732904
REACTOME_PATHWAYR-HSA-5654720~PI-3K cascade:FGFR430.31712473572938690.3440071139334081GRB2, PTPN11, PIK3R167320110912.4719910846953941.00.74989223703974370.6668112736746942
REACTOME_PATHWAYR-HSA-5654689~PI-3K cascade:FGFR130.31712473572938690.366421549995569GRB2, PTPN11, PIK3R167321110912.35427722351942271.00.76877547959118760.6836024316503291
REACTOME_PATHWAYR-HSA-389513~CTLA4 inhibitory signaling30.31712473572938690.366421549995569PPP2R5E, SRC, PTPN1167321110912.35427722351942271.00.76877547959118760.6836024316503291
REACTOME_PATHWAYR-HSA-74752~Signaling by Insulin receptor70.73995771670190270.37818991710213334NRAS, SHC1, GRB2, PTPN11, KRAS, PIK3R1, SOS167382110911.4068241945420941.00.78869208945607350.7013124696202016
REACTOME_PATHWAYR-HSA-5654695~PI-3K cascade:FGFR230.31712473572938690.4102937966504575GRB2, PTPN11, PIK3R167323110912.1495574649525161.00.81261617096599190.7225859893009772
REACTOME_PATHWAYR-HSA-6811558~PI5P, PP2A and IER3 Regulate PI3K/AKT Signaling80.84566596194503170.4635955811467577PPP2R5E, SRC, GRB2, PTPN11, PIK3R1, MET, EGFR, IER3673106110911.2437690992178081.00.88534258349471370.7872550035576191
REACTOME_PATHWAYR-HSA-2219530~Constitutive Signaling by Aberrant PI3K in Cancer60.63424947145877380.5148194105471553SRC, GRB2, PTPN11, PIK3R1, MET, EGFR67378110911.26768773574122751.00.93468149892951360.8311276340740799
KEGG_PATHWAYhsa04929:GnRH secretion50.52854122621564480.571961369359785NRAS, KRAS, ITPR3, PIK3R1, RAF15446588401.251.00.90773968570035530.614645053640366
REACTOME_PATHWAYR-HSA-109704~PI3K Cascade30.31712473572938690.7552850019199349GRB2, PTPN11, PIK3R167344110911.1236323112251791.01.00.8897228637413395

Annotation Cluster 19Enrichment Score: 2.3672366853790856
CategoryTermCount%PValueGenesList TotalPop HitsPop TotalFold EnrichmentBonferroniBenjaminiFDR
REACTOME_PATHWAYR-HSA-1266738~Developmental Biology12413.1078224101479936.819255220953402E-6FOXA1, EIF4A1, PLXND1, HNRNPU, CCND3, RPS6KA5, CCND1, RPS18, CDH1, MYC, DPF1, EP300, PKNOX1, PRKACB, IL6R, KRTAP13-2, MEF2C, SEMA6A, RPS5, SEMA6D, RPL22, HNF1B, [illegible]
REACTOME_PATHWAYR-HSA-9675108~Nervous system development485.073995771670190.019766674208059883RET, ITGB1, YAP1, GSK3B, SEMA7A, PLXND1, PSMD14, SHC1, SRC, SEMA3A, CLTA, WASL, HMGCR, PIK3R1, EGFR, RPS4X, NRAS, RPS15A, RPS6KA5, RPS18, ABL2, RPS27A, PAK2, MYH [illegible]
REACTOME_PATHWAYR-HSA-422475~Axon guidance464.8625792811839320.021769747428253758RET, ITGB1, GSK3B, SEMA7A, PLXND1, PSMD14, SHC1, SRC, SEMA3A, CLTA, WASL, PIK3R1, EGFR, RPS4X, NRAS, RPS15A, RPS6KA5, RPS18, ABL2, RPS27A, PAK2, MYH10, PRKACB, RPL19, VAV3, CA [illegible]
REACTOME_PATHWAYR-HSA-376176~Signaling by ROBO receptors192.008456659619450.11575221728325061CAP1, PSMD14, NCBP2, SRC, RPS5, RPL22, RPS4X, RPS15A, CXCL12, PSMC3, RPS18, ABL2, FAU, RPS27A, SOS1, PAK2, PRKACB, RPS24, RPL19673219110911.4297665330049461.00. [illegible]

Annotation Cluster 20Enrichment Score: 2.298653200659406
CategoryTermCount%PValueGenesList TotalPop HitsPop TotalFold EnrichmentBonferroniBenjaminiFDR
REACTOME_PATHWAYR-HSA-194138~Signaling by VEGF161.69133192389006350.002114723908613095VAV3, HSP90AA1, SRC, CTNND1, VEGFC, ITPR3, PIK3R1, MAPK14, CDH5, NRAS, ELMO1, PTK2B, KRAS, CALM3, PAK2, PRKACB673108110912.4414726762423640.97443625340647230.02 [illegible]
REACTOME_PATHWAYR-HSA-4420097~VEGFA-VEGFR2 Pathway151.58562367864693440.002475969229183245VAV3, HSP90AA1, SRC, CTNND1, ITPR3, PIK3R1, MAPK14, CDH5, NRAS, ELMO1, PTK2B, KRAS, CALM3, PAK2, PRKACB67399110912.49696069161150860.98634564050341140.03 [illegible]
KEGG_PATHWAYhsa04912:GnRH signaling pathway141.47991543340380540.00428685185427777MAP2K4, SRC, ITPR3, MAPK14, EGFR, MAPK9, NRAS, PTK2B, GRB2, KRAS, CALM3, RAF1, SOS1, PRKACB5449388402.44623655913978450.74709500117312190.016279830256126030.011023 [illegible]
REACTOME_PATHWAYR-HSA-5218920~VEGFR2 mediated vascular permeability60.63424947145877380.02846097695132277VAV3, CDH5, HSP90AA1, CTNND1, CALM3, PAK267329110913.40964287544192231.00.168913948824117670.15019988178763147

Annotation Cluster 21Enrichment Score: 2.258632634788041
CategoryTermCount%PValueGenesList TotalPop HitsPop TotalFold EnrichmentBonferroniBenjaminiFDR
REACTOME_PATHWAYR-HSA-5357801~Programmed Cell Death252.64270613107822430.002424323238592914PSMD14, HMGB2, XIAP, ADD1, LMNB1, CDH1, E2F1, PMAIP1, RPS27A, PAK2, HSP90AA1, PDCD6IP, APAF1, STAT3, YWHAZ, DYNLL2, ITCH, APC, PSMC3, IRF1, CHMP2B, NMT1, FAS, TP5 [illegible]
REACTOME_PATHWAYR-HSA-109606~Intrinsic Pathway for Apoptosis101.05708245243128960.00539504955117674APAF1, STAT3, NMT1, E2F1, PMAIP1, XIAP, TP53, DYNLL2, YWHAZ, BCL2L167355110912.99635282993381050.99991471595196960.058597063593216410.05210506347 [illegible]
REACTOME_PATHWAYR-HSA-109581~Apoptosis202.11416490486257920.012809026770474009PSMD14, APAF1, STAT3, HMGB2, XIAP, YWHAZ, DYNLL2, ADD1, LMNB1, APC, PSMC3, CDH1, NMT1, E2F1, FAS, PMAIP1, RPS27A, PAK2, TP53, BCL2L1673180110911.83110450718177290.99999 [illegible]

Annotation Cluster 22Enrichment Score: 2.1521943400264867
CategoryTermCount%PValueGenesList TotalPop HitsPop TotalFold EnrichmentBonferroniBenjaminiFDR
REACTOME_PATHWAYR-HSA-9824439~Bacterial Infection Pathways121.26849894291754750.004251961657923254MAP2K4, HSP90AA1, PDCD6IP, CDH1, SRC, MRC1, CTNND1, GRB2, CALM3, TRIM27, RPS27A, MET67373110912.70903132569358230.9993764606493 [illegible]
REACTOME_PATHWAYR-HSA-8876384~Listeria monocytogenes entry into host cells60.63424947145877380.0058193652737754354CDH1, SRC, CTNND1, GRB2, RPS27A, MET67320110914.9439821693907880.99995927148230380.0610519364543641650.0542879 [illegible]
REACTOME_PATHWAYR-HSA-8876493~InlA-mediated entry of Listeria monocytogenes into host cells40.422832980972515870.014123815059326587CDH1, SRC, CTNND1, RPS27A6739110917.3244180287270920.99999999998003110.10878476221250210.0967324 [illegible]

Annotation Cluster 23Enrichment Score: 2.0179874270978586
CategoryTermCount%PValueGenesList TotalPop HitsPop TotalFold EnrichmentBonferroniBenjaminiFDR
REACTOME_PATHWAYR-HSA-9843745~Adipogenesis171.79704016913319238.357231366806488E-4SMAD4, HNRNPU, NR2F2, SLC2A4, MED4, MED13L, CCND3, MED14, RXRA, MED13, MED30, CARM1, ZNF638, EP300, PPARG, PPARGC1A, PPARGC1B673109110912.5702 [illegible]
REACTOME_PATHWAYR-HSA-9843743~Transcriptional regulation of brown and beige adipocyte differentiation60.63424947145877380.03250780504486492SMAD4, RXRA, HNRNPU, PPARG, PPARGC1A, PPARGC1B67330110913.29598811292719151.00.17771617079 [illegible]
REACTOME_PATHWAYR-HSA-9844594~Transcriptional regulation of brown and beige adipocyte differentiation by EBF260.63424947145877380.03250780504486492SMAD4, RXRA, HNRNPU, PPARG, PPARGC1A, PPARGC1B67330110913.29598811292719151.00.17771 [illegible]

Annotation Cluster 24Enrichment Score: 1.90773690213608
CategoryTermCount%PValueGenesList TotalPop HitsPop TotalFold EnrichmentBonferroniBenjaminiFDR
REACTOME_PATHWAYR-HSA-9614085~FOXO-mediated transcription131.37420718816067664.597943792933953E-4SMAD2, SMAD4, TRIM63, SMAD3, FOXO3, SOD2, YWHAZ, SIRT1, FOXO1, PINK1, TXNIP, EP300, PPARGC1A67365110913.2959881129271920.54911682 [illegible]
REACTOME_PATHWAYR-HSA-9615017~FOXO-mediated transcription of oxidative stress, metabolic and neuronal genes80.84566596194503170.0014024704522850086SMAD2, SMAD4, TRIM63, SMAD3, FOXO3, SOD2, PPARGC1A, FOXO167329110914.546190500589230 [illegible]
REACTOME_PATHWAYR-HSA-3304349~Loss of Function of SMAD2/3 in Cancer40.422832980972515870.00644732974679597SMAD2, SMAD4, SMAD3, TGFBR16737110919.417108894077690.99998636579080180.06458510087397350.0574296828890901
REACTOME_PATHWAYR-HSA-2173789~TGF-beta receptor signaling activates SMADs90.95137420718816070.006693289586115743SMAD2, ITGB1, TGFBR3, PPP1CB, SMAD4, SMAD3, XPO1, RPS27A, TGFBR167347110913.15573329961114050.9999112035043250.06 [illegible]
REACTOME_PATHWAYR-HSA-3304351~Signaling by TGF-beta Receptor Complex in Cancer40.422832980972515870.009854785325946544SMAD2, SMAD4, SMAD3, TGFBR16738110918.2399702823179780.99999996447922110.085820818944047030.07631268435 [illegible]
REACTOME_PATHWAYR-HSA-3304347~Loss of Function of SMAD4 in Cancer30.31712473572938690.010554927888965228SMAD2, SMAD4, SMAD367331109116.4799405646359570.99999998956705690.090106847446190850.080123861462539
REACTOME_PATHWAYR-HSA-3311021~SMAD4 MH2 Domain Mutants in Cancer30.31712473572938690.010554927888965228SMAD2, SMAD4, SMAD367331109116.4799405646359570.99999998956705690.090106847446190850.080123861462539
REACTOME_PATHWAYR-HSA-3315487~SMAD2/3 MH2 Domain Mutants in Cancer30.31712473572938690.010554927888965228SMAD2, SMAD4, SMAD367331109116.4799405646359570.99999998956705690.090106847446190850.080123861462539
REACTOME_PATHWAYR-HSA-9617828~FOXO-mediated transcription of cell cycle genes50.52854122621564480.01687531151704854SMAD2, SMAD4, SMAD3, FOXO3, FOXO167317110914.8470413425399870.99999999999984220.120846755615888920.10745807 [illegible]
REACTOME_PATHWAYR-HSA-1502540~Signaling by Activin50.52854122621564480.01687531151704854SMAD2, TGFBR3, SMAD4, SMAD3, ACVR2B67317110914.8470413425399870.99999999999984220.120846755615888920.10745807870980083
REACTOME_PATHWAYR-HSA-8952158~RUNX3 regulates BCL2L11 (BIM) transcription30.31712473572938690.032427014712059644SMAD4, SMAD3, FOXO36735110919.8879643387815751.00.177716170797321470.158026900864785
REACTOME_PATHWAYR-HSA-1181150~Signaling by NODAL50.52854122621564480.04085540953232024SMAD2, SMAD4, SMAD3, FOXO3, ACVR2B67322110913.74544103741726351.00.202912500062204570.1804317152890117
REACTOME_PATHWAYR-HSA-9823730~Formation of definitive endoderm40.422832980972515870.05839769524137099SMAD2, SMAD4, SMAD3, CDH167315110914.3946508172362551.00.256210647729863130.2278249325745638
REACTOME_PATHWAYR-HSA-9754189~Germ layer formation at gastrulation30.31712473572938690.2755101941849841SMAD2, SMAD4, SMAD367317110912.90822480552399251.00.64696364027449530.5752861913808408

Annotation Cluster 25Enrichment Score: 1.8440903400472952
CategoryTermCount%PValueGenesList TotalPop HitsPop TotalFold EnrichmentBonferroniBenjaminiFDR
REACTOME_PATHWAYR-HSA-9759194~Nuclear events mediated by NFE2L2151.58562367864693440.002036885809800527GSK3B, ABCC1, NOTCH1, PRKAA2, PSMD14, CUL1, SLC7A11, PSMC3, SP1, MYC, PRDX1, EP300, RPS27A, NFE2L2, BCL2L167397110912.5484444 [illegible]
REACTOME_PATHWAYR-HSA-9755511~KEAP1-NFE2L2 pathway161.69133192389006350.00911253871552004GSK3B, ABCC1, NOTCH1, PRKAA2, PSMD14, CUL1, SLC7A11, PSMC3, SP1, MYC, PRDX1, SESN2, EP300, RPS27A, NFE2L2, BCL2L1673126110912.09269086535 [illegible]
REACTOME_PATHWAYR-HSA-9711123~Cellular response to chemical stress232.43128964059196660.011022550428121756GSK3B, ABCC1, NOTCH1, PRKAA2, PSMD14, STAT3, CUL1, SLC7A11, SOD2, PRDX3, RXRA, PSMC3, SP1, MYC, CARM1, PRDX1, SESN2, TXNIP, EP300, N [illegible]
REACTOME_PATHWAYR-HSA-9762114~GSK3B and BTRC:CUL1-mediated-degradation of NFE2L260.63424947145877380.20547097242933288GSK3B, PSMD14, PSMC3, CUL1, RPS27A, NFE2L267352110911.9015316036118411.00.56236306495676360.500058559202 [illegible]

Annotation Cluster 26Enrichment Score: 1.7485126673991962
CategoryTermCount%PValueGenesList TotalPop HitsPop TotalFold EnrichmentBonferroniBenjaminiFDR
REACTOME_PATHWAYR-HSA-6804116~TP53 Regulates Transcription of Genes Involved in G1 Cell Cycle Arrest50.52854122621564480.00820180582614351CCNA2, CDK2, E2F1, TP53, E2F767314110915.8856930587985570.99999936147415620.07583567887197855 [illegible]
REACTOME_PATHWAYR-HSA-1538133~G0 and Early G160.63424947145877380.02136217880410381CCNA2, LIN54, MYC, CDK2, E2F1, E2F467327110913.66220901436354570.99999999999999990.140762950066585180.12516774728944474
REACTOME_PATHWAYR-HSA-68911~G2 Phase30.31712473572938690.032427014712059644CCNA2, CDK2, E2F16735110919.8879643387815751.00.177716170797321470.158026900864785

Annotation Cluster 27Enrichment Score: 1.7106536357665707
CategoryTermCount%PValueGenesList TotalPop HitsPop TotalFold EnrichmentBonferroniBenjaminiFDR
REACTOME_PATHWAYR-HSA-194315~Signaling by Rho GTPases596.2367864693446090.010421739717781948ITSN2, ITGB1, DOCK4, PLXND1, KDM1A, WASL, LMNB1, SYDE2, CDC20, PPP1CB, PREX1, GJA1, XPO1, PPP2R5E, CDH1, EFHD2, LBR, VAV3, HSP90AA1, NUP133, T [illegible]
REACTOME_PATHWAYR-HSA-9716542~Signaling by Rho GTPases, Miro GTPases and RHOBTB3606.3424947145877380.010667101258752991ITSN2, ITGB1, DOCK4, PLXND1, KDM1A, WASL, LMNB1, SYDE2, CDC20, PPP1CB, PREX1, GJA1, XPO1, PPP2R5E, CDH1, EFHD2, LBR, [illegible]
REACTOME_PATHWAYR-HSA-9012999~RHO GTPase cycle404.22832980972515850.014276474493000253ITGB1, ITSN2, DOCK4, PLXND1, SRC, TWF1, WASL, PIK3R1, ADD3, TOR1AIP1, LMNB1, SYDE2, PREX1, GJA1, EFHD2, PTK2B, ABL2, CCT7, ECT2, PAK2, LBR, VAV3, HSP90 [illegible]
REACTOME_PATHWAYR-HSA-195258~RHO GTPase Effectors272.8541226215644820.09052694050949503ITGB1, KDM1A, SRC, CDCA8, WASL, CDC20, PPP1CB, XPO1, PPP2R5E, CDH1, FLNA, BUB3, PAK2, MYH10, NUP133, RANGAP1, MAPK14, YWHAZ, DYNLL2, RHOB, DIAPH [illegible]

Annotation Cluster 28Enrichment Score: 1.7103030031202433
CategoryTermCount%PValueGenesList TotalPop HitsPop TotalFold EnrichmentBonferroniBenjaminiFDR
REACTOME_PATHWAYR-HSA-3304349~Loss of Function of SMAD2/3 in Cancer40.422832980972515870.00644732974679597SMAD2, SMAD4, SMAD3, TGFBR16737110919.417108894077690.99998636579080180.06458510087397350.0574296828890901
REACTOME_PATHWAYR-HSA-2173789~TGF-beta receptor signaling activates SMADs90.95137420718816070.006693289586115743SMAD2, ITGB1, TGFBR3, PPP1CB, SMAD4, SMAD3, XPO1, RPS27A, TGFBR167347110913.15573329961114050.9999112035043250.06 [illegible]
REACTOME_PATHWAYR-HSA-3304351~Signaling by TGF-beta Receptor Complex in Cancer40.422832980972515870.009854785325946544SMAD2, SMAD4, SMAD3, TGFBR16738110918.2399702823179780.99999996447922110.085820818944047030.07631268435 [illegible]
REACTOME_PATHWAYR-HSA-2173788~Downregulation of TGF-beta receptor signaling60.63424947145877380.018292492656275222SMAD2, PPP1CB, SMAD3, XPO1, RPS27A, TGFBR167326110913.8030632072236820.99999999999999870.127826168440826450.11 [illegible]
REACTOME_PATHWAYR-HSA-3656532~TGFBR1 KD Mutants in Cancer30.31712473572938690.04671197541775407SMAD2, SMAD3, TGFBR16736110918.2399702823179781.00.220928515270841730.1964517265045396
REACTOME_PATHWAYR-HSA-3304356~SMAD2/3 Phosphorylation Motif Mutants in Cancer30.31712473572938690.04671197541775407SMAD2, SMAD3, TGFBR16736110918.2399702823179781.00.220928515270841730.1964517265045396
REACTOME_PATHWAYR-HSA-3656534~Loss of Function of TGFBR1 in Cancer30.31712473572938690.06281783368539047SMAD2, SMAD3, TGFBR16737110917.0628316705582681.00.26629274399064090.23679002798013715

Annotation Cluster 29Enrichment Score: 1.6939986091772554
CategoryTermCount%PValueGenesList TotalPop HitsPop TotalFold EnrichmentBonferroniBenjaminiFDR
REACTOME_PATHWAYR-HSA-76002~Platelet activation, signaling and aggregation323.3826638477801272.7671426591773147E-4SHC1, SRC, GNAI3, ITPR3, PIK3R1, THBS1, PIK3CG, RAP1B, GNA12, LAMP2, PSAP, FLNA, VAV3, CAP1, ABCC4, VEGFC, PTPN11, ACTN4, IGF1, M [illegible]
REACTOME_PATHWAYR-HSA-114608~Platelet degranulation 121.26849894291754750.15849930926759723CAP1, ABCC4, LAMP2, PSAP, VEGFC, FLNA, CALM3, ACTN4, IGF1, ALDOA, HRG, THBS1673129110911.53301772694287971.00.483175527269396670.4296442 [illegible]
REACTOME_PATHWAYR-HSA-76005~Response to elevated platelet cytosolic Ca2+121.26849894291754750.18877533468356977CAP1, ABCC4, LAMP2, PSAP, VEGFC, FLNA, CALM3, ACTN4, IGF1, ALDOA, HRG, THBS1673134110911.47581557295247381.00.5354298772 [illegible]

Annotation Cluster 30Enrichment Score: 1.525823733978363
CategoryTermCount%PValueGenesList TotalPop HitsPop TotalFold EnrichmentBonferroniBenjaminiFDR
REACTOME_PATHWAYR-HSA-1059683~Interleukin-6 signaling70.73995771670190271.719262205793012E-5IL6, STAT1, STAT3, PTPN11, IL6ST, IL6R, JAK1673111109110.4872349047683380.0293388846098722186.771548642362021E-46.021325134379618E-4
REACTOME_PATHWAYR-HSA-8854691~Interleukin-20 family signaling60.63424947145877380.015528448909151915STAT5A, STAT5B, STAT1, STAT3, PTPN11, JAK167325110913.95518573551262960.9999999999998310.114514050891745820.10182697774043874
REACTOME_PATHWAYR-HSA-877312~Regulation of IFNG signaling40.422832980972515870.04884950427375973IFNG, STAT1, PTPN11, JAK167314110914.7085544470388451.00.226353451621458870.20127563124562498
REACTOME_PATHWAYR-HSA-877300~Interferon gamma signaling90.95137420718816070.22540874047730358MT2A, VCAM1, IFNG, STAT1, IRF1, HLA-DRA, PTPN11, RAF1, JAK167396110911.5449944279346211.00.58153089001276790.5171027706345501
REACTOME_PATHWAYR-HSA-912694~Regulation of IFNA/IFNB signaling30.31712473572938690.47312394076060205STAT1, PTPN11, JAK167326110911.9015316036118411.00.89511330713768920.795943223484812
REACTOME_PATHWAYR-HSA-909733~Interferon alpha/beta signaling60.63424947145877380.503247428821425STAT1, IRF1, PTPN11, ADAR, RPS27A, JAK167377110911.2841512128287761.00.92227777430455950.8200981247566798

Annotation Cluster 31Enrichment Score: 1.4764156448864538
CategoryTermCount%PValueGenesList TotalPop HitsPop TotalFold EnrichmentBonferroniBenjaminiFDR
REACTOME_PATHWAYR-HSA-9012852~Signaling by NOTCH3101.05708245243128960.002408247224532984NEURL1B, NOTCH1, PLXND1, STAT1, FABP7, EP300, HES1, WWP2, RPS27A, EGFR67349110913.36325317645631830.98464193862917650.03256862149210 [illegible]
REACTOME_PATHWAYR-HSA-1980143~Signaling by NOTCH1111.16279069767441870.01335197119418976NEURL1B, ADAM17, ITCH, NOTCH1, FBXW7, MYC, CUL1, EP300, HES1, RPS27A, HIF1A67374110912.4497208947431830.99999999999222550.10614204623638 [illegible]
REACTOME_PATHWAYR-HSA-9013508~NOTCH3 Intracellular Domain Regulates Transcription60.63424947145877380.015528448909151915NOTCH1, PLXND1, STAT1, FABP7, EP300, HES167325110913.95518573551262960.9999999999998310.11451405089174582 [illegible]
REACTOME_PATHWAYR-HSA-2894862~Constitutive Signaling by NOTCH1 HD+PEST Domain Mutants90.95137420718816070.022829688362931286NEURL1B, ADAM17, NOTCH1, FBXW7, MYC, CUL1, EP300, HES1, RPS27A67358110912.5572321565814421.00.1433472 [illegible]
REACTOME_PATHWAYR-HSA-2644602~Signaling by NOTCH1 PEST Domain Mutants in Cancer90.95137420718816070.022829688362931286NEURL1B, ADAM17, NOTCH1, FBXW7, MYC, CUL1, EP300, HES1, RPS27A67358110912.5572321565814421.00.1433472823657 [illegible]
REACTOME_PATHWAYR-HSA-2644603~Signaling by NOTCH1 in Cancer90.95137420718816070.022829688362931286NEURL1B, ADAM17, NOTCH1, FBXW7, MYC, CUL1, EP300, HES1, RPS27A67358110912.5572321565814421.00.14334728236579680.127465760026 [illegible]
REACTOME_PATHWAYR-HSA-2644606~Constitutive Signaling by NOTCH1 PEST Domain Mutants90.95137420718816070.022829688362931286NEURL1B, ADAM17, NOTCH1, FBXW7, MYC, CUL1, EP300, HES1, RPS27A67358110912.5572321565814421.00.14334728236 [illegible]
REACTOME_PATHWAYR-HSA-2894858~Signaling by NOTCH1 HD+PEST Domain Mutants in Cancer90.95137420718816070.022829688362931286NEURL1B, ADAM17, NOTCH1, FBXW7, MYC, CUL1, EP300, HES1, RPS27A67358110912.5572321565814421.00.1433472823 [illegible]
REACTOME_PATHWAYR-HSA-2122947~NOTCH1 Intracellular Domain Regulates Transcription80.84566596194503170.02449415180266912NOTCH1, FBXW7, MYC, CUL1, EP300, HES1, RPS27A, HIF1A67348110912.74665676077265971.00.152691960697933750.1357 [illegible]
REACTOME_PATHWAYR-HSA-9013694~Signaling by NOTCH4111.16279069767441870.02588147751997873NOTCH2, NOTCH1, SMAD3, PSMD14, PSMC3, FBXW7, CUL1, EP300, HES1, RPS27A, YWHAZ67382110912.21072373428043361.00.156827274622808180.1394 [illegible]
REACTOME_PATHWAYR-HSA-9013695~NOTCH4 Intracellular Domain Regulates Transcription50.52854122621564480.029759623437987177NOTCH2, NOTCH1, SMAD3, EP300, HES167320110914.1199851411589891.00.17423455208794520.15493101256060215
REACTOME_PATHWAYR-HSA-2197563~NOTCH2 intracellular domain regulates transcription40.422832980972515870.032295404565034186NOTCH2, CREB1, EP300, HES167312110915.49331352154531951.00.177716170797321470.158026900864785
REACTOME_PATHWAYR-HSA-2644605~FBXW7 Mutants and NOTCH1 in Cancer30.31712473572938690.032427014712059644NOTCH1, FBXW7, CUL16735110919.8879643387815751.00.177716170797321470.158026900864785
REACTOME_PATHWAYR-HSA-2644607~Loss of Function of FBXW7 in Cancer and NOTCH1 Signaling30.31712473572938690.032427014712059644NOTCH1, FBXW7, CUL16735110919.8879643387815751.00.177716170797321470.158026900864785
REACTOME_PATHWAYR-HSA-186712~Regulation of beta-cell development70.73995771670190270.035576827575425865NOTCH1, NR5A2, EP300, HNF1B, HES1, PAX6, FOXO167341110912.8136483890841881.00.19088124516474620.16973340957811536
REACTOME_PATHWAYR-HSA-1980145~Signaling by NOTCH260.63424947145877380.04671376896834707NOTCH2, NEURL1B, CREB1, EP300, HES1, RPS27A67333110912.99635282993381051.00.220928515270841730.1964517265045396
REACTOME_PATHWAYR-HSA-2691230~Signaling by NOTCH1 HD Domain Mutants in Cancer40.422832980972515870.05839769524137099NEURL1B, ADAM17, NOTCH1, RPS27A67315110914.3946508172362551.00.256210647729863130.2278249325745638
REACTOME_PATHWAYR-HSA-2691232~Constitutive Signaling by NOTCH1 HD Domain Mutants40.422832980972515870.05839769524137099NEURL1B, ADAM17, NOTCH1, RPS27A67315110914.3946508172362551.00.256210647729863130.2278249325745638
REACTOME_PATHWAYR-HSA-210744~Regulation of gene expression in late stage (branching morphogenesis) pancreatic bud precursor cells40.422832980972515870.06874939365563379NOTCH1, EP300, HNF1B, HES167316110914.1199851411589891.00.2791689939 [illegible]
REACTOME_PATHWAYR-HSA-2122948~Activated NOTCH1 Transmits Signal to the Nucleus50.52854122621564480.11527711227781075NEURL1B, ADAM17, ITCH, NOTCH1, RPS27A67331110912.6580549297799931.00.39931500304309430.35507467956688304
KEGG_PATHWAYhsa04330:Notch signaling pathway70.73995771670190270.17929766473558822LFNG, NOTCH2, ADAM17, ITCH, NOTCH1, EP300, HES15446288401.83467741935483851.00.37382977157289310.2531261149208304
REACTOME_PATHWAYR-HSA-8941856~RUNX3 regulates NOTCH signaling30.31712473572938690.2067081779442922NOTCH1, EP300, HES167314110913.5314158352791341.00.56236306495676360.500058559202754

Annotation Cluster 32Enrichment Score: 1.4409309431700688
CategoryTermCount%PValueGenesList TotalPop HitsPop TotalFold EnrichmentBonferroniBenjaminiFDR
REACTOME_PATHWAYR-HSA-5218859~Regulated Necrosis90.95137420718816070.027441019559481503HSP90AA1, ITCH, PDCD6IP, IRF1, CHMP2B, FAS, XIAP, RPS27A, TP5367360110912.4719910846953941.00.163983747919246360.14581590048676207
REACTOME_PATHWAYR-HSA-5675482~Regulation of necroptotic cell death60.63424947145877380.041629717480539105HSP90AA1, ITCH, PDCD6IP, FAS, XIAP, RPS27A67332110913.0899888558692421.00.204374788650918630.18173199614025712
REACTOME_PATHWAYR-HSA-5213460~RIPK1-mediated regulated necrosis60.63424947145877380.041629717480539105HSP90AA1, ITCH, PDCD6IP, FAS, XIAP, RPS27A67332110913.0899888558692421.00.204374788650918630.18173199614025712

Annotation Cluster 33Enrichment Score: 1.4326633655438994
CategoryTermCount%PValueGenesList TotalPop HitsPop TotalFold EnrichmentBonferroniBenjaminiFDR
REACTOME_PATHWAYR-HSA-9694686~Replication of the SARS-CoV-2 genome30.31712473572938690.02026395216255958RB1, DDX5, VHL67341109112.3599554234769680.99999999999999960.135588529334809830.12056660340735256
REACTOME_PATHWAYR-HSA-9679514~SARS-CoV-1 Genome Replication and Transcription30.31712473572938690.02026395216255958RB1, DDX5, VHL67341109112.3599554234769680.99999999999999960.135588529334809830.12056660340735256
REACTOME_PATHWAYR-HSA-9682706~Replication of the SARS-CoV-1 genome30.31712473572938690.02026395216255958RB1, DDX5, VHL67341109112.3599554234769680.99999999999999960.135588529334809830.12056660340735256
REACTOME_PATHWAYR-HSA-9694682~SARS-CoV-2 Genome Replication and Transcription30.31712473572938690.02026395216255958RB1, DDX5, VHL67341109112.3599554234769680.99999999999999960.135588529334809830.12056660340735256
REACTOME_PATHWAYR-HSA-9772572~Early SARS-CoV-2 Infection Events40.422832980972515870.40718012009777904RB1, DDX5, CHMP2B, VHL67338110911.73473058575115351.00.81181397908818490.721872672691802

Annotation Cluster 34Enrichment Score: 1.3570715533698605
CategoryTermCount%PValueGenesList TotalPop HitsPop TotalFold EnrichmentBonferroniBenjaminiFDR
REACTOME_PATHWAYR-HSA-3214841~PKMTs methylate histone lysines101.05708245243128960.022765431805228047ATF7IP, KMT2D, KMT2A, RBBP5, NSD1, EHMT2, KMT2C, SETD7, KMT5B, EZH267369110912.38839718328057331.00.14334728236579680.127469 [illegible]
REACTOME_PATHWAYR-HSA-4839726~Chromatin organization242.5369978858350950.0610597081044575ATF7IP, SMARCD1, KMT2D, KDM3B, KMT2A, KDM1A, TRRAP, EHMT2, KMT2C, SETD7, PAX3, SUPT7L, WDR77, HCFC1, CCND1, KANSL1, RBBP5, CARM1, NSD1, HAT1 [illegible]
REACTOME_PATHWAYR-HSA-3247509~Chromatin modifying enzymes242.5369978858350950.0610597081044575ATF7IP, SMARCD1, KMT2D, KDM3B, KMT2A, KDM1A, TRRAP, EHMT2, KMT2C, SETD7, PAX3, SUPT7L, WDR77, HCFC1, CCND1, KANSL1, RBBP5, CARM1, NSD1 [illegible]

Annotation Cluster 35Enrichment Score: 1.3503634188644058
CategoryTermCount%PValueGenesList TotalPop HitsPop TotalFold EnrichmentBonferroniBenjaminiFDR
REACTOME_PATHWAYR-HSA-381119~Unfolded Protein Response (UPR)131.37420718816067660.011947834495289064KLHDC3, CXCL8, SHC1, ASNS, EIF2S2, PDIA6, PDIA5, ADD1, HSP90B1, SERP1, KHSRP, SSR1, DCP267395110912.25514976147649950.99999999990 [illegible]
REACTOME_PATHWAYR-HSA-381038~XBP1(S) activates chaperone genes70.73995771670190270.08008735694992845KLHDC3, SERP1, SHC1, SSR1, PDIA6, ADD1, PDIA567350110912.3071916790490341.00.315434976350513650.28048776604509035
REACTOME_PATHWAYR-HSA-381070~IRE1alpha activates chaperones70.73995771670190270.09290874294772208KLHDC3, SERP1, SHC1, SSR1, PDIA6, ADD1, PDIA567352110912.2184535375471481.00.35002359027913560.311244288874869

Annotation Cluster 36Enrichment Score: 1.2985922572080648
CategoryTermCount%PValueGenesList TotalPop HitsPop TotalFold EnrichmentBonferroniBenjaminiFDR
KEGG_PATHWAYhsa04110:Cell cycle262.7484143763213531.060552348226789E-5RB1, GSK3B, CUL1, PDS5B, CDC20, CCNB2, CCND3, CCND1, PPP2R5E, CHEK2, MYC, CDC27, E2F1, EP300, E2F4, BUB3, SMAD2, SMAD4, SMAD3, YWHAZ, CCNA2, WEE1, CDK2, MDM2, ATM, [illegible]
REACTOME_PATHWAYR-HSA-1640170~Cell Cycle565.91966173361522150.022009268977078715RB1, LIN54, GSK3B, UBE2D1, FOXM1, LMNB1, CDC20, PPP1CB, CCND3, XPO1, CCND1, PPP2R5E, MYC, CHEK2, CDC27, EP300, TOPBP1, TNPO1, LBR, SUN2, HSP90AA1, NUP1 [illegible]
REACTOME_PATHWAYR-HSA-68882~Mitotic Anaphase232.4312896405919660.027868529315751265NUP133, PSMD14, CDCA8, UBE2D1, PDS5B, RANGAP1, LEMD2, DYNLL2, LMNB1, NUP93, CDC20, CCNB2, XPO1, PSMC3, PPP2R5E, TAOK1, CDC27, CHMP2B, CENPM, BU [illegible]
REACTOME_PATHWAYR-HSA-2555396~Mitotic Metaphase and Anaphase232.4312896405919660.029054672305666572NUP133, PSMD14, CDCA8, UBE2D1, PDS5B, RANGAP1, LEMD2, DYNLL2, LMNB1, NUP93, CDC20, CCNB2, XPO1, PSMC3, PPP2R5E, TAOK1, CDC27, CH [illegible]
REACTOME_PATHWAYR-HSA-69278~Cell Cycle, Mitotic464.8625792811839320.03120504586707762RB1, GSK3B, LIN54, PSMD14, SRC, CUL1, UBE2D1, CDCA8, PDS5B, FOXM1, LMNB1, CDC20, PPP1CB, CCNB2, CCND3, XPO1, CCND1, PPP2R5E, MYC, CDC27, E2F1, EP300, [illegible]
REACTOME_PATHWAYR-HSA-69618~Mitotic Spindle Checkpoint121.26849894291754750.07653292377751417CDC20, XPO1, NUP133, PPP2R5E, TAOK1, CDC27, CDCA8, UBE2D1, CENPM, BUB3, RANGAP1, DYNLL2673112110911.7657079176395671.00.30560266568 [illegible]
REACTOME_PATHWAYR-HSA-69620~Cell Cycle Checkpoints252.64270613107822430.07695665848622088PSMD14, UBE2D1, CDCA8, CDC20, CCNB2, XPO1, PPP2R5E, CHEK2, CDC27, TOPBP1, BUB3, RPS27A, NUP133, RANGAP1, YWHAZ, DYNLL2, CCNA2, WEE1, PSMC3, TA [illegible]
REACTOME_PATHWAYR-HSA-5663220~RHO GTPases Activate Formins141.47991543340380540.08196001564382077ITGB1, NUP133, SRC, CDCA8, RANGAP1, DYNLL2, RHOB, CDC20, DIAPH1, XPO1, PPP2R5E, TAOK1, CENPM, BUB3673140110911.64799405646359581.0 [illegible]
REACTOME_PATHWAYR-HSA-195258~RHO GTPase Effectors272.8541226215644820.09052694050949503ITGB1, KDM1A, SRC, CDCA8, WASL, CDC20, PPP1CB, XPO1, PPP2R5E, CDH1, FLNA, BUB3, PAK2, MYH10, NUP133, RANGAP1, MAPK14, YWHAZ, DYNLL2, RHOB, DIAPH [illegible]
REACTOME_PATHWAYR-HSA-141424~Amplification of signal from the kinetochores101.05708245243128960.12680283947514132CDC20, XPO1, NUP133, PPP2R5E, TAOK1, CDCA8, CENPM, BUB3, RANGAP1, DYNLL267396110911.71666047548291221.00.4185701348 [illegible]
REACTOME_PATHWAYR-HSA-141444~Amplification of signal from unattached kinetochores via a MAD2 inhibitory signal101.05708245243128960.12680283947514132CDC20, XPO1, NUP133, PPP2R5E, TAOK1, CDCA8, CENPM, BUB3, RANGAP1, DYNLL267396110911.7 [illegible]
REACTOME_PATHWAYR-HSA-2500257~Resolution of Sister Chromatid Cohesion121.26849894291754750.14712520209582775CDC20, CCNB2, XPO1, NUP133, PPP2R5E, TAOK1, CDCA8, CENPM, BUB3, PDS5B, RANGAP1, DYNLL2673127110911.55715973839079941.00 [illegible]
REACTOME_PATHWAYR-HSA-9648025~EML4 and NUDC in mitotic spindle formation111.16279069767441870.17124085346405102CDC20, XPO1, NUP133, PPP2R5E, TAOK1, NEK6, CDCA8, CENPM, BUB3, RANGAP1, DYNLL2673117110911.54939612146150061.00.502 [illegible]
REACTOME_PATHWAYR-HSA-2467813~Separation of Sister Chromatids161.69133192389006350.17740320204427462NUP133, PSMD14, CDCA8, UBE2D1, PDS5B, RANGAP1, DYNLL2, CDC20, XPO1, PSMC3, PPP2R5E, TAOK1, CDC27, CENPM, BUB3, RPS27A673190110911 [illegible]
REACTOME_PATHWAYR-HSA-68886~M Phase303.1712473572938690.24974739018076442RB1, PSMD14, UBE2D1, CDCA8, PDS5B, LMNB1, CDC20, CCNB2, XPO1, PPP2R5E, CDC27, CEP192, BUB3, TNPO1, RPS27A, LBR, HSP90AA1, NUP133, NEK6, NUP153, RANGAP1, LEM [illegible]
REACTOME_PATHWAYR-HSA-68877~Mitotic Prometaphase151.58562367864693440.3608627796523233HSP90AA1, NUP133, NEK6, CDCA8, PDS5B, RANGAP1, DYNLL2, CDC20, CCNB2, XPO1, PPP2R5E, TAOK1, CENPM, CEP192, BUB3673205110911.2058493096075094 [illegible]

Annotation Cluster 37Enrichment Score: 1.2712734437526103
CategoryTermCount%PValueGenesList TotalPop HitsPop TotalFold EnrichmentBonferroniBenjaminiFDR
REACTOME_PATHWAYR-HSA-9861718~Regulation of pyruvate metabolism70.73995771670190270.013159029150246932MKLN1, PDHX, LDHA, PKM, RMND5A, RPS27A, PDK167333110913.4957449682561120.9999999998913320.105090311139990480.093447299 [illegible]
REACTOME_PATHWAYR-HSA-70268~Pyruvate metabolism80.84566596194503170.027137631320747327MKLN1, PDHX, LDHB, LDHA, PKM, RMND5A, RPS27A, PDK167349110912.69060254116505471.00.16329692735713580.14520517314330428
REACTOME_PATHWAYR-HSA-1428517~Aerobic respiration and respiratory electron transport151.58562367864693440.4299162705842142PDHX, MDH2, SDHD, ACAT1, MKLN1, LDHB, LDHA, PKM, RMND5A, NDUFS6, UQCRFS1, UQCRC2, NDUFV3, RPS27A, PDK167321511 [illegible]

Annotation Cluster 38Enrichment Score: 1.263522357862267
CategoryTermCount%PValueGenesList TotalPop HitsPop TotalFold EnrichmentBonferroniBenjaminiFDR
REACTOME_PATHWAYR-HSA-450294~MAP kinase activation111.16279069767441870.004833051700368525MAP2K4, MAPK9, MEF2C, RPS6KA5, CREB1, CHUK, CUL1, TAB3, TAB2, MAPK14, RPS27A67364110912.83248978454680560.9997731313456950.0540366361 [illegible]
REACTOME_PATHWAYR-HSA-168643~Nucleotide-binding domain, leucine rich repeat containing receptor (NLR) signaling pathways101.05708245243128960.006856224908512204ITCH, CHUK, TXNIP, NLRP3, TAB3, TNFAIP3, TAB2, MAPK14, RPS27A, BCL2L167357110912 [illegible]
REACTOME_PATHWAYR-HSA-75893~TNF signaling101.05708245243128960.006856224908512204ADAM17, CHUK, SPPL2A, RACK1, TAB3, TNFAIP3, UBE2D1, XIAP, TAB2, RPS27A67357110912.89121764291858870.99999331655742810.066751897564335110.05935 [illegible]
REACTOME_PATHWAYR-HSA-448424~Interleukin-17 signaling111.16279069767441870.011099719459537163MAP2K4, MAPK9, MEF2C, RPS6KA5, CREB1, CHUK, CUL1, TAB3, TAB2, MAPK14, RPS27A67372110912.5177686973749380.99999999598088750.09247987 [illegible]
REACTOME_PATHWAYR-HSA-5357956~TNFR1-induced NF-kappa-B signaling pathway70.73995771670190270.013159029150246932CHUK, RACK1, TAB3, TNFAIP3, XIAP, TAB2, RPS27A67333110913.4957449682561120.9999999998913320.105090311139990480.09 [illegible]
REACTOME_PATHWAYR-HSA-937061~TRIF (TICAM1)-mediated TLR4 signaling 141.47991543340380540.016561781811928626MAP2K4, MEF2C, CHUK, CUL1, UBE2D1, PTPN11, MAPK14, MAPK9, RPS6KA5, CREB1, TAB3, TAB2, RPS27A, TP53673111110912.07855106220 [illegible]
REACTOME_PATHWAYR-HSA-166166~MyD88-independent TLR4 cascade 141.47991543340380540.016561781811928626MAP2K4, MEF2C, CHUK, CUL1, UBE2D1, PTPN11, MAPK14, MAPK9, RPS6KA5, CREB1, TAB3, TAB2, RPS27A, TP53673111110912.078551062206337 [illegible]
REACTOME_PATHWAYR-HSA-168638~NOD1/2 Signaling Pathway70.73995771670190270.022526167426888264ITCH, CHUK, TAB3, TNFAIP3, TAB2, MAPK14, RPS27A67337110913.11782659330950551.00.14334728236579680.12746576002636634
REACTOME_PATHWAYR-HSA-168164~Toll Like Receptor 3 (TLR3) Cascade131.37420718816067660.026430199572557177MAP2K4, MEF2C, CHUK, CUL1, UBE2D1, MAPK14, MAPK9, RPS6KA5, CREB1, TAB3, TAB2, RPS27A, TP53673106110912.02112478622893831.00.1 [illegible]
REACTOME_PATHWAYR-HSA-168142~Toll Like Receptor 10 (TLR10) Cascade121.26849894291754750.032334593342597494MAP2K4, MAPK9, MEF2C, RPS6KA5, CREB1, CHUK, CUL1, TAB3, TAB2, MAPK14, RPS27A, TP5367397110912.03875553376939681.00.17771617 [illegible]
REACTOME_PATHWAYR-HSA-168176~Toll Like Receptor 5 (TLR5) Cascade121.26849894291754750.032334593342597494MAP2K4, MAPK9, MEF2C, RPS6KA5, CREB1, CHUK, CUL1, TAB3, TAB2, MAPK14, RPS27A, TP5367397110912.03875553376939681.00.1777161707 [illegible]
REACTOME_PATHWAYR-HSA-975871~MyD88 cascade initiated on plasma membrane121.26849894291754750.032334593342597494MAP2K4, MAPK9, MEF2C, RPS6KA5, CREB1, CHUK, CUL1, TAB3, TAB2, MAPK14, RPS27A, TP5367397110912.03875553376939681.00.1 [illegible]
REACTOME_PATHWAYR-HSA-166058~MyD88:MAL(TIRAP) cascade initiated on plasma membrane131.37420718816067660.04063286957901051MAP2K4, MEF2C, CHUK, CUL1, MAPK14, MAPK9, RPS6KA5, CREB1, TAB3, TAB2, TLR6, RPS27A, TP53673113110911.8959223 [illegible]
REACTOME_PATHWAYR-HSA-168188~Toll Like Receptor TLR6:TLR2 Cascade131.37420718816067660.04063286957901051MAP2K4, MEF2C, CHUK, CUL1, MAPK14, MAPK9, RPS6KA5, CREB1, TAB3, TAB2, TLR6, RPS27A, TP53673113110911.89592236584307481.00.20 [illegible]
REACTOME_PATHWAYR-HSA-73887~Death Receptor Signaling161.69133192389006350.046457039627108454VAV3, BEX3, CHUK, SPPL2A, XIAP, TNFAIP3, UBE2D1, PREX1, ADAM17, RACK1, FAS, TAB3, TAB2, ECT2, RPS27A, SOS1673154110911.71220161710503451.00. [illegible]
REACTOME_PATHWAYR-HSA-975138~TRAF6 mediated induction of NFkB and MAP kinases upon TLR7/8 or 9 activation121.26849894291754750.046994959602086356MAP2K4, MAPK9, MEF2C, RPS6KA5, CREB1, CHUK, CUL1, TAB3, TAB2, MAPK14, RPS27A, TP536731031 [illegible]
REACTOME_PATHWAYR-HSA-450321~JNK (c-Jun kinases) phosphorylation and activation mediated by activated human TAK150.52854122621564480.04716881168506142MAP2K4, MAPK9, TAB3, TAB2, RPS27A67323110913.58259577492086031.00.22092851527084 [illegible]
REACTOME_PATHWAYR-HSA-181438~Toll Like Receptor 2 (TLR2) Cascade131.37420718816067660.04810759248267288MAP2K4, MEF2C, CHUK, CUL1, MAPK14, MAPK9, RPS6KA5, CREB1, TAB3, TAB2, TLR6, RPS27A, TP53673116110911.84688989086437471.00.22411 [illegible]
REACTOME_PATHWAYR-HSA-168179~Toll Like Receptor TLR1:TLR2 Cascade131.37420718816067660.04810759248267288MAP2K4, MEF2C, CHUK, CUL1, MAPK14, MAPK9, RPS6KA5, CREB1, TAB3, TAB2, TLR6, RPS27A, TP53673116110911.84688989086437471.00.224 [illegible]
REACTOME_PATHWAYR-HSA-975155~MyD88 dependent cascade initiated on endosome121.26849894291754750.04981647751950806MAP2K4, MAPK9, MEF2C, RPS6KA5, CREB1, CHUK, CUL1, TAB3, TAB2, MAPK14, RPS27A, TP53673104110911.9015316036118411.00 [illegible]
REACTOME_PATHWAYR-HSA-168898~Toll-like Receptor Cascades171.79704016913319230.05049307726990489MAP2K4, MEF2C, CHUK, CUL1, UBE2D1, PTPN11, MAPK14, HSP90B1, MAPK9, RPS6KA5, CREB1, TAB3, TAB2, TLR6, RPS27A, TP53, CTSB673169110911.6577 [illegible]
REACTOME_PATHWAYR-HSA-168181~Toll Like Receptor 7/8 (TLR7/8) Cascade121.26849894291754750.05275035040997255MAP2K4, MAPK9, MEF2C, RPS6KA5, CREB1, CHUK, CUL1, TAB3, TAB2, MAPK14, RPS27A, TP53673105110911.8834217788155381.00.2380634 [illegible]

REACTOME_PATHWAYR-HSA-166016~Toll Like Receptor 4 (TLR4) Cascade151.58562367864693440.06184477073403267MAP2K4, MEF2C, CHUK, CUL1, UBE2D1, PTPN11, MAPK14, MAPK9, RPS6KA5, CREB1, TAB3, TAB2, TLR6, RPS27A, TP53673147110911.6816265882281591.00.26594786025329680.23648335409713234
REACTOME_PATHWAYR-HSA-168138~Toll Like Receptor 9 (TLR9) Cascade121.26849894291754750.062239184091861244MAP2K4, MAPK9, MEF2C, RPS6KA5, CREB1, CHUK, CUL1, TAB3, TAB2, MAPK14, RPS27A, TP53673108110911.83110450718177291.00.26629274399064090.23679002798013715
REACTOME_PATHWAYR-HSA-5357905~Regulation of TNFR1 signaling70.73995771670190270.06832590002942002CHUK, SPPL2A, RACK1, TNFAIP3, UBE2D1, XIAP, RPS27A67348110912.4033246656760771.00.279168993992516050.24823971133437225
REACTOME_PATHWAYR-HSA-5684264~MAP3K8 (TPL2)-dependent MAPK1/3 activation40.422832980972515870.06874939365563379MAP2K4, CHUK, CUL1, RPS27A67316110914.1199851411589891.00.279168993992516050.24823971133437225
REACTOME_PATHWAYR-HSA-450282~MAPK targets/ Nuclear events mediated by MAP kinases50.52854122621564480.11527711227781075MAPK9, MEF2C, RPS6KA5, CREB1, MAPK1467331110912.6580549297799931.00.39931500304309430.35507467956688304
REACTOME_PATHWAYR-HSA-937042~IRAK2 mediated activation of TAK1 complex30.31712473572938690.11946576925456481TAB3, TAB2, RPS27A67310110914.9439821693907881.00.40279022980186930.3581648840881019
REACTOME_PATHWAYR-HSA-9645460~Alpha-protein kinase 1 signaling pathway30.31712473572938690.1403828811282177TAB3, TAB2, RPS27A67311110914.4945292449007161.00.44886260700221640.3991328778940655
REACTOME_PATHWAYR-HSA-9014325~TICAM1,TRAF6-dependent induction of TAK1 complex30.31712473572938690.1403828811282177TAB3, TAB2, RPS27A67311110914.4945292449007161.00.44886260700221640.3991328778940655
REACTOME_PATHWAYR-HSA-450302~activated TAK1 mediates p38 MAPK activation40.422832980972515870.17518552220053485TAB3, TAB2, MAPK14, RPS27A67324110912.74665676077265971.00.50768647152763690.4514396149013783
REACTOME_PATHWAYR-HSA-446652~Interleukin-1 family signaling131.37420718816067660.21702238975670668MAP2K4, SMAD3, PSMD14, CHUK, STAT3, CUL1, PTPN11, PTPN14, PSMC3, TAB3, TAB2, RPS27A, TP53673153110911.40025638784488551.00.57778048477195090.5137678748029869
REACTOME_PATHWAYR-HSA-2871837~FCERI mediated NF-kB activation80.84566596194503170.22779891522475615PSMD14, PSMC3, CHUK, CUL1, TAB3, UBE2D1, TAB2, RPS27A67382110911.60779907947667881.00.58153089001276790.5171027706345501
REACTOME_PATHWAYR-HSA-975163~IRAK2 mediated activation of TAK1 complex upon TLR7/8 or 9 stimulation30.31712473572938690.25249314408876566TAB3, TAB2, RPS27A67316110913.0899888558692421.00.6180375970421340.5495648800011128
REACTOME_PATHWAYR-HSA-937072~TRAF6-mediated induction of TAK1 complex within TLR4 complex30.31712473572938690.2755101941849841TAB3, TAB2, RPS27A67317110912.90822480552399251.00.64696364027449530.5752861913808408
REACTOME_PATHWAYR-HSA-445989~TAK1-dependent IKK and NF-kappa-B activation 50.52854122621564480.28961272686611117CHUK, TAB3, TAB2, RPS27A, TP5367345110911.83110450718177291.00.66919847421196090.5950576161342365
REACTOME_PATHWAYR-HSA-9758274~Regulation of NF-kappa B signaling30.31712473572938690.2984861943627842CHUK, RPS27A, TP5367318110912.74665676077265971.00.68152381400619890.60601742491838
REACTOME_PATHWAYR-HSA-9020702~Interleukin-1 signaling90.95137420718816070.39621342756033895MAP2K4, PSMD14, PSMC3, CHUK, CUL1, TAB3, TAB2, RPS27A, TP53673115110911.28973447897150971.00.79564063726777490.7074911840909412

Annotation Cluster 39Enrichment Score: 1.2391108276784373
CategoryTermCount%PValueGenesList TotalPop HitsPop TotalFold EnrichmentBonferroniBenjaminiFDR
REACTOME_PATHWAYR-HSA-9009391~Extra-nuclear estrogen signaling181.90274841437632142.6051204677746397E-6HSP90AA1, SHC1, SRC, GNAI3, PIK3R1, GNG12, FOXO3, EGFR, IGF1R, NRAS, CREB1, XPO1, CCND1, GNB2, GNB4, KRAS, CALM3, GNB567377110913.8524536384863280.0045019104120236351.7364129887128657E-41.5440348[illegible]
KEGG_PATHWAYhsa04926:Relaxin signaling pathway232.43128964059196612.061471785647616E-5SMAD2, MAP2K4, SHC1, SRC, GNAI3, VEGFC, PIK3R1, GNG12, MAPK14, EGFR, TGFBR1, MAPK9, NRAS, MMP13, CREB1, GNB2, GNB4, GRB2, KRAS, GNB5, RAF1, SOS1, PRKACB54413088402.8750.0038522552023059219.619023749053974E-56.5131[illegible]
KEGG_PATHWAYhsa04371:Apelin signaling pathway222.32558139534883731.1829845256659792E-4SMAD2, MEF2C, SMAD4, SMAD3, PRKAA2, GNAI3, RRAS2, ITPR3, GNG12, TGFBR1, PIK3CG, NRAS, CCND1, CDH1, GNB2, GNB4, KRAS, CALM3, GNB5, RAF1, PPARGC1A, PRKACB54414088402.5535714285714290.037150097792238587.2571550709[illegible]
REACTOME_PATHWAYR-HSA-9824443~Parasitic Infection Pathways242.5369978858350951.3675716345624484E-4VAV3, SRC, FZD7, PLK2, GNAI3, ITPR3, WASL, GNG12, MAPK14, MYH2, IL6, ADAM17, MYO1C, CREB1, ADORA2B, GNB2, GNB4, ELMO1, TXNIP, NLRP3, GRB2, CALM3, GNB5, PRKACB673164110912.41169861921501830.2109137156317[illegible]
REACTOME_PATHWAYR-HSA-9658195~Leishmania infection242.5369978858350951.3675716345624484E-4VAV3, SRC, FZD7, PLK2, GNAI3, ITPR3, WASL, GNG12, MAPK14, MYH2, IL6, ADAM17, MYO1C, CREB1, ADORA2B, GNB2, GNB4, ELMO1, TXNIP, NLRP3, GRB2, CALM3, GNB5, PRKACB673164110912.41169861921501830.210913715631753430.[illegible]
REACTOME_PATHWAYR-HSA-9662851~Anti-inflammatory response favouring Leishmania parasite infection151.58562367864693442.4796986833086274E-4SRC, PLK2, GNAI3, ITPR3, GNG12, MAPK14, IL6, ADAM17, CREB1, ADORA2B, GNB2, GNB4, CALM3, GNB5, PRKACB67379110913.1291026388549290.349189695566722860.00558093223139[illegible]

[illegible]

REACTOME_PATHWAYR-HSA-389957~Prefoldin mediated transfer of substrate to CCT/TriC30.31712473572938690.5127007907387976TCP1, CCT7, CCT567328110911.7657079176395671.00.9333093175948910.8299074774458898
REACTOME_PATHWAYR-HSA-389958~Cooperation of Prefoldin and TriC/CCT in actin and tubulin folding30.31712473572938690.5857473140255957TCP1, CCT7, CCT567332110911.5449944279346211.01.00.8897228637413395

Annotation Cluster 55Enrichment Score: 0.9062656599891419
CategoryTermCount%PValueGenesList TotalPop HitsPop TotalFold EnrichmentBonferroniBenjaminiFDR
REACTOME_PATHWAYR-HSA-1483255~PI Metabolism101.05708245243128960.06710950022946216INPP4A, TNFAIP8, MTMR3, RAB14, PTEN, INPPL1, PI4KB, PIK3R1, SBF1, PIK3CG67384110911.96189768626618541.00.27916899399251605 0.24823971133437225
KEGG_PATHWAYhsa04070:Phosphatidylinositol signaling system111.16279069767441870.07758182516987255CDS1, INPP4A, ITPKC, MTMR3, IMPA1, PTEN, INPPL1, CALM3, ITPR3, PI4KB, PIK3R15449888401.82397959183673470.99999999999401710.19385249700405696 0.1312606249306 4672
REACTOME_PATHWAYR-HSA-1660499~Synthesis of PIPs at the plasma membrane70.73995771670190270.09970999370166843INPP4A, MTMR3, RAB14, PTEN, INPPL1, PIK3R1, PIK3CG67353110912.17659592363116431.00.36532223908032010.3248479921654779
KEGG_PATHWAYhsa00562:Inositol phosphate metabolism80.84566596194503170.15983527446366508INPP4A, ITPKC, MTMR3, IMPA1, PTEN, INPPL1, PI4KB, PIK3CG5447388401.78082191780821921.00.34064292849797695 0.2306547729014515
REACTOME_PATHWAYR-HSA-1483249~Inositol phosphate metabolism60.63424947145877380.18403190565733385INPP4A, ITPKC, PTEN, INPPL1, CALM367350110911.97759286775631481.00.52490785365873150.4667530308644577
REACTOME_PATHWAYR-HSA-1855204~Synthesis of IP3 and IP4 in the cytosol40.42283298097251587 0.23908790125611473ITPKC, PTEN, INPPL1, CALM367328110912.35427722351942271.00.59531513344374540.5293598503386103

Annotation Cluster 56Enrichment Score: 0.883948539238031
CategoryTermCount%PValueGenesList TotalPop HitsPop TotalFold EnrichmentBonferroniBenjaminiFDR
REACTOME_PATHWAYR-HSA-2029480~Fcgamma receptor (FCGR) dependent phagocytosis101.05708245243128960.07551968771850989VAV3, HSP90AA1, MYH2, MYO1C, SRC, ELMO1, GRB2, ITPR3, WASL, PIK3R167386110911.91627215867859981.00.30365572811178110.2700135470399623
REACTOME_PATHWAYR-HSA-9664417~Leishmania phagocytosis70.73995771670190270.14570050442669302VAV3, MYH2, MYO1C, SRC, ELMO1, GRB2, WASL67359110911.95524718563477441.00.45825585149085120.40748543978499807
REACTOME_PATHWAYR-HSA-9664422~FCGR3A-mediated phagocytosis70.73995771670190270.14570050442669302VAV3, MYH2, MYO1C, SRC, ELMO1, GRB2, WASL67359110911.95524718563477441.00.45825585149085120.40748543978499807
REACTOME_PATHWAYR-HSA-9664407~Parasite infection70.73995771670190270.14570050442669302VAV3, MYH2, MYO1C, SRC, ELMO1, GRB2, WASL67359110911.95524718563477441.00.45825585149085120.40748543978499807
REACTOME_PATHWAYR-HSA-2029482~Regulation of actin dynamics for phagocytic cup formation70.73995771670190270.16285982071572103VAV3, HSP90AA1, MYH2, MYO1C, ELMO1, GRB2, WASL67361110911.89114072053199521.00.48761973766964500.43359608525615867

Annotation Cluster 57Enrichment Score: 0.8504726343551168
CategoryTermCount%PValueGenesList TotalPop HitsPop TotalFold EnrichmentBonferroniBenjaminiFDR
REACTOME_PATHWAYR-HSA-432722~Golgi Associated Vesicle Biogenesis70.73995771670190270.12162704656143179TGOLN2, NECAP1, AP1G1, HIP1R, CLTA, AP4B1, TXNDC567356110912.05999257057949510.00.40928091590477920.3639364635944979
REACTOME_PATHWAYR-HSA-199992~trans-Golgi Network Vesicle Budding80.84566596194503170.14471800303282062TGOLN2, NECAP1, AP1G1, HIP1R, CLTA, AP4B1, GNS, TXNDC567372110911.83110450718177310.00.45825585149085120.40748543978499807
REACTOME_PATHWAYR-HSA-432720~Lysosome Vesicle Biogenesis50.52854122621564480.15959870931935793AP1G1, CLTA, AP4B1, GNS, TXNDC567335110912.35427722351942271.00.48317552726939667 0.4296442513111023

Annotation Cluster 58Enrichment Score: 0.8446071726609711
CategoryTermCount%PValueGenesList TotalPop HitsPop TotalFold EnrichmentBonferroniBenjaminiFDR
REACTOME_PATHWAYR-HSA-5627123~RHO GTPases activate PAKs60.63424947145877380.01305773030122007 3PPP1CB, FLNA, CALM3, MYH10, PAK2, MYL967324110914.11998514115898 90.99999999987018930.10509031113999048 0.09344729917295172
REACTOME_PATHWAYR-HSA-5625900~RHO GTPases activate CIT40.42283298097251587 0.11735322431142463PPP1CB, MYH10, MYL9, RHOB67320110913.29598811292719151.00.39931500304309430.35507467956688304
REACTOME_PATHWAYR-HSA-5627117~RHO GTPases Activate ROCKs40.42283298097251587 0.11735322431142463PPP1CB, MYH10, MYL9, RHOB67320110913.29598811292719151.00.39931500304309430.35507467956688304
REACTOME_PATHWAYR-HSA-400685~Sema4D in semaphorin signaling40.42283298097251587 0.19071301611235764MYH10, MET, MYL9, RHOB67325110912.63679049034175310.537134711245265400.47762526833753827
REACTOME_PATHWAYR-HSA-416572~Sema4D induced cell migration and growth-cone collapse30.31712473572938690.36642154999556 9MYH10, MYL9, RHOB67321110912.35427722351942271.00.76877547959118760.6836024316503291
REACTOME_PATHWAYR-HSA-5625740~RHO GTPases activate PKNs60.63424947145877380.68098551580025PPP1CB, KDM1A, MYH10, MYL9, YWHAZ, RHOB67394110911.05191109987038021.01.00.8897228637413395

Annotation Cluster 59Enrichment Score: 0.787604233523934
CategoryTermCount%PValueGenesList TotalPop HitsPop TotalFold EnrichmentBonferroniBenjaminiFDR
REACTOME_PATHWAYR-HSA-5083630~Defective LFNG causes SCDO330.31712473572938690.03242701471205964 4LFNG, NOTCH2, NOTCH16735110919.88796433878157510.0.177716170797321470.158026900864785
KEGG_PATHWAYhsa04330:Notch signaling pathway70.73995771670190270.17929766473558822LFNG, NOTCH2, ADAM17, ITCH, NOTCH1, EP300, HES15446288401.83467741935483851.00.37382977157289310.2531261149208304
REACTOME_PATHWAYR-HSA-1912420~Pre-NOTCH Processing in Golgi30.31712473572938690.2984861943627842LFNG, NOTCH2, NOTCH167318110912.74665676077265971.00.60512381400619890.60601742491838
REACTOME_PATHWAYR-HSA-3906995~Diseases associated with O-glycosylation of proteins60.63424947145877380.40754654460860984LFNG, NOTCH2, ADAMTS5, NOTCH1, THBS2, THBS167369110911.43303830996834411.00.81813979088184900.721872672691802

Annotation Cluster 60Enrichment Score: 0.7779814045019772
CategoryTermCount%PValueGenesList TotalPop HitsPop TotalFold EnrichmentBonferroniBenjaminiFDR
KEGG_PATHWAYhsa04120:Ubiquitin mediated proteolysis171.79704016913319230.01338789627941001FBXW7, CUL1, XIAP, UBE2D1, WWP2, UBE2G1, CUL4A, CDC20, HERC3, ITCH, CDC27, UBR5, MDM2, UBA1, VHL, RPS27A, CUL4B54414288401.94542253521126770.98660753557060790.04495514645402220.0304398483826608
REACTOME_PATHWAYR-HSA-1280218~Adaptive Immune System535.60253699788583502.15081027986294 91ITGB1, PTEN, UBE2D1, CLTA, CDC20, NPEPPS, HERC3, PPP2R5E, AP1G1, CDH1, MRC1, CDC27, PRKACB, RNF111, CTSB, TRIM63, CHUK, FBXW7, KIF22, DYNLL2, YWHAZ, PJA2, KIF20A, TLR6, RAF1, SOS1, PSMD14, SRC, CUL1, ITPR3, PIK3R1, RAP1B, NRAS, BTLA, VHL, LONRF1, RPS27A, PAK2, SEC24A, VCAM1, KLHL2, PTPN11, UBE2G1, ITCH, PSMC3, RNF182, HLA-DRA, KRAS, UBA1, CALM3, GRB2, TAB2, SIGLEC1673768110911.13728756500742941.00.57698981656385310.5130648051499698
REACTOME_PATHWAYR-HSA-983168~Antigen processing: Ubiquitination & Proteasome degradation202.11416490486257920.49785692856577424TRIM63, PSMD14, FBXW7, CUL1, KLHL2, UBE2D1, UBE2G1, PJA2, CDC20, NPEPPS, HERC3, ITCH, PSMC3, CDC27, RNF182, UBA1, VHL, LONRF1, RPS27A, RNF111673309110911.06666281971753761.00.91688210117373730.8153002411475644
REACTOME_PATHWAYR-HSA-983169~Class I MHC mediated antigen processing & presentation242.53699788583509 50.5390826863838623TRIM63, SEC24A, PSMD14, CHUK, FBXW7, CUL1, KLHL2, UBE2D1, UBE2G1, PJA2, CDC20, NPEPPS, HERC3, ITCH, PSMC3, CDC27, MRC1, RNF182, UBA1, VHL, TLR6, LONRF1, RPS27A, RNF111673381110911.03810649226053281.00.9599257695228950.8535750783812933

Annotation Cluster 61Enrichment Score: 0.7512917969156204
CategoryTermCount%PValueGenesList TotalPop HitsPop TotalFold EnrichmentBonferroniBenjaminiFDR
REACTOME_PATHWAYR-HSA-9734009~Defective Intrinsic Pathway for Apoptosis50.52854122621564480.06131723349595063PRDX1, FOXO3, SOD2, TP53, LMNB167325110913.29598811292719151.00.26433523793154834 0.23504939506781075
REACTOME_PATHWAYR-HSA-8862803~Deregulated CDK5 triggers multiple neurodegenerative pathways in Alzheimer's disease models40.42283298097251587 0.14531437580213 14PRDX1, FOXO3, SOD2, LMNB167322110912.99635282993381 11.00.45825585149085120.40748543978499807
REACTOME_PATHWAYR-HSA-8863678~Neurodegenerative Diseases40.42283298097251587 0.14531437580213 14PRDX1, FOXO3, SOD2, LMNB167322110912.99635282993381 11.00.45825585149085120.40748543978499807
REACTOME_PATHWAYR-HSA-9645723~Diseases of programmed cell death60.63424947145877380.7631907512212651PRDX1, FOXO3, SOD2, TP53, LMNB1, EZH267310411091 0.95076580180592051.01.00.8897228637413395

Annotation Cluster 62Enrichment Score: 0.748330593178002
CategoryTermCount%PValueGenesList TotalPop HitsPop TotalFold EnrichmentBonferroniBenjaminiFDR
REACTOME_PATHWAYR-HSA-352230~Amino acid transport across the plasma membrane60.63424947145877380.04671376896834707SLC6A6, SLC7A5, SLC6A15, SLC43A1, SLC7A11, SLC7A267333110912.99635282993381051.00.22092851527084173 0.1964517265045396
REACTOME_PATHWAYR-HSA-425393~Transport of inorganic cations/anions and amino acids/oligopeptides101.05708245243128960.22225347656893724SLC6A6, SLC7A5, SLC26A2, SLC9A6, SLC6A15, SLC43A1, CALM3, SLC7A11, SLC12A7, SLC7A26731101 10911.49817641496690521.00.57778048477195090.5137678748029869
REACTOME_PATHWAYR-HSA-425407~SLC-mediated transmembrane transport161.69133192389006350.5479169666081745SLC26A2, SLC16A1, SLC6A15, SLC43A1, SLC7A11, SLC2A4, SLC7A2, RUNX1, SLC5A6, SLC6A6, SLC7A5, SLC9A6, CALM3, SLC25A5, SLC12A7, SLC27A4673249110911.05895200415331451.00.97307371558058910.8652663564395199

Annotation Cluster 63Enrichment Score: 0.7308541280694086
CategoryTermCount%PValueGenesList TotalPop HitsPop TotalFold EnrichmentBonferroniBenjaminiFDR
KEGG_PATHWAYhsa05012:Parkinson disease242.53699788583509 50.07109081163404185PSMD14, APAF1, GNAI3, UBE2G1, ITPR3, SDHD, NDUFC2-KCTD14, MAPK9, PINK1, PSMC3, NDUFS6, PPIF, UQCRFS1, CALM3, UBA1, UQCRC2, NDUFV3, SLC25A5, RPS27A, TP53, PRKACB, MCU, NFE2L2, BCL2L154427188401.43911439114391150.99999999994357870.18288684605854316 0.12383560736252451
KEGG_PATHWAYhsa05010:Alzheimer disease323.38266384778012700.08475464703619287GSK3B, PSMD14, LRP5, ITPR3, PIK3R1, NDUFC2-KCTD14, MAPK9, NRAS, UQCRFS1, NDUFV3, FZD5, CHUK, APAF1, FZD7, FZD9, WNT9B, SDHD, DKK2, ADAM17, IL6, APC, PSMC3, NDUFS6, PPIF, FAS, KRAS, CALM3, UQCRC2, RAF1, SLC25A5, GAPDH, MCU54439188401.32992327365728990.99999999999995080.20638727026370632 0.13974812030395162
KEGG_PATHWAYhsa05022:Pathways of neurodegeneration - multiple diseases384.01691331923890.09412786332421427GSK3B, PSMD14, LRP5, DERL1, ITPR3, NDUFC2-KCTD14, MAPK9, NRAS, UQCRFS1, NDUFV3, RPS27A, FZD5, APAF1, FZD7, FZD9, WNT9B, UBE2G1, SDHD, MAPK14, TOMM40L, DKK2, IL6, PINK1, APC, PSMC3, NDUFS6, CHMP2B, PPIF, FAS, KRAS, UBA1, CALM3, UQCRC2, RAF1, TARDBP, SLC25A5, MCU, BCL2L154448388401.27846790890269160.99999999999998180.22747566970018449 0.15402741271235063
KEGG_PATHWAYhsa05020:Prion disease212.21987315010570850.26166383183903 95GSK3B, NOTCH1, PSMD14, APAF1, ITPR3, PIK3R1, SDHD, MAPK14, NDUFC2-KCTD14, MAPK9, IL6, CREB1, PSMC3, NDUFS6, PPIF, UQCRFS1, UQCRC2, NDUFV3, SLC25A5, PRKACB, MCU54427888401.22751798561151081.00.51525161948551610.34888510911871934
KEGG_PATHWAYhsa05014:Amyotrophic lateral sclerosis242.53699788583509 50.5095293997930099NUP133, PSMD14, APAF1, DERL1, NUP153, ITPR3, SDHD, MAPK14, NDUFC2-KCTD14, TOMM40L, NUP93, PINK1, POM121C, PSMC3, NDUFS6, HNRNPA2B1, CHMP2B, UQCRFS1, UQCRC2, NDUFV3, TARDBP, TP53, MCU, BCL2L154437188401.05121293800539110.0.82928509456107240.5615221956902557
KEGG_PATHWAYhsa05016:Huntington disease202.11416490486257920.5448376289072931PSMD14, APAF1, CLTA, SDHD, SOD2, NDUFC2-KCTD14, MAPK9, CREB1, PSMC3, SP1, NDUFS6, PPIF, EP300, UQCRFS1, PPARG, UQCRC2, NDUFV3, SLC25A5, PPARGC1A, TP5354431188401.04501607717041810.0.86901601810713260.5884246392198766

Annotation Cluster 64Enrichment Score: 0.7248835769573849
CategoryTermCount%PValueGenesList TotalPop HitsPop TotalFold EnrichmentBonferroniBenjaminiFDR
REACTOME_PATHWAYR-HSA-2454202~Fc epsilon receptor (FCERI) signaling202.11416490486257924.4299599485040964E-4VAV3, MAP2K4, PSMD14, CHUK, SHC1, CUL1, UBE2D1, ITPR3, PIK3R1, MAPK9, NRAS, PSMC3, TAB3, GRB2, KRAS, CALM3, TAB2, RPS27A, SOS1, PAK2673134110912.45969262158745605.53579966424763880.008625978191862472 0.007670301438926755
REACTOME_PATHWAYR-HSA-69202~Cyclin E associated events during G1/S transition 131.37420718816067660.004087877102007173RB1, LINS4, PSMD14, CUL1, CCNA2, WEE1, PSMC3, CCND1, MYC, CDK2, E2F1, E2F4, RPS27A67383110912.58119551012370430.99917052219071810.04919646540123910.04374596259856287
REACTOME_PATHWAYR-HSA-69656~Cyclin A:Cdk2-associated events at S phase entry131.37420718816067660.004974821671806 56RB1, LINS4, PSMD14, CUL1, CCNA2, WEE1, PSMC3, CCND1, MYC, CDK2, E2F1, E2F4, RPS27A67385110912.52046149812079400.99982274033975530.05526516578573127 0.04914230841073969
REACTOME_PATHWAYR-HSA-5621481~C-type lectin receptors (CLRs)181.90274841437632140.005799695495834332PSMD14, CHUK, SRC, CUL1, UBE2D1, ITPR3, NRAS, RPS6KA5, PSMC3, EP300, TAB3, KRAS, CALM3, TAB2, RAF1, RPS27A, PAK2, PRKACB673143110912.07439811303109960.99995785164489390.061051936454364165 0.05428795965157253
REACTOME_PATHWAYR-HSA-69231~Cyclin D associated events in G190.95137420718816070.006693289586115743RB1, CCND3, CCND1, SRC, CDK2, CUL1, E2F1, E2F4, RPS27A67347110913.15573329961114050.99999912035043250.06590608439056012 0.05860431393297932
REACTOME_PATHWAYR-HSA-69236~G1 Phase90.95137420718816070.006693289586115743RB1, CCND3, CCND1, SRC, CDK2, CUL1, E2F1, E2F4, RPS27A67347110913.15573329961114050.99999912035043250.06590608439056012 0.05860431393297932
REACTOME_PATHWAYR-HSA-69273~Cyclin A/B1/B2 associated events during G2/M transition50.52854122621564480.01552844869294033CCNB2, WEE1, XPO1, CDK2, FOXM167316110916.19511875735526990.99999999999990.11451405089174582 0.10182697774043874
REACTOME_PATHWAYR-HSA-9659787~Aberrant regulation of mitotic G1/S transition in cancer due to RB1 defects50.52854122621564480.01687531151704854RB1, CCND3, CCND1, CDK2, E2F167317110915.84704134253998 70.99999999999984220.12084675561588892 0.10745807870980083
REACTOME_PATHWAYR-HSA-9661069~Defective binding of RB1 mutants to E2F1,(E2F2, E2F3)50.52854122621564480.01687531151704854RB1, CCND3, CCND1, CDK2, E2F167317110915.84704134253998 70.99999999999984220.12084675561588892 0.10745807870980083
REACTOME_PATHWAYR-HSA-9687139~Aberrant regulation of mitotic cell cycle due to RB1 defects70.73995771670190270.01741299070772211 3RB1, CCND3, CCND1, CDC27, CDK2, E2F1, UBE2D167335110913.29598811292719150.99999999999993880.12367505285445361 50.10997302737950729
REACTOME_PATHWAYR-HSA-5687128~MAPK6/MAPK4 signaling121.26849894291754750.01827648714766683 8CCND3, XPO1, PSMD14, PSMC3, MYC, FOXO3, RPS27A, PAK2, PRKACB, FOXO1, TNRC6A, TNRC6B67389110912.22201445815316270.99999999999998670.12782616844082645 0.11366423864241983
REACTOME_PATHWAYR-HSA-9675126~Diseases of mitotic cell cycle70.73995771670190270.02252616742688826 4RB1, CCND3, CCND1, CDC27, CDK2, E2F1, UBE2D167337110913.11782659330950551.00.14334728236579680.12746576002636634
REACTOME_PATHWAYR-HSA-69242~S Phase171.79704016913319230.03650178825287486RB1, GSK3B, LINS4, PSMD14, CUL1, UBE2D1, PDS5B, CCNA2, WEE1, PSMC3, CCND1, MYC, CDC27, CDK2, E2F1, E2F4, RPS27A673162110911.72937647900500 81.00.19168969406737010.17045228999297019
REACTOME_PATHWAYR-HSA-174143~APC/C-mediated degradation of cell cycle proteins111.16279069767441870.03706533160756422RB1, CCNA2, CDC20, PSMD14, PSMC3, CDC27, CUL1, UBE2D1, BUB3, RPS27A67387110912.08367064610339710.00.19347656528888190.17204119279294117
REACTOME_PATHWAYR-HSA-453276~Regulation of mitotic cell cycle111.16279069767441870.03706533160756422RB1, CCNA2, CDC20, PSMD14, PSMC3, CDC27, CUL1, UBE2D1, BUB3, RPS27A67387110912.08367064610339710.00.19347656528888190.17204119279294117
REACTOME_PATHWAYR-HSA-69563~p53-Dependent G1 DNA Damage Response90.95137420718816070.04504120816912273CCNA2, PSMD14, PSMC3, CHEK2, CDK2, MDM2, ATM, RPS27A, TP5367366110912.24726462245035 81.00.21925958920530814 0.1949677016534217
REACTOME_PATHWAYR-HSA-69580~p53-Dependent G1/S DNA damage checkpoint90.95137420718816070.04504120816912273CCNA2, PSMD14, PSMC3, CHEK2, CDK2, MDM2, ATM, RPS27A, TP5367366110912.24726462245035 81.00.21925958920530814 0.1949677016534217
REACTOME_PATHWAYR-HSA-176408~Regulation of APC/C activators between G1/S and early anaphase101.05708245243128960.05212573760142 5CCNA2, CDC20, PSMD14, PSMC3, CDC27, CDK2, CUL1, UBE2D1, BUB3, RPS27A67380110912.05999257057949461.00.23703788055457310.21077632656352976
REACTOME_PATHWAYR-HSA-69615~G1/S DNA Damage Checkpoints90.95137420718816070.05224955012801322 4CCNA2, PSMD14, PSMC3, CHEK2, CDK2, MDM2, ATM, RPS27A, TP5367368110912.18116860414299431.00.23703788055457310.21077632656352976
REACTOME_PATHWAYR-HSA-179409~APC-Cdc20 mediated degradation of Nek2A50.52854122621564480.06131723349595063CDC20, CDC27, UBE2D1, BUB3, RPS27A67325110913.29598811292719151.00.26433523793154834 0.23504939506781075
REACTOME_PATHWAYR-HSA-453279~Mitotic G1 phase and G1/S transition151.58562367864693440.06763856619226599RB1, LINS4, PSMD14, SRC, CUL1, CCNA2, CCND3, WEE1, PSMC3, CCND1, MYC, CDK2, E2F1, E2F4, RPS27A673149110911.65905441925865361.00.27916899399251605 0.24823971133437225
REACTOME_PATHWAYR-HSA-8939902~Regulation of RUNX2 expression and activity90.95137420718816070.06878543591160088GSK3B, PSMD14, PSMC3, STAT1, CUL1, RPS27A, PPARGC1A, PPARGC1B, RUNX267372110912.05999257057949461.00.27916899399251605 0.24823971133437225
REACTOME_PATHWAYR-HSA-983705~Signaling by the B Cell Receptor (BCR)121.26849894291754750.07653292377751417NRAS, PSMD14, PSMC3, CHUK, CUL1, GRB2, KRAS, CALM3, ITPR3, PIK3R1, RPS27A, SOS1673112110911.76570791763956710.00.30560266568302320.27174478235287863
REACTOME_PATHWAYR-HSA-1234174~Cellular response to hypoxia90.95137420718816070.08307480785148152EGLN3, PSMD14, PSMC3, EP300, UBE2D1, VHL, HIF1AN, RPS27A, HIF1A67375110911.97759286775631510.00.32279964575474770.2870364997738409
REACTOME_PATHWAYR-HSA-5607764~CLEC7A (Dectin-1) signaling111.16279069767441870.08486887302599094PSMD14, PSMC3, CHUK, SRC, CUL1, TAB3, UBE2D1, CALM3, TAB2, ITPR3, RPS27A673101110911.79484501199005510.00.32756738742548397 0.29127602078630743
REACTOME_PATHWAYR-HSA-382556~ABC-family proteins mediated transport111.16279069767441870.09382704248464765ABCC4, ABCC1, ABCB1, PSMD14, PSMC3, DERL1, RPS27A, EIF2S2, ABCD1, CFTR, ABCF1673103110911.75999365253393751.00.35271640916679910.31363876891288944
REACTOME_PATHWAYR-HSA-69206~G1/S Transition131.37420718816067660.09939527223302355RB1, LINS4, PSMD14, CUL1, CCNA2, WEE1, PSMC3, CCND1, MYC, CDK2, E2F1, E2F4, RPS27A673131110911.63541394916234721.00.36506401254175996 0.3246183746837
REACTOME_PATHWAYR-HSA-141405~Inhibition of the proteolytic activity of APC/C required for the onset of anaphase by mitotic spindle checkpoint components40.42283298097251587 0.11735322431142463CDC20, CDC27, UBE2D1, BUB367320110913.29598811292719151.00.39931500304309430.35507467956688304
REACTOME_PATHWAYR-HSA-141430~Inactivation of APC/C via direct inhibition of the APC/C complex40.42283298097251587 0.11735322431142463CDC20, CDC27, UBE2D1, BUB367320110913.29598811292719151.00.39931500304309430.35507467956688304
REACTOME_PATHWAYR-HSA-69541~Stabilization of p5370.73995771670190270.12942005141607593PSMD14, PSMC3, CHEK2, MDM2, ATM, RPS27A, TP5367355110912.02385235004301 21.00.42639724164269880.37915646241857986
REACTOME_PATHWAYR-HSA-202424~Downstream TCR signaling101.05708245243128960.13885655302374 26PSMD14, PSMC3, CHUK, PTEN, CUL1, HLA-DRA, UBE2D1, TAB2, PIK3R1, RPS27A67398110911.68162658822815911.00.44728328325305940.3977285282706085
REACTOME_PATHWAYR-HSA-174184~Cdc20:Phospho-APC/C mediated degradation of Cyclin A80.84566596194503170.14471800303282062CCNA2, CDC20, PSMD14, PSMC3, CDC27, UBE2D1, BUB3, RPS27A67372110911.83110450718177310.00.45825585149085120.40748543978499807
REACTOME_PATHWAYR-HSA-453274~Mitotic G2-G2/M phases171.79704016913319230.15094584932300592LINS4, HSP90AA1, PSMD14, CUL1, FOXM1, CCNA2, PPP1CB, CCNB2, WEE1, XPO1, PSMC3, CDK2, E2F1, EP300, CEP192, RPS27A, TP53673199110911.40783411858699141.00.47048409510210300.4183589097243743
REACTOME_PATHWAYR-HSA-179419~APC:Cdc20 mediated degradation of cell cycle proteins prior to satisfation of the cell cycle checkpoint80.84566596194503170.15228542872023 29CCNA2, CDC20, PSMD14, PSMC3, CDC27, UBE2D1, BUB3, RPS27A67373110911.80602088379572151.00.47354215525097870.4210773301351274 4
REACTOME_PATHWAYR-HSA-187577~SCF(Skp2)-mediated degradation of p27/p2170.73995771670190270.15417405205863421CCNA2, CCND1, PSMD14, PSMC3, CDK2, CUL1, RPS27A67360110911.92265973254086161.00.47457128282015470.4219932757057821
REACTOME_PATHWAYR-HSA-174048~APC/C:Cdc20 mediated degradation of Cyclin B40.42283298097251587 0.16003621041698424CDC20, CDC27, UBE2D1, RPS27A67323110912.86607619933668831.00.48317552726939667 0.4296442513111023
REACTOME_PATHWAYR-HSA-176409~APC/C:Cdc20 mediated degradation of mitotic proteins80.84566596194503170.16795505050779092CCNA2, CDC20, PSMD14, PSMC3, CDC27, UBE2D1, BUB3, RPS27A67375110911.75780326894502 11.00.49840086049657 8170.4431827616994962
REACTOME_PATHWAYR-HSA-176814~Activation of APC/C and APC/C:Cdc20 mediated degradation of mitotic proteins80.84566596194503170.17604626926685812CCNA2, CDC20, PSMD14, PSMC3, CDC27, UBE2D1, BUB3, RPS27A67376110911.73473058575115351.00.50932918971530070.4529003354594797
REACTOME_PATHWAYR-HSA-5619115~Disorders of transmembrane transporters151.58562367864693440.18048408544614764ABCA1, SLC26A2, SLC16A1, NUP133, PSMD14, DERL1, NUP153, HK1, NUP93, PSMC3, SLC9A6, RPS27A, ABCD1, CFTR, SLC27A4673176110911.40454038903147381.00.51956631242221570.46200328184802913
REACTOME_PATHWAYR-HSA-202403~TCR signaling111.16279069767441870.19092612932895756PSMD14, PSMC3, CHUK, PTEN, CUL1, HLA-DRA, UBE2D1, TAB2, PIK3R1, RPS27A, PAK2673120110911.51066121842496310.00.53713471124526540.4776252683375382 7
REACTOME_PATHWAYR-HSA-9762114~GSK3B and BTRC:CUL1-mediated-degradation of NFE2L260.63424947145877380.20547097242933288GSK3B, PSMD14, PSMC3, CUL1, RPS27A, NFE2L267352110911.90153160361184110.00.56236306495676360.500058559202754
REACTOME_PATHWAYR-HSA-1234176~Oxygen-dependent proline hydroxylation of Hypoxia-inducible Factor Alpha70.73995771670190270.20918099755299424EGLN3, PSMD14, PSMC3, UBE2D1, VHL, RPS27A, HIF1A67366110911.74787248412805610.00.56290476515425310.5005402441446648
REACTOME_PATHWAYR-HSA-69275~G2/M Transition161.69133192389006350.21462383008806718LINS4, HSP90AA1, PSMD14, CUL1, FOXM1, CCNA2, PPP1CB, CCNB2, WEE1, XPO1, PSMC3, CDK2, EP300, CEP192, RPS27A, TP53673197110911.33847233012271731.00.57665596518235730.5127679413421884
REACTOME_PATHWAYR-HSA-9604323~Negative regulation of NOTCH4 signaling60.63424947145877380.22768377642519 83PSMD14, PSMC3, FBXW7, CUL1, RPS27A, YWHAZ67354110911.83110450718177291.00.58153089001276790.5171027706345501
REACTOME_PATHWAYR-HSA-2871837~FCERI mediated NF-kB activation80.84566596194503170.22779891522475615PSMD14, PSMC3, CHUK, CUL1, TAB3, UBE2D1, TAB2, RPS27A67382110911.60779907947667881.00.58153089001276790.5171027706345501
REACTOME_PATHWAYR-HSA-1168372~Downstream signaling events of B Cell Receptor (BCR)80.84566596194503170.23689696293572 66NRAS, PSMD14, PSMC3, CHUK, CUL1, KRAS, CALM3, RPS27A67383110911.58842800622997181.00.59531513344374540.5293598503386103
REACTOME_PATHWAYR-HSA-8948751~Regulation of PTEN stability and activity70.73995771670190270.23896319374386943PSMD14, PSMC3, PTEN, XIAP, WWP2, TRIM27, RPS27A67369110911.67187802829640141.00.59531513344374540.5293598503386103
REACTOME_PATHWAYR-HSA-8941858~Regulation of RUNX3 expression and activity60.63424947145877380.23904471475906367PSMD14, PSMC3, SRC, MDM2, EP300, RPS27A67355110911.79781169796028631.00.59531513344374540.5293598503386103
REACTOME_PATHWAYR-HSA-69017~CDK-mediated phosphorylation and removal of Cdc670.73995771670190270.26992140188293545CCNA2, PSMD14, PSMC3, CDC27, CDK2, UBE2D1, RPS27A67372110911.60221644378405141.00.63903523150700430.5682361752754147
REACTOME_PATHWAYR-HSA-174178~APC/C:Cdh1 mediated degradation of Cdc20 and other APC/C:Cdh1 targeted proteins in late mitosis/early G170.73995771670190270.28045596664705863RB1, CDC20, PSMD14, PSMC3, CDC27, UBE2D1, RPS27A67373110911.58026827332125631.00.65591118785337740.5832424353618317
REACTOME_PATHWAYR-HSA-5610780~Degradation of GLI1 by the proteasome60.63424947145877380.29782129951836855ITCH, PSMD14, PSMC3, CUL1, RPS27A, PRKACB67360110911.64799405646359581.00.68152381400619890.60601742491838
REACTOME_PATHWAYR-HSA-5610783~Degradation of GLI2 by the proteasome60.63424947145877380.29782129951836855GSK3B, PSMD14, PSMC3, CUL1, RPS27A, PRKACB67360110911.64799405646359581.00.68152381400619890.60601742491838
REACTOME_PATHWAYR-HSA-5610785~GLI3 is processed to GLI3R by the proteasome60.63424947145877380.29782129951836855GSK3B, PSMD14, PSMC3, CUL1, RPS27A, PRKACB67360110911.64799405646359581.00.68152381400619890.60601742491838
REACTOME_PATHWAYR-HSA-69052~Switching of origins to a post-replicative state80.84566596194503170.31261838966880 87CCNA2, PSMD14, PSMC3, CDC27, CDK2, CUL1, UBE2D1, RPS27A67391110911.44878598370426 11.00.70321601939388660.6253063391812997
REACTOME_PATHWAYR-HSA-176407~Conversion from APC/C:Cdc20 to APC/C:Cdh1 in late anaphase30.31712473572938690.3217124966888174CDC20, CDC27, UBE2D167318110912.60209587862673 11.00.71291203849663340.6339281311732904
REACTOME_PATHWAYR-HSA-9687136~Aberrant regulation of mitotic exit in cancer due to RB1 defects30.31712473572938690.3213417249668817 4RB1, CDC27, UBE2D167319110912.60209587862673 11.00.71291203849663340.6339281311732904
REACTOME_PATHWAYR-HSA-5619084~ABC transporter disorders70.73995771670190270.33429649563611435ABCA1, PSMD14, PSMC3, DERL1, RPS27A, ABCD1, CFTR67378110911.47896902503432 11.00.73333648979415980.6520897465509522
REACTOME_PATHWAYR-HSA-2559582~Senescence-Associated Secretory Phenotype (SASP)90.95137420718816070.3495617256443578 3CCNA2, IL6, CXCL8, EHMT2, CDC27, STAT3, CDK2, UBE2D1, RPS27A673110110911.34835877347021471.00.75646619773053960.6726569017326955
REACTOME_PATHWAYR-HSA-174154~APC/C:Cdc20 mediated degradation of Securin60.63424947145877380.38309224778691 9CDC20, PSMD14, PSMC3, CDC27, UBE2D1, RPS27A67367110911.47581557295247381.00.79311397460940460.7052444517444272
REACTOME_PATHWAYR-HSA-349425~Autodegradation of the E3 ubiquitin ligase COP150.52854122621564480.38752802147853477PSMD14, PSMC3, ATM, RPS27A, TP5367352110911.58460966967653441.00.79311397460940460.7052444517444272
REACTOME_PATHWAYR-HSA-75815~Ubiquitin-dependent degradation of Cyclin D50.52854122621564480.38752802147853477GSK3B, CCND1, PSMD14, PSMC3, RPS27A67352110911.58460966967653441.00.79311397460940460.7052444517444272
REACTOME_PATHWAYR-HSA-195253~Degradation of beta-catenin by the destruction complex70.73995771670190270.38919975768958 75GSK3B, APC, PSMD14, PSMC3, PPP2R5E, CUL1, RPS27A67383110911.38987450545122541.00.79311397460940460.7052444517444272
REACTOME_PATHWAYR-HSA-5658442~Regulation of RAS by GAPs60.63424947145877380.39533035976135994NRAS, PSMD14, PSMC3, NF1, KRAS, RPS27A67368110911.45411240276199631.00.79478829868496150.7067332765571411
REACTOME_PATHWAYR-HSA-9020702~Interleukin-1 signaling90.95137420718816070.39621342756033895MAP2K4, PSMD14, PSMC3, CHUK, CUL1, TAB3, TAB2, RPS27A, SQSTM1673115110911.28973447897150971.00.79564063726774 90.7074911840909412
REACTOME_PATHWAYR-HSA-174113~SCF-beta-TrCP mediated degradation of Emi150.52854122621564480.42922642489507373CDC20, PSMD14, PSMC3, CUL1, RPS27A67355110911.49817641496690521.00.83998816627442880.7469254265602394
REACTOME_PATHWAYR-HSA-68949~Orc1 removal from chromatin60.63424947145877380.43186927648616047CCNA2, PSMD14, PSMC3, CDK2, CUL1, RPS27A67371110911.39267103363120761.00.83998816627442880.7469254265602394
KEGG_PATHWAYhsa04340:Hedgehog signaling pathway50.52854122621564480.45361076017158564GSK3B, GRK2, CCND1, CUL1, PRKACB5445688401.45089285714285721.00.75760121724992580.512983896319699
REACTOME_PATHWAYR-HSA-5676590~NIK-->noncanonical NF-kB signaling50.52854122621564480.48354486147874 72PSMD14, PSMC3, CHUK, CUL1, RPS27A67359110911.39660513259626741.00.91085135319855310.8099376429769016
REACTOME_PATHWAYR-HSA-4086400~PCP/CE pathway70.73995771670190270.48718697842437786PSMD14, PSMC3, FZD5, FZD7, CLTA, ROR1, RPS27A67392110911.25390852122230111.00.91512929690394120.8137416310034468
REACTOME_PATHWAYR-HSA-5607761~Dectin-1 mediated noncanonical NF-kB signaling50.52854122621564480.49681083018947PSMD14, PSMC3, CHUK, CUL1, RPS27A67360110911.37332838038632991.00.91688210117373730.8153002411475644
REACTOME_PATHWAYR-HSA-8852276~The role of GTSE1 in G2/M progression after G2 checkpoint60.63424947145877380.503247428821425CCNB2, HSP90AA1, PSMD14, PSMC3, RPS27A, TP5367377110911.28415121282877611.00.92227777430455950.8200981247566798
REACTOME_PATHWAYR-HSA-5678895~Defective CFTR causes cystic fibrosis50.52854122621564480.5099293844804259PSMD14, PSMC3, DERL1, RPS27A, CFTR67361110911.35081480037999671.00.93316538891718900.8297794947036287
REACTOME_PATHWAYR-HSA-174084~Autodegradation of Cdh1 by Cdh1:APC/C50.52854122621564480.53568651663618 65PSMD14, PSMC3, CDC27, UBE2D1, RPS27A67363110911.30793179084412371.00.95508717420834490.8492725536382 34
REACTOME_PATHWAYR-HSA-4608870~Asymmetric localization of PCP proteins50.52854122621564480.5483080873798838PSMD14, PSMC3, FZD5, FZD7, RPS27A67364110911.28749535661218411.00.97307371558058910.8652663564395199
REACTOME_PATHWAYR-HSA-5358351~Signaling by Hedgehog101.05708245243128960.5531370498995 94GSK3B, ADAM17, ITCH, GRK2, PSMD14, PSMC3, CUL1, RPS27A, PRKACB, CDC73673149110911.10603627950576911.00.97312206176877 00.8653093463275676
REACTOME_PATHWAYR-HSA-69481~G2/M Checkpoints111.16279069767441870.5621662750205862CCNB2, WEE1, PSMD14, PSMC3, CHEK2, CDK2, ATM, TOPBP1, RPS27A, TP53, YWHAZ673167110911.08550506713171 11.00.98011484367271220.8715273941 71754
REACTOME_PATHWAYR-HSA-1169091~Activation of NF-kappaB in B cells50.52854122621564480.5850577363640801PSMD14, PSMC3, CHUK, CUL1, RPS27A67367110911.22984637937281 01.00.8897228637413395
REACTOME_PATHWAYR-HSA-211733~Regulation of activated PAK-2p34 by proteasome mediated degradation40.42283298097251587 0.591033099804093 6PSMD14, PSMC3, RPS27A, PAK267350110911.31839524517087651.01.00.8897228637413395
REACTOME_PATHWAYR-HSA-5632684~Hedgehog 'on' state60.63424947145877380.5923066633263595ITCH, GRK2, PSMD14, PSMC3, RPS27A, CDC7367385110911.16328992220959711.01.00.8897228637413395
REACTOME_PATHWAYR-HSA-69239~Synthesis of DNA80.84566596194503170.5964676019726034CCNA2, PSMD14, PSMC3, CDC27, CDK2, CUL1, UBE2D1, RPS27A673120110911.09866270430906381.01.00.8897228637413395
REACTOME_PATHWAYR-HSA-69601~Ubiquitin Mediated Degradation of Phosphorylated Cdc25A40.42283298097251587 0.6179192426989331PSMD14, PSMC3, CHEK2, RPS27A67352110911.26768773574122751.01.00.8897228637413395
REACTOME_PATHWAYR-HSA-69610~p53-Independent DNA Damage Response40.42283298097251587 0.6179192426989331PSMD14, PSMC3, CHEK2, RPS27A67352110911.26768773574122751.01.00.8897228637413395
REACTOME_PATHWAYR-HSA-69613~p53-Independent G1/S DNA damage checkpoint40.42283298097251587 0.6179192426989331PSMD14, PSMC3, CHEK2, RPS27A67352110911.26768773574122751.01.00.8897228637413395
REACTOME_PATHWAYR-HSA-2565942~Regulation of PLK1 Activity at G2/M Transition60.63424947145877380.6233428064888198PPP1CB, CCNB2, HSP90AA1, CUL1, CEP192, RPS27A67388110911.12362331122517911.01.00.8897228637413395
REACTOME_PATHWAYR-HSA-169911~Regulation of Apoptosis40.42283298097251587 0.630904967659726PSMD14, PSMC3, RPS27A, PAK267353110911.24376909927808 1.01.00.8897228637413395
REACTOME_PATHWAYR-HSA-8854050~FBXL7 down-regulates AURKA during mitotic entry and in early mitosis40.42283298097251587 0.6559486918837261PSMD14, PSMC3, CUL1, RPS27A67355110911.19854113197352421.01.00.8897228637413395
REACTOME_PATHWAYR-HSA-5610787~Hedgehog 'off' state70.73995771670190270.6882343128580063GSK3B, ITCH, PSMD14, PSMC3, CUL1, RPS27A, PRKACB673113110911.02088127391550181.01.00.8897228637413395
REACTOME_PATHWAYR-HSA-5689603~UCH proteinases60.63424947145877380.74814771478384 02INO80D, PSMD14, PSMC3, RPS27A, TGFBR1, HCFC1673102110910.96940826850799761.01.00.8897228637413395
REACTOME_PATHWAYR-HSA-5358346~Hedgehog ligand biogenesis40.42283298097251587 0.7626756868970609ADAM17, PSMD14, PSMC3, RPS27A67365110911.01415018859298 21.01.00.8897228637413395
REACTOME_PATHWAYR-HSA-1236975~Antigen processing-Cross presentation60.63424947145877380.770453704503841 5PSMD14, PSMC3, CHUK, MRC1, TLR6, RPS27A673105110910.94171008940776911.01.00.8897228637413395
REACTOME_PATHWAYR-HSA-1236974~ER-Phagosome pathway50.52854122621564480.8028808167695491PSMD14, PSMC3, CHUK, TLR6, RPS27A67390110910.91555225359088641.01.00.8897228637413395
REACTOME_PATHWAYR-HSA-180534~Vpu mediated degradation of CD430.31712473572938690.8318735976908991PSMD14, PSMC3, RPS27A67352110910.95076580180592051.01.00.8897228637413395
REACTOME_PATHWAYR-HSA-180585~Vif-mediated degradation of APOBEC3G30.31712473572938690.8472998241917153PSMD14, PSMC3, RPS27A67354110910.91555225359088641.01.00.8897228637413395
REACTOME_PATHWAYR-HSA-4641257~Degradation of AXIN30.31712473572938690.854520621633892PSMD14, PSMC3, RPS27A67355110910.89890584898014311.01.00.8897228637413395
REACTOME_PATHWAYR-HSA-5362768~Hh mutants are degraded by ERAD30.31712473572938690.8614286419264064PSMD14, PSMC3, RPS27A67356110910.88285395881978351.01.00.8897228637413395
REACTOME_PATHWAYR-HSA-450408~AUF1 (hnRNP D0) binds and destabilizes mRNA30.31712473572938690.8614286419264064PSMD14, PSMC3, RPS27A67356110910.88285395881978351.01.00.8897228637413395
REACTOME_PATHWAYR-HSA-4641258~Degradation of DVL30.31712473572938690.8680352099597 97PSMD14, PSMC3, RPS27A67357110910.86736529287557671.01.00.8897228637413395
REACTOME_PATHWAYR-HSA-5668541~TNFR2 non-canonical NF-kB pathway50.52854122621564480.8733049301704273PSMD14, PSMC3, CHUK, CUL1, RPS27A673102110910.80784022375666461.01.00.8897228637413395
REACTOME_PATHWAYR-HSA-5387390~Hh mutants abrogate ligand secretion30.31712473572938690.8803881647308966PSMD14, PSMC3, RPS27A67359110910.83796307955776051.01.00.8897228637413395
REACTOME_PATHWAYR-HSA-69306~DNA Replication80.84566596194503170.9391634707156906CCNA2, PSMD14, PSMC3, CDC27, CDK2, CUL1, UBE2D1, RPS27A673186110910.70881464794133151.01.00.9391634707156906
REACTOME_PATHWAYR-HSA-69002~DNA Replication Pre-Initiation60.63424947145877380.966538140451455PSMD14, PSMC3, CDC27, CDK2, UBE2D1, RPS27A673158110910.62582052777098571.01.00.966538140451455
REACTOME_PATHWAYR-HSA-68867~Assembly of the pre-replicative complex50.52854122621564480.9747917325901718PSMD14, PSMC3, CDC27, UBE2D1, RPS27A673141110910.58439505548354461.01.00.9747917325901718

Annotation Cluster 65Enrichment Score: 0.7123386221031144
CategoryTermCount%PValueGenesList TotalPop HitsPop TotalFold EnrichmentBonferroniBenjaminiFDR
REACTOME_PATHWAYR-HSA-450282~MAPK targets/ Nuclear events mediated by MAP kinases50.52854122621564480.11527711227781075MAPK9, MEF2C, RPS6KA5, CREB1, MAPK1467331110912.65805492977999311.00.39931500304309430.35507467956688304
REACTOME_PATHWAYR-HSA-198725~Nuclear Events (kinase and transcription factor activation)70.73995771670190270.16285982071572103MEF2C, RPS6KA5, CREB1, ID4, EP300, MAPK14, F367361110911.89114072053199521.00.48761973766964500.43359608525615867
REACTOME_PATHWAYR-HSA-198753~ERK/MAPK targets30.31712473572938690.38853226741238 3MEF2C, RPS6KA5, MAPK1467322110912.24726462245035 81.00.79311397460940460.7052444517444272

Annotation Cluster 66Enrichment Score: 0.6958477208912537
CategoryTermCount%PValueGenesList TotalPop HitsPop TotalFold EnrichmentBonferroniBenjaminiFDR
REACTOME_PATHWAYR-HSA-4791275~Signaling by WNT in cancer60.63424947145877380.05215010935325891 5GSK3B, APC, FZD5, PPP2R5E, LRP5, DKK267334110912.90822480552399251.00.23703788055457310.21077632656352976
REACTOME_PATHWAYR-HSA-4641262~Disassembly of the destruction complex and recruitment of AXIN to the membrane50.52854122621564480.11527711227781075GSK3B, APC, FZD5, PPP2R5E, LRP567331110912.65805492977999311.00.39931500304309430.35507467956688304
REACTOME_PATHWAYR-HSA-4839735~Signaling by AXIN mutants30.31712473572938690.2067081779442922GSK3B, APC, PPP2R5E67314110913.53141583527913410.0.56236306495676360.500058559202754
REACTOME_PATHWAYR-HSA-4839744~Signaling by APC mutants30.31712473572938690.2067081779442922GSK3B, APC, PPP2R5E67314110913.53141583527913410.0.56236306495676360.500058559202754
REACTOME_PATHWAYR-HSA-4839748~Signaling by AMER1 mutants30.31712473572938690.2067081779442922GSK3B, APC, PPP2R5E67314110913.53141583527913410.0.56236306495676360.500058559202754
REACTOME_PATHWAYR-HSA-5467337~APC truncation mutants have impaired AXIN binding30.31712473572938690.2067081779442922GSK3B, APC, PPP2R5E67314110913.53141583527913410.0.56236306495676360.500058559202754
REACTOME_PATHWAYR-HSA-5467340~AXIN missense mutants destabilize the destruction complex30.31712473572938690.2067081779442922GSK3B, APC, PPP2R5E67314110913.53141583527913410.0.56236306495676360.500058559202754
REACTOME_PATHWAYR-HSA-5467348~Truncations of AMER1 destabilize the destruction complex30.31712473572938690.2067081779442922GSK3B, APC, PPP2R5E67314110913.53141583527913410.0.56236306495676360.500058559202754
REACTOME_PATHWAYR-HSA-5339716~Signaling by GSK3beta mutants30.31712473572938690.22952517528490088GSK3B, APC, PPP2R5E67315110913.29598811292719151.00.58153089001276790.5171027706345501
REACTOME_PATHWAYR-HSA-5358747~CTNNB1 S33 mutants aren't phosphorylated30.31712473572938690.22952517528490088GSK3B, APC, PPP2R5E67315110913.29598811292719151.00.58153089001276790.5171027706345501
REACTOME_PATHWAYR-HSA-5358749~CTNNB1 S37 mutants aren't phosphorylated30.31712473572938690.22952517528490088GSK3B, APC, PPP2R5E67315110913.29598811292719151.00.58153089001276790.5171027706345501
REACTOME_PATHWAYR-HSA-4839743~Signaling by CTNNB1 phospho-site mutants30.31712473572938690.22952517528490088GSK3B, APC, PPP2R5E67315110913.29598811292719151.00.58153089001276790.5171027706345501
REACTOME_PATHWAYR-HSA-5358751~CTNNB1 S45 mutants aren't phosphorylated30.31712473572938690.22952517528490088GSK3B, APC, PPP2R5E67315110913.29598811292719151.00.58153089001276790.5171027706345501
REACTOME_PATHWAYR-HSA-5358752~CTNNB1 T41 mutants aren't phosphorylated30.31712473572938690.22952517528490088GSK3B, APC, PPP2R5E67315110913.29598811292719151.00.58153089001276790.5171027706345501
REACTOME_PATHWAYR-HSA-196299~Beta-catenin phosphorylation cascade30.31712473572938690.2755101941849841GSK3B, APC, PPP2R5E67317110912.90822480552399251.00.64696364027449530.5752861913808408
REACTOME_PATHWAYR-HSA-195253~Degradation of beta-catenin by the destruction complex70.73995771670190270.38919975768958 75GSK3B, APC, PSMD14, PSMC3, PPP2R5E, CUL1, RPS27A67383110911.38987450545122541.00.79311397460940460.7052444517444272

Annotation Cluster 67Enrichment Score: 0.6538904988529719
CategoryTermCount%PValueGenesList TotalPop HitsPop TotalFold EnrichmentBonferroniBenjaminiFDR
REACTOME_PATHWAYR-HSA-5357905~Regulation of TNFR1 signaling70.73995771670190270.06832590002942002CHUK, SPPL2A, RACK1, TNFAIP3, UBE2D1, XIAP, RPS27A67348110912.40332466567607 71.00.27916899399251605 0.24823971133437225
REACTOME_PATHWAYR-HSA-168928~DDX58/IFIH1-mediated induction of interferon-alpha/beta80.84566596194503170.22779891522475615HSP90AA1, ITCH, CHUK, EP300, TNFAIP3, UBE2D1, RPS27A, ATG1267382110911.60779907947667881.00.58153089001276790.5171027706345501
REACTOME_PATHWAYR-HSA-168927~TICAM1, RIP1-mediated IKK complex recruitment30.31712473572938690.34400711393340 81CHUK, UBE2D1, RPS27A67320110912.47199108469539 41.00.74989223703974370.6668112736746942
REACTOME_PATHWAYR-HSA-937041~IKK complex recruitment mediated by RIP130.31712473572938690.45261982049354627CHUK, UBE2D1, RPS27A67325110911.97759286775631481.00.8754354340572720.7784454725229406

Annotation Cluster 68Enrichment Score: 0.6455429138492476
CategoryTermCount%PValueGenesList TotalPop HitsPop TotalFold EnrichmentBonferroniBenjaminiFDR
REACTOME_PATHWAYR-HSA-9663891~Selective autophagy90.95137420718816070.11648873087199796HSP90AA1, PINK1, PRKAA2, SRC, ATM, RPS27A, DYNLL2, ATG12, CFTR67381110911.83110450718177310.00.39931500304309430.35507467956688304
REACTOME_PATHWAYR-HSA-5205647~Mitophagy40.42283298097251587 0.25565850921996913PINK1, SRC, RPS27A, ATG1267329110912.27309525029461 51.00.61965901605343580.5510066611300314
REACTOME_PATHWAYR-HSA-5205685~PINK1-PRKN Mediated Mitophagy30.31712473572938690.38853226741238 3PINK1, RPS27A, ATG1267322110912.24726462245035 81.00.79311397460940460.7052444517444272

Annotation Cluster 69Enrichment Score: 0.5157100458580737
CategoryTermCount%PValueGenesList TotalPop HitsPop TotalFold EnrichmentBonferroniBenjaminiFDR
REACTOME_PATHWAYR-HSA-73887~Death Receptor Signaling161.69133192389006350.04645703962710845 4VAV3, BEX3, CHUK, SPPL2A, XIAP, TNFAIP3, UBE2D1, PREX1, ADAM17, RACK1, FAS, TAB3, TAB2, ECT2, RPS27A, SOS1673154110911.71220161710503451.00.22092851527084173 0.1964517265045396
REACTOME_PATHWAYR-HSA-204998~Cell death signalling via NRAGE, NRIF and NADE60.63424947145877380.4915693743394344VAV3, BEX3, PREX1, ECT2, RPS27A, SOS167376110911.30104793931336 51.00.91688210117373730.8153002411475644
REACTOME_PATHWAYR-HSA-193704~p75 NTR receptor-mediated signalling70.73995771670190270.5395081936037506VAV3, BEX3, PREX1, ADAM17, ECT2, RPS27A, SOS167397110911.18927406136548151.00.9599257695228950.8535750783812933
REACTOME_PATHWAYR-HSA-193648~NRAGE signals death through JNK40.42283298097251587 0.70230445314782 09VAV3, PREX1, ECT2, SOS167359110911.11728410607701410.1.00.8897228637413395

Annotation Cluster 70Enrichment Score: 0.5002398380686162
CategoryTermCount%PValueGenesList TotalPop HitsPop TotalFold EnrichmentBonferroniBenjaminiFDR
REACTOME_PATHWAYR-HSA-72203~Processing of Capped Intron-Containing Pre-mRNA232.43128964059196 60.14267558192564891DDX5, CPSF7, NUP133, NCBP2, SRSF1, HNRNPU, THOC3, SNU13, NUP153, PRPF8, HNRNPL, NUP93, LENG1, PNN, NUDT21, PRPF6, HNRNPK, HNRNPF, ZC3H11A, HNRNPA2B1, SNRNP27, SNRPC, TXNL4A673285110911.32996011574255110.0.45451614609770140.4041600583592371
REACTOME_PATHWAYR-HSA-72163~mRNA Splicing - Major Pathway161.69133192389006350.27308279501855 19DDX5, NCBP2, SRSF1, HNRNPU, SNU13, PRPF8, HNRNPL, LENG1, PNN, PRPF6, HNRNPK, HNRNPF, HNRNPA2B1, SNRNP27, SNRPC, TXNL4A673207110911.27381183108297251.00.64563776775873180.5741072129926174
REACTOME_PATHWAYR-HSA-72172~mRNA Splicing161.69133192389006350.32334037060493 59DDX5, NCBP2, SRSF1, HNRNPU, SNU13, PRPF8, HNRNPL, LENG1, PNN, PRPF6, HNRNPK, HNRNPF, HNRNPA2B1, SNRNP27, SNRPC, TXNL4A673215110911.22641418155430411.00.71291203849663340.6339281311732904
KEGG_PATHWAYhsa03040:Spliceosome121.26849894291754750.7920210498767541DDX5, PRPF6, HNRNPK, NCBP2, SRSF1, HNRNPU, SNRNP27, THOC3, SNU13, SNRPC, TXNL4A, PRPF854421888400.89449541284403681.01.00.7920210498767541

Annotation Cluster 71Enrichment Score: 0.4897759374039097 3
CategoryTermCount%PValueGenesList TotalPop HitsPop TotalFold EnrichmentBonferroniBenjaminiFDR
REACTOME_PATHWAYR-HSA-2980766~Nuclear Envelope Breakdown80.84566596194503170.03621165544695014NUP93, CCNB2, NUP133, NEK6, NUP153, LPIN1, LEMD2, LMNB167352110912.53537547148245510.0.19132560637062374 0.17012853976753098
REACTOME_PATHWAYR-HSA-162909~Host Interactions of HIV factors141.47991543340380540.05124258983786023NPM1, NUP133, PSMD14, CCNT1, NUP153, RANGAP1, NUP93, XPO1, PSMC3, AP1G1, ELMO1, SLC25A5, RPS27A, PAK2673130110911.77476283003771851.00.23492965129368196 0.20890166915381644
REACTOME_PATHWAYR-HSA-177243~Interactions of Rev with host cellular proteins60.63424947145877380.06408183838631 55NUP93, NPM1, XPO1, NUP133, NUP153, RANGAP167336110912.74665676077265971.00.27020395601821107 0.24026791472825348
REACTOME_PATHWAYR-HSA-70171~Glycolysis90.95137420718816070.07336798942231937NUP93, PKM, NUP133, NUP153, ALDOA, GAPDH, PRKACB, PGM2L1, HK167373110912.03177349427018711.00.29569005969506856 0.26293039930184686
REACTOME_PATHWAYR-HSA-3232142~SUMOylation of ubiquitinylation proteins60.63424947145877380.07741574169636845NUP93, NUP133, MDM2, NUP153, TRIM27, VHL67338110912.60209587862673 11.00.30713790429396290.27310993105423936
KEGG_PATHWAYhsa03013:Nucleocytoplasmic transport111.16279069767441870.12623516357012 17NUP93, EEF1A1, PNN, XPO1, NUP133, POM121C, NCBP2, THOC3, NUP153, RANGAP1, TNPO15441088840 1.65509259259259281.00.28763583699192020.19476282379390208
REACTOME_PATHWAYR-HSA-4615885~SUMOylation of DNA replication proteins60.63424947145877380.13453707863053 43NUP93, NUP133, CDCA8, NUP153, TOP1, RANGAP167345110912.19732540861812751.00.43908240539871174 0.3904362300746767
REACTOME_PATHWAYR-HSA-70326~Glucose metabolism90.95137420718816070.1355370179540326NUP93, PKM, NUP133, NUP153, ALDOA, GAPDH, PRKACB, PGM2L1, HK167384110911.76570791763956710.00.44068602648093530.3918621851166309
REACTOME_PATHWAYR-HSA-165054~Rev-mediated nuclear export of HIV RNA50.52854122621564480.14798052872760584NUP93, XPO1, NUP133, NUP153, RANGAP167334110912.42352067126999371.00.46207253384674040.4108792698544876
REACTOME_PATHWAYR-HSA-4570464~SUMOylation of RNA binding proteins60.63424947145877380.15356616452689267NUP93, PHC2, HNRNPK, NUP133, NUP153, BMI167347110912.10382219974076051.00.47354121552509787 0.4210773013512744
REACTOME_PATHWAYR-HSA-3301854~Nuclear Pore Complex (NPC) Disassembly50.52854122621564480.15959870931935793NUP93, CCNB2, NUP133, NEK6, NUP15367335110912.35427722351942271.00.48317552726939667 0.4296442513111023
REACTOME_PATHWAYR-HSA-9615933~Postmitotic nuclear pore complex (NPC) reformation40.42283298097251587 0.22271053885035386NUP93, NUP133, RANGAP1, TNPO167327110912.44147267624236 41.00.57778048477195090.5137678748029869
REACTOME_PATHWAYR-HSA-4419969~Depolymerization of the Nuclear Lamina30.31712473572938690.22952517528490088LPIN1, LEMD2, LMNB167315110913.29598811292719151.00.58153089001276790.5171027706345501
REACTOME_PATHWAYR-HSA-3371453~Regulation of HSF1-mediated heat shock response70.73995771670190270.23896319374386943NUP93, GSK3B, NUP133, BAG2, NUP153, ATM, SIRT167369110911.67187802829640141.00.59531513344374540.5293598503386103
REACTOME_PATHWAYR-HSA-1169408~ISG15 antiviral mechanism70.73995771670190270.28045596664705863NUP93, EIF4A1, NUP133, STAT1, NUP153, RPS27A, JAK167373110911.58026827332125631.00.65591118785337740.5832424353618317
REACTOME_PATHWAYR-HSA-168333~NEP/NS2 Interacts with the Cellular Export Machinery40.42283298097251587 0.28921266352303 09NUP93, XPO1, NUP133, NUP15367333110912.12644394382399461.00.66919847421196090.5950576161342365
REACTOME_PATHWAYR-HSA-159236~Transport of Mature mRNA derived from an Intron-Containing Transcript70.73995771670190270.30178818238420 93NUP93, NUP133, NCBP2, ZC3H11A, SRSF1, THOC3, NUP15367375110911.53812778603268941.00.68725219457534130.6111111551301793
REACTOME_PATHWAYR-HSA-168274~Export of Viral Ribonucleoproteins from Nucleus40.42283298097251587 0.32305873621900 35NUP93, XPO1, NUP133, NUP15367333110911.99756855328920 71.00.71291203849663340.6339281311732904

REACTOME_PATHWAYR-HSA-180746~Nuclear import of Rev protein40.422832980972515870.3230587362190035NUP93, NPM1, NUP133, NUP15367333110911.99756855328920710.0.71291203849663340.6339281311732904
REACTOME_PATHWAYR-HSA-159227~Transport of the SLBP independent Mature mRNA40.422832980972515870.34000178855595653NUP93, NUP133, NCBP2, NUP15367334110911.93881653701599510.0.74490910185521190.6623802227114147
REACTOME_PATHWAYR-HSA-159230~Transport of the SLBP Dependant Mature mRNA40.422832980972515870.3569145772321122NUP93, NUP133, NCBP2, NUP15367335110911.88342177881553810.0.75800608130300280.6740261807778001
REACTOME_PATHWAYR-HSA-176033~Interactions of Vpr with host cellular proteins40.422832980972515870.373766964422642NUP93, NUP133, NUP153, SLC25A567336110911.8311045071817731.00.78040740884872120.6939456532232425
REACTOME_PATHWAYR-HSA-5619102~SLC transporter disorders80.84566596194503170.3832441322440663NUP93, SLC26A2, SLC16A1, NUP133, SLC9A6, NUP153, SLC27A4, HK167398110911.3453012705825274 1.00.79311397460940460.7052444517444272
REACTOME_PATHWAYR-HSA-72202~Transport of Mature Transcript to Cytoplasm70.73995771670190270.40020654648083864NUP93, NUP133, NCBP2, ZC3H11A, SRSF1, THOC3, NUP15367384110911.37332838038632991.00.80272910306862670.7137943149617736
REACTOME_PATHWAYR-HSA-191859~snRNP Assembly50.52854122621564480.4014904933475593NUP93, NUP133, NCBP2, NUP153, WDR7767353110911.55471137402226021.00.80344460158351070.7144305430122273
REACTOME_PATHWAYR-HSA-194441~Metabolism of non-coding RNA50.52854122621564480.4014904933475593NUP93, NUP133, NCBP2, NUP153, WDR7767353110911.55471137402226021.00.80344460158351070.7144305430122273
REACTOME_PATHWAYR-HSA-159231~Transport of Mature mRNA Derived from an Intronless Transcript40.422832980972515870.45620839249840345NUP93, NUP133, NCBP2, NUP15367341110911.60779907947667881.00.87631269212105840.7792255386950669
REACTOME_PATHWAYR-HSA-6784531~tRNA processing in the nucleus50.52854122621564480.45661301845661617NUP93, NUP133, NUP153, RPP14, TSEN5467357110911.44560882145929441.00.87631269212105840.7792255386950669
REACTOME_PATHWAYR-HSA-159234~Transport of Mature mRNAs Derived from Intronless Transcripts40.422832980972515870.4721766590294275 7NUP93, NUP133, NCBP2, NUP15367342110911.56951814901294841.00.89511330713768920.795943223484812
REACTOME_PATHWAYR-HSA-3108214~SUMOylation of DNA damage response and repair proteins60.63424947145877380.503247428821425NUP93, PHC2, NUP133, PARP1, NUP153, BMI167377110911.28415121282877610.0.92227777430455950.8200981247566798
REACTOME_PATHWAYR-HSA-168271~Transport of Ribonucleoproteins into the Host Nucleus30.31712473572938690.5682704576033979NUP93, NUP133, NUP15367331110911.59483295786799581.00.98777603112004860.8783397945504876
REACTOME_PATHWAYR-HSA-5619107~Defective TPR may confer susceptibility towards thyroid papillary carcinoma (TPC)30.31712473572938690.5682704576033979NUP93, NUP133, NUP15367331110911.59483295786799581.00.98777603112004860.8783397945504876
REACTOME_PATHWAYR-HSA-170822~Regulation of Glucokinase by Glucokinase Regulatory Protein30.31712473572938690.5682704576033979NUP93, NUP133, NUP15367331110911.59483295786799581.00.98777603112004860.8783397945504876
REACTOME_PATHWAYR-HSA-180910~Vpr-mediated nuclear import of PICs30.31712473572938690.6026949642056106NUP93, NUP133, NUP15367333110911.49817641496690521.01.00.8897228637413395
REACTOME_PATHWAYR-HSA-4085377~SUMOylation of SUMOylation proteins30.31712473572938690.6191132876848832NUP93, NUP133, NUP15367334110911.45411240276199631.01.00.8897228637413395
REACTOME_PATHWAYR-HSA-68875~Mitotic Prophase90.95137420718816070.6274424529065213RB1, NUP93, CCNB2, NUP133, NEK6, NUP153, LPIN1, LEMD2, LMNB1673141110911.05191109987038021.01.00.8897228637413395
REACTOME_PATHWAYR-HSA-4551638~SUMOylation of chromatin organization proteins50.52854122621564480.6312465195498405NUP93, PHC2, NUP133, NUP153, BMI167371110911.16055919469267 31.01.00.8897228637413395
REACTOME_PATHWAYR-HSA-9609646~HCMV Infection101.05708245243128960.6395380594197339NUP93, ITGB1, HNRNPK, CREB1, NUP133, CHMP2B, NUP153, DYNLL2, EGFR, EZH2673160110911.02999628528974731.01.00.8897228637413395
REACTOME_PATHWAYR-HSA-71387~Metabolism of carbohydrates171.79704016913319230.6881134642253376SLC26A2, NUP133, NUP153, GNS, PGM2L1, FUT4, HS2ST1, HK1, NUP93, GYS1, VCAN, PKM, CALM3, ALDOA, RPS27A, GAPDH, PRKACB673290110910.96606548137521141.01.00.8897228637413395
REACTOME_PATHWAYR-HSA-168276~NS1 Mediated Effects on Host Pathways30.31712473572938690.7067189299141672NUP93, NUP133, NUP15367340110911.2359955423476971.01.00.8897228637413395
REACTOME_PATHWAYR-HSA-9609690~HCMV Early Events80.84566596194503170.7175672202268895NUP93, ITGB1, CREB1, NUP133, NUP153, DYNLL2, EGFR, EZH2673135110910.97658907049694571.01.00.8897228637413395
REACTOME_PATHWAYR-HSA-168325~Viral Messenger RNA Synthesis30.31712473572938690.7438378494814336NUP93, NUP133, NUP15367343110911.149763295207161.01.00.8897228637413395
REACTOME_PATHWAYR-HSA-211000~Gene Silencing by RNA70.73995771670190270.8447686309714395NUP93, TSNAX, HSP90AA1, NUP133, NUP153, TNRC6A, TNRC6B673137110910.84204075877701971.01.00.8897228637413395
REACTOME_PATHWAYR-HSA-72306~tRNA processing50.52854122621564480.8955404680072178NUP93, NUP133, NUP153, RPP14, TSEN5467310711 0910.77009068059046541.01.00.8955404680072178
REACTOME_PATHWAYR-HSA-9610379~HCMV Late Events50.52854122621564480.9270409662680104NUP93, HNRNPK, NUP133, CHMP2B, NUP153673116110910.71034226571706721.01.00.9270409662680104
REACTOME_PATHWAYR-HSA-5578749~Transcriptional regulation by small RNAs40.422832980972515870.9576381750111251NUP93, NUP133, NUP153, TNRC6A673105110910.62780725960517941.01.00.9576381750111251

Annotation Cluster 72Enrichment Score: 0.48955481554810454
CategoryTermCount%PValueGenesList TotalPop HitsPop TotalFold EnrichmentBonferroniBenjaminiFDR
REACTOME_PATHWAYR-HSA-9013408~RHOG GTPase cycle80.84566596194503170.16003289452767694VAV3, ITGB1, PREX1, DOCK4, ERBIN, PIK3R1, PAK2, LBR67374110911.78161519617686031.00.48317552726939667 0.4296442513111023
REACTOME_PATHWAYR-HSA-9013404~RAC2 GTPase cycle90.95137420718816070.16320664940020851VAV3, ITGB1, PREX1, DOCK4, SWAP70, ERBIN, PIK3R1, PAK2, LBR67388110911.68544846683776851.00.48761973766964 50.43359608525615867
REACTOME_PATHWAYR-HSA-9013423~RAC3 GTPase cycle90.95137420718816070.20909072330002104ITGB1, PREX1, SWAP70, RAPGEF1, ERBIN, ABL2, PIK3R1, PAK2, LBR67394110911.57786664980557021.00.56290476515425310.5005402441446648
REACTOME_PATHWAYR-HSA-9013149~RAC1 GTPase cycle151.58562367864693440.2314287210825979ITGB1, VAV3, DOCK4, SWAP70, ERBIN, PIK3R1, WASL, SYDE2, PREX1, SPATA13, ABL2, ECT2, SOS1, PAK2, LBR673185110911.33621139713264551.00.58379326584591 30.5191144966350558
REACTOME_PATHWAYR-HSA-9013026~RHOB GTPase cycle70.73995771670190270.24916507135628 96DIAPH1, PREX1, ERBIN, PIK3R1, ECT2, MYO9A, RHOB67370110911.64799405646359581.00.61658921767547970.5482769673617508
REACTOME_PATHWAYR-HSA-9013106~RHOC GTPase cycle50.52854122621564480.6636421626274728 2DIAPH1, PREX1, ERBIN, PIK3R1, LBR673374110911.11350949761053771.01.00.8897228637413395
REACTOME_PATHWAYR-HSA-9013148~CDC42 GTPase cycle90.95137420718816070.7296813653815738VAV3, PREX1, DNMBP, SPATA13, WASL, PIK3R1, ECT2, PAK2, LBR673155110910.95689977472079761.01.00.8897228637413395
REACTOME_PATHWAYR-HSA-8980692~RHOA GTPase cycle80.84566596194503170.7948916941134845VAV3, DIAPH1, PREX1, ERBIN, PIK3R1, ECT2, MYO9A, LBR673147110910.89686751372168471.01.00.8897228637413395

Annotation Cluster 73Enrichment Score: 0.4774176542632896
CategoryTermCount%PValueGenesList TotalPop HitsPop TotalFold EnrichmentBonferroniBenjaminiFDR
REACTOME_PATHWAYR-HSA-75955~RNA Polymerase II Transcription Elongation70.73995771670190270.13744677796067029CCNK, CCNT2, CCNT1, NCBP2, SSRP1, AFF4, CDC7367358110911.98895834400778 81.00.44439415336910750.39515948663692707
REACTOME_PATHWAYR-HSA-112382~Formation of RNA Pol II elongation complex 70.73995771670190270.13744677796067029CCNK, CCNT2, CCNT1, NCBP2, SSRP1, AFF4, CDC7367358110911.98895834400778 81.00.44439415336910750.39515948663692707
REACTOME_PATHWAYR-HSA-6807505~RNA polymerase II transcribes snRNA genes80.84566596194503170.16003289452767694INTS11, CCNK, CCNT2, CCNT1, NCBP2, SP1, RPRD2, ELL267374110911.78161519617686031.00.48317552726939670.4296442513111023
REACTOME_PATHWAYR-HSA-167152~Formation of HIV elongation complex in the absence of HIV Tat50.52854122621564480.28961272686611117CCNK, CCNT2, CCNT1, NCBP2, SSRP167345110911.83110450718177291.00.66919847421196090.5950576161342365
REACTOME_PATHWAYR-HSA-6796648~TP53 Regulates Transcription of DNA Repair Genes60.63424947145877380.32198736197912114CCNK, CCNT2, CCNT1, ATM, SSRP1, TP53673362110911.59483295786799581.00.71291203849663340.6339281311732904
REACTOME_PATHWAYR-HSA-167287~HIV elongation arrest and recovery40.422832980972515870.3230587362190035CCNK, CCNT2, CCNT1, SSRP167333110911.99756855328920710.0.71291203849663340.6339281311732904
REACTOME_PATHWAYR-HSA-167290~Pausing and recovery of HIV elongation40.422832980972515870.3230587362190035CCNK, CCNT2, CCNT1, SSRP167333110911.99756855328920710.0.71291203849663340.6339281311732904
REACTOME_PATHWAYR-HSA-674695~RNA Polymerase II Pre-transcription Events70.73995771670190270.3671856974537545CCNK, CCNT2, CCNT1, NCBP2, SSRP1, AFF4, CDC7367381110911.42419239447124 10.0.76944717495448190.6841997095238642
REACTOME_PATHWAYR-HSA-167172~Transcription of the HIV genome60.63424947145877380.4197297348553394CCNK, CCNT2, CCNT1, NCBP2, SSRP1, RNMT673370110911.41256633411165341.00.83035574258481030.738360184260 3534
REACTOME_PATHWAYR-HSA-167169~HIV Transcription Elongation30.31712473572938690.7438378494814336CCNT1, NCBP2, SSRP167343110911.149763295207161.01.00.8897228637413395
REACTOME_PATHWAYR-HSA-167200~Formation of HIV-1 elongation complex containing HIV-1 Tat30.31712473572938690.7438378494814336CCNT1, NCBP2, SSRP167343110911.149763295207161.01.00.8897228637413395
REACTOME_PATHWAYR-HSA-167246~Tat-mediated elongation of the HIV-1 transcript30.31712473572938690.7438378494814336CCNT1, NCBP2, SSRP167343110911.149763295207161.01.00.8897228637413395

Annotation Cluster 74Enrichment Score: 0.4345400236425602
CategoryTermCount%PValueGenesList TotalPop HitsPop TotalFold EnrichmentBonferroniBenjaminiFDR
REACTOME_PATHWAYR-HSA-9748787~Azathioprine ADME50.52854122621564480.05399102796228333VAV3, ABCC4, IMPDH2, GMPS, NME267324110913.43332095096582451.00.24053072354405405 0.21388219560380106
REACTOME_PATHWAYR-HSA-73817~Purine ribonucleoside monophosphate biosynthesis30.31712473572938690.1403828811282177IMPDH2, GMPS, PFAS67311110914.49452924490071610.0.44886260700221640.3991328778940655
REACTOME_PATHWAYR-HSA-8956320~Nucleotide biosynthesis30.31712473572938690.2067081779442922IMPDH2, GMPS, PFAS67314110913.5314158352791341.00.56236306495676360.500058559202754
REACTOME_PATHWAYR-HSA-9748784~Drug ADME90.95137420718816070.3495617256443578 3VAV3, ABCC4, ABCC1, SLC16A1, ABCB1, IMPDH2, GMPS, NME2, CYP3A4673110110911.34835877347021471.00.75646619773053960.6726569017326955
KEGG_PATHWAYhsa00983:Drug metabolism - other enzymes50.52854122621564480.7416415295086978IMPDH2, DPYD, GMPS, NME2, CYP3A4544818840.0030864197530864 1.01.00.741641529508697 8
REACTOME_PATHWAYR-HSA-15869~Metabolism of nucleotides50.52854122621564480.8580895688040288IMPDH2, DPYD, GMPS, NME2, PFAS67399110910.83232023053716951.01.00.8897228637413395
KEGG_PATHWAYhsa01232:Nucleotide metabolism30.31712473572938690.9705464786725988IMPDH2, GMPS, NME25448588400.57352941176470581.01.00.9705464786725988
KEGG_PATHWAYhsa00230:Purine metabolism40.422832980972515870.9872326909441815IMPDH2, GMPS, NME2, PFAS5441288840 0.50781251.01.00.9872326909441815

Annotation Cluster 75Enrichment Score: 0.4216353165536723
CategoryTermCount%PValueGenesList TotalPop HitsPop TotalFold EnrichmentBonferroniBenjaminiFDR
REACTOME_PATHWAYR-HSA-72187~mRNA 3'-end processing60.63424947145877380.2858506955643024NUDT21, CPSF7, NCBP2, ZC3H11A, SRSF1, THOC367359110911.67592615911552111.00.665832332544268900.59206441110832
REACTOME_PATHWAYR-HSA-159236~Transport of Mature mRNA derived from an Intron-Containing Transcript70.73995771670190270.3017881823842093NUP93, NUP133, NCBP2, ZC3H11A, SRSF1, THOC3, NUP15367375110911.53812778603268941.00.68725219457534130.6111111551301793
REACTOME_PATHWAYR-HSA-77595~Processing of Intronless Pre-mRNAs30.31712473572938690.3440071139334081NUDT21, CPSF7, NCBP267320110912.4719910846953941.00.74989223703974370.6668112736746942
REACTOME_PATHWAYR-HSA-73856~RNA Polymerase II Transcription Termination60.63424947145877380.3953303597613594NUDT21, CPSF7, NCBP2, ZC3H11A, SRSF1, THOC367368110911.45411240276199631.00.79478829868496150.7067332765571411
REACTOME_PATHWAYR-HSA-72202~Transport of Mature Transcript to Cytoplasm70.73995771670190270.40020654648083864NUP93, NUP133, NCBP2, ZC3H11A, SRSF1, THOC3, NUP15367384110911.37332838038632991.00.80272910306862670.7137943149617736
KEGG_PATHWAYhsa03015:mRNA surveillance pathway80.84566596194503170.4479189191386202 5PPP1CB, PNN, NUDT21, CPSF7, NCBP2, PPP2R5E, RNMT, TARDBP5441038840 1.26213592233009 71.00.75203229055378870.5092130870207472
REACTOME_PATHWAYR-HSA-75067~Processing of Capped Intronless Pre-mRNA30.31712473572938690.5317409597373535NUDT21, CPSF7, NCBP267329110911.70482143772096121.00.95098770198641240.8456272641437169

Annotation Cluster 76Enrichment Score: 0.40939422620536414
CategoryTermCount%PValueGenesList TotalPop HitsPop TotalFold EnrichmentBonferroniBenjaminiFDR
REACTOME_PATHWAYR-HSA-199220~Vitamin B5 (pantothenate) metabolism40.422832980972515870.1173532243114246 3SLC5A6, PANK2, FASN, COASY67320110913.29598811292719151.00.39931500304309430.3550746795668 8304
REACTOME_PATHWAYR-HSA-196849~Metabolism of water-soluble vitamins and cofactors80.84566596194503170.6562067624283141SLC5A6, CYB5A, PANK2, ABCC1, FASN, NAMPT, NMNAT2, COASY673127110911.03810649226053281.01.00.8897228637413395
REACTOME_PATHWAYR-HSA-196854~Metabolism of vitamins and cofactors111.16279069767441870.7678592021725658SLC5A6, CYB5A, PANK2, HSP90AA1, ABCC1, FASN, NAMPT, CALM3, NMNAT2, LDLR, COASY673198110910.91555225359088651.01.00.8897228637413395

Annotation Cluster 77Enrichment Score: 0.3633893302112478
CategoryTermCount%PValueGenesList TotalPop HitsPop TotalFold EnrichmentBonferroniBenjaminiFDR
REACTOME_PATHWAYR-HSA-111885~Opioid Signalling101.05708245243128960.10446050981363916CREB1, GRK2, GNB2, GNB4, GNAI3, CALM3, ITPR3, GNB5, GNG12, PRKACB67392110911.79129788746034021.00.37780869338575560.3359510654976627
REACTOME_PATHWAYR-HSA-442720~CREB1 phosphorylation through the activation of Adenylate Cyclase30.31712473572938690.2067081779442922CREB1, CALM3, PRKACB67314110913.5314158352791341.00.56236306495676360.500058559202754
KEGG_PATHWAYhsa04927:Cortisol synthesis and secretion70.73995771670190270.2079827883126306SCARB1, MC2R, CREB1, SP1, ITPR3, LDLR, PRKACB5446588401.751.00.42258923230400740.2861419253218357
REACTOME_PATHWAYR-HSA-9664323~FCGR3A-mediated IL10 synthesis50.52854122621564480.22204621178824605CREB1, SRC, CALM3, ITPR3, PRKACB67340110912.05999257057949461.00.57778048477195090.5137678748029869
KEGG_PATHWAYhsa04924:Renin secretion70.73995771670190270.24850480764615930EDNRA, CREB1, GNAI3, CALM3, ITPR3, PRKACB, CTSB5446988401.64855072463768121.00.49455668899452960.3354809680322 3154
REACTOME_PATHWAYR-HSA-112040~G-protein mediated events60.63424947145877380.2505562947755815CREB1, GRK2, GNAI3, CALM3, ITPR3, PRKACB67356110911.76570791763956711.00.61765868968148330.5492279519902861
REACTOME_PATHWAYR-HSA-1489509~DAG and IP3 signaling50.52854122621564480.2621568841391382CREB1, GRK2, CALM3, ITPR3, PRKACB67343110911.91627215867859810.0.62924914156942720.5595342914936453
KEGG_PATHWAYhsa04971:Gastric acid secretion70.73995771670190270.3238417003527787 8CA2, GNAI3, CALM3, ITPR3, ATP1A1, PRKACB, CFTR5447688401.49671052631578941.00.59370978398834250.4020103866504138
REACTOME_PATHWAYR-HSA-438064~Post NMDA receptor activation events70.73995771670190270.3561959773632 81NRAS, CREB1, PRKAA2, SRC, KRAS, CALM3, PRKACB67380110911.44199479940564631.00.75800608130300280.6740261807778001
REACTOME_PATHWAYR-HSA-112043~PLC beta mediated events50.52854122621564480.3735236330904622CREB1, GRK2, CALM3, ITPR3, PRKACB67351110911.61568044751332931.00.78040740884872120.6939456532232425
REACTOME_PATHWAYR-HSA-111931~PKA-mediated phosphorylation of CREB30.31712473572938690.3885322674123 83CREB1, CALM3, PRKACB67322110912.24726462245035811.00.79311397460940460.7052444517444272
REACTOME_PATHWAYR-HSA-111933~Calmodulin induced events40.422832980972515870.3905308459716145CREB1, GRK2, CALM3, PRKACB67337110911.78161519617686031.00.79311397460940460.7052444517444272
REACTOME_PATHWAYR-HSA-111997~CaM pathway40.422832980972515870.3905308459716145CREB1, GRK2, CALM3, PRKACB67337110911.78161519617686031.00.79311397460940460.7052444517444272
KEGG_PATHWAYhsa04925:Aldosterone synthesis and secretion80.84566596194503170.39686678644232964SCARB1, MC2R, CREB1, CALM3, ITPR3, ATP1A1, LDLR, PRKACB5449888401.32653061224489791.00.69560716964342400.4710067355579297
REACTOME_PATHWAYR-HSA-111996~Ca-dependent events40.422832980972515870.4236906460938251CREB1, GRK2, CALM3, PRKACB67339110911.69025031432163651.00.83628233448815370.7436301658662694
REACTOME_PATHWAYR-HSA-442755~Activation of NMDA receptors and postsynaptic events70.73995771670190270.4978152248295470 4NRAS, CREB1, PRKAA2, SRC, KRAS, CALM3, PRKACB67393110911.24042563897337 33 01.00.91688210117373730.8153002411475644
KEGG_PATHWAYhsa05031:Amphetamine addiction50.52854122621564480.6197345103168779PPP1CB, CREB1, CALM3, SIRT1, PRKACB5446988401.17753623188405801.00.95045821534175020.6435704530213733
KEGG_PATHWAYhsa04918:Thyroid hormone synthesis40.422832980972515870.6848067671036021 7CREB1, ITPR3, ATP1A1, PRKACB, HSP90B15447588401.08333333333333511.00.6848067671036021 7
KEGG_PATHWAYhsa05030:Cocaine addiction30.31712473572938690.8124775669191369CREB1, GNAI3, PRKACB5444988400.99489795918367351.01.00.8124775669191369
REACTOME_PATHWAYR-HSA-5578775~Ion homeostasis30.31712473572938690.8614286419264064CALM3, ITPR3, ATP1A167356110910.88285395881978351.01.00.8897228637413395
KEGG_PATHWAYhsa04911:Insulin secretion40.422832980972515870.9055327537901 08CREB1, ITPR3, ATP1A1, PRKACB5448688400.75581395348837211.01.00.9055327537901 08
KEGG_PATHWAYhsa04970:Salivary secretion40.422832980972515870.9426748064152947CALM3, ITPR3, ATP1A1, PRKACB5449788400.67010309278350521.01.00.9426748064152947
REACTOME_PATHWAYR-HSA-5576891~Cardiac conduction50.52854122621564480.9626710737739192CACNG8, CALM3, ITPR3, ATP1A1, HIPK2673131211 0910.62424017290287711.01.00.962671073739192
REACTOME_PATHWAYR-HSA-397014~Muscle contraction80.84566596194503170.9689403847218128CACNG8, TPM3, CALM3, ITPR3, ATP1A1, PAK2, MYL9, HIPK2673205110910.64311963179067161.01.00.9689403847218128
KEGG_PATHWAYhsa04740:Olfactory transduction30.31712473572938690.9999999999950219GRK2, CALM3, PRKACB54445388400.10761589403973511 1.01.00.9999999999950219

Annotation Cluster 78Enrichment Score: 0.3617857618198635
CategoryTermCount%PValueGenesList TotalPop HitsPop TotalFold EnrichmentBonferroniBenjaminiFDR
REACTOME_PATHWAYR-HSA-9820841~M-decay: degradation of maternal mRNAs by maternally stored factors40.422832980972515870.17518552220053485EIF4A1, CNOT1, CNOT9, ZFP36L267324110912.74665676077265971.00.50768647152763690.4514396149013783
REACTOME_PATHWAYR-HSA-429947~Deadenylation of mRNA30.31712473572938690.4316672761515900 87EIF4A1, CNOT1, CNOT967324110912.05999257057949461.00.83998816627442880.7469254265602394
REACTOME_PATHWAYR-HSA-429914~Deadenylation-dependent mRNA decay40.422832980972515870.6559486918837261EIF4A1, CNOT1, CNOT9, DCP267355110911.19854113197352421.01.00.8897228637413395
REACTOME_PATHWAYR-HSA-9816359~Maternal to zygotic transition (MZT)70.73995771670190270.7200120146566726YAP1, EIF4A1, CNOT1, EP300, CNOT9, TP53, ZFP36L267311711 0910.98597935002095481.01.00.8897228637413395

Annotation Cluster 79Enrichment Score: 0.35619283614877395
CategoryTermCount%PValueGenesList TotalPop HitsPop TotalFold EnrichmentBonferroniBenjaminiFDR
REACTOME_PATHWAYR-HSA-73980~RNA Polymerase III Transcription Termination30.31712473572938690.4102937966504575NFIX, NFIA, POLR3G67323110912.1495574649525161.00.81261617096599190.7225859893009772
REACTOME_PATHWAYR-HSA-749476~RNA Polymerase III Abortive And Retractive Initiation40.422832980972515870.45620839249840345NFIX, GTF3C4, NFIA, POLR3G67341110911.60779907947667881.00.87631269212105840.7792255386950669
REACTOME_PATHWAYR-HSA-74158~RNA Polymerase III Transcription40.422832980972515870.45620839249840345NFIX, GTF3C4, NFIA, POLR3G67341110911.60779907947667881.00.87631269212105840.7792255386950669

Annotation Cluster 80Enrichment Score: 0.3552573256098876
CategoryTermCount%PValueGenesList TotalPop HitsPop TotalFold EnrichmentBonferroniBenjaminiFDR
REACTOME_PATHWAYR-HSA-6783783~Interleukin-10 signaling70.73995771670190270.06284693150154191IL6, CXCL8, STAT3, LIF, CCL3, CXCL2, JAK167347110912.45459233030887 1.00.26629274399064090.2367900279801 3715
KEGG_PATHWAYhsa04061:Viral protein interaction with cytokine and cytokine receptor90.95137420718816070.27117487295141511IL6, CCL8, CXCL12, CXCL8, CCL3, IL6ST, IL6R, CXCL2, CX3CL154410088401.46250000000000011.00.52746819799695990.35715714974088825
REACTOME_PATHWAYR-HSA-380108~Chemokine receptors bind chemokines50.52854122621564480.4835448614787472CXCL12, CXCL8, CCL3, CXCL2, CX3CL167359110911.39660513259626741.00.91085135319855310.8099376429769016
KEGG_PATHWAYhsa05323:Rheumatoid arthritis70.73995771670190270.5223076688431 42IL6, CXCL12, CXCL8, IFNG, CCL3, HLA-DRA, CXCL25449388401.21010638297872331.00.84117346646950650.5695720023743367
KEGG_PATHWAYhsa04060:Cytokine-cytokine receptor interaction161.69133192389006350.8239697498447854BMPR2, CXCL8, LIF, LIFR, CXCL2, ACVR2B, TGFBR1, CX3CL1, IL6, CCL8, CXCL12, IFNG, CCL3, FAS, IL6ST, IL6R54429888840 0.87248322147651011.01.00.8239697498447854
REACTOME_PATHWAYR-HSA-375276~Peptide ligand-binding receptors90.95137420718816070.9262557817024676NLN, EDNRA, MC2R, CXCL12, CXCL8, PSAP, CCL3, CXCL2, CX3CL1673201110910.73790778647623691.01.00.9262557817024676
REACTOME_PATHWAYR-HSA-373076~Class A/1 (Rhodopsin-like receptors)121.26849894291754750.99271858546991NLN, EDNRA, MC2R, CXCL12, P2RY11, CXCL8, ADORA2B, PSAP, CCL3, LPAR1, CXCL2, CX3CL1673334110910.59209367298093261.01.00.99271858546991

Annotation Cluster 81Enrichment Score: 0.3489502673500739
CategoryTermCount%PValueGenesList TotalPop HitsPop TotalFold EnrichmentBonferroniBenjaminiFDR
KEGG_PATHWAYhsa00010:Glycolysis / Gluconeogenesis70.73995771670190270.22795188551660026LDHB, LDHA, PKM, ALDOA, GAPDH, HK1, ALDH9A15446788401.69776119402985071.00.457337430690537670.30967048598481545
KEGG_PATHWAYhsa01200:Carbon metabolism80.84566596194503170.5750920878056335PKM, MDH2, GPT2, SDHD, ALDOA, GAPDH, ACAT1, HK154411688401.12068965517241371.00.90818998024751030.6149499552773111
KEGG_PATHWAYhsa01230:Biosynthesis of amino acids50.52854122621564480.6848067671036021 7PKM, GPT2, ASNS, ALDOA, GAPDH5447588401.08333333333333511.00.6848067671036021 7

Annotation Cluster 82Enrichment Score: 0.2984687085771632
CategoryTermCount%PValueGenesList TotalPop HitsPop TotalFold EnrichmentBonferroniBenjaminiFDR
REACTOME_PATHWAYR-HSA-2559586~DNA Damage/Telomere Stress Induced Senescence90.95137420718816070.10464406972023238RB1, CCNA2, CDK2, TERF2IP, HMGA2, ATM, TP53, TERF2, LMNB167379110911.87746158331295710.0.37780869338575560.3359510654976627
REACTOME_PATHWAYR-HSA-171319~Telomere Extension By Telomerase40.422832980972515870.16003621041698424CCNA2, CDK2, TERF2IP, TERF267323110912.86607661993668831.00.48317552726939670.4296442513111023
REACTOME_PATHWAYR-HSA-180786~Extension of Telomeres40.422832980972515870.6046274414745064CCNA2, CDK2, TERF2IP, TERF267351110911.29254435801066351.01.00.8897228637413395
REACTOME_PATHWAYR-HSA-1500620~Meiosis70.73995771670190270.7200120146566726SUN2, RAD51, CDK2, TERF2IP, ATM, TERF2, LMNB167311711 0910.98597935002095481.01.00.8897228637413395
REACTOME_PATHWAYR-HSA-1474165~Reproduction90.95137420718816070.7230589217406379SUN2, RAD51, CATSPERB, CDK2, TERF2IP, ATM, PRDM1, TERF2, LMNB167315411 0910.96311340962158211.01.00.8897228637413395
REACTOME_PATHWAYR-HSA-1221632~Meiotic synapsis40.422832980972515870.8595353135658527SUN2, TERF2IP, TERF2, LMNB167378110910.84512515716081831.01.00.8897228637413395
REACTOME_PATHWAYR-HSA-73886~Chromosome Maintenance60.63424947145877380.9321728014450272CCNA2, NPM1, CDK2, TERF2IP, CENPM, TERF2673140110910.70628316705582671.01.00.9321728014450272
REACTOME_PATHWAYR-HSA-157579~Telomere Maintenance40.422832980972515870.9695183044931455CCNA2, CDK2, TERF2IP, TERF2673112110910.58856930587985571.01.00.9695183044931455

Annotation Cluster 83Enrichment Score: 0.28735240850435567
CategoryTermCount%PValueGenesList TotalPop HitsPop TotalFold EnrichmentBonferroniBenjaminiFDR
REACTOME_PATHWAYR-HSA-3906995~Diseases associated with O-glycosylation of proteins60.63424947145877380.40754654460860984LFNG, NOTCH2, ADAMTS5, NOTCH1, THBS2, THBS167369110911.43303830996834411.00.81181397908818490.721872672691802
REACTOME_PATHWAYR-HSA-3781865~Diseases of glycosylation101.05708245243128960.5112665685496314LFNG, NOTCH2, NUS1, ADAMTS5, VCAN, SLC26A2, NOTCH1, ALG9, THBS2, THBS1673144110911.14444031698860821.00.93330931759489100.8299074774458898
REACTOME_PATHWAYR-HSA-5668914~Diseases of metabolism151.58562367864693440.6593532691371629NOTCH2, NUS1, SLC26A2, NOTCH1, ALG9, THBS2, GNS, THBS1, LFNG, GYS1, ADAMTS5, VCAN, MC2R, CYP1B1, RPS27A673250110910.98879643387815751.01.00.8897228637413395

Annotation Cluster 84Enrichment Score: 0.27695870409629714
CategoryTermCount%PValueGenesList TotalPop HitsPop TotalFold EnrichmentBonferroniBenjaminiFDR
KEGG_PATHWAYhsa00510:N-Glycan biosynthesis50.52854122621564480.4117104800209589GANAB, ALG9, RPN2, MGAT5, STT3B5445388401.53301886792452821.00.70992239527938320.48069980370014664
REACTOME_PATHWAYR-HSA-9694635~Translation of Structural Proteins50.52854122621564480.45661301845661617GSK3B, GANAB, RPN2, MGAT5, RPS27A67357110911.44560882145929441.00.87631269212105840.7792255386950669
REACTOME_PATHWAYR-HSA-9683701~Translation of Structural Proteins30.31712473572938690.5317409597373535GSK3B, GANAB, RPS27A67329110911.70482143772096121.00.95098770198641240.8456272641437169
REACTOME_PATHWAYR-HSA-9772573~Late SARS-CoV-2 Infection Events50.52854122621564480.6200139988136307GSK3B, GANAB, RPN2, MGAT5, RPS27A67370110911.17713861175971141.01.00.8897228637413395
REACTOME_PATHWAYR-HSA-9694548~Maturation of spike protein30.31712473572938690.6652190194152422GANAB, RPN2, MGAT567337110911.33621139713264531.01.00.8897228637413395

Annotation Cluster 85Enrichment Score: 0.22813963520026992
CategoryTermCount%PValueGenesList TotalPop HitsPop TotalFold EnrichmentBonferroniBenjaminiFDR
REACTOME_PATHWAYR-HSA-5696394~DNA Damage Recognition in GG-NER50.52854122621564480.19628931448067022CUL4A, INO80D, PARP1, RPS27A, CUL4B67338110912.16841323218894161.00.54866029354032500.4878739251850206 5
REACTOME_PATHWAYR-HSA-5696395~Formation of Incision Complex in GG-NER50.52854122621564480.2621568841391382CUL4A, INO80D, PARP1, RPS27A, RNF111, CUL4B67343110911.91627215867859810.0.62924914156942720.5595342914936453
REACTOME_PATHWAYR-HSA-5696400~Dual Incision in GG-NER40.422832980972515870.45620839249840345CUL4A, PARP1, RPS27A, CUL4B67341110911.60779907947667881.00.87631269212105840.7792255386950669
REACTOME_PATHWAYR-HSA-110314~Recognition of DNA damage by PCNA-containing replication complex30.31712473572938690.5502667289492079CUL4A, RPS27A, CUL4B67330110911.64799405646359581.00.97307371558058910.8652663564395199
REACTOME_PATHWAYR-HSA-5696399~Global Genome Nucleotide Excision Repair (GG-NER)60.63424947145877380.5816534377898473CUL4A, INO80D, PARP1, RPS27A, RNF111, CUL4B67384110911.17713861175971141.01.00.8897228637413395
REACTOME_PATHWAYR-HSA-73893~DNA Damage Bypass30.31712473572938690.7967497117470075CUL4A, RPS27A, CUL4B67348110911.02999628528974731.01.00.8897228637413395
REACTOME_PATHWAYR-HSA-5696398~Nucleotide Excision Repair60.63424947145877380.7978122790668158CUL4A, INO80D, PARP1, RPS27A, RNF111, CUL4B673110110910.90715269163133711.01.00.8897228637413395
REACTOME_PATHWAYR-HSA-6781823~Formation of TC-NER Pre-Incision Complex30.31712473572938690.8318735976908991CUL4A, RPS27A, CUL4B67352110910.95076580180592051.01.00.8897228637413395
REACTOME_PATHWAYR-HSA-6782210~Gap-filling DNA repair synthesis and ligation in TC-NER30.31712473572938690.9019487147154625CUL4A, RPS27A, CUL4B67363110910.78475907450647421.01.00.9019487147154625
REACTOME_PATHWAYR-HSA-6782135~Dual incision in TC-NER30.31712473572938690.9067420295014748CUL4A, RPS27A, CUL4B67364110910.77249721396731051.01.00.9067420295014748
REACTOME_PATHWAYR-HSA-6781827~Transcription-Coupled Nucleotide Excision Repair (TC-NER)30.31712473572938690.9520498762056815CUL4A, RPS27A, CUL4B67377110910.64207560641438 81.01.00.9520498762056815

Annotation Cluster 86Enrichment Score: 0.2266434736819357
CategoryTermCount%PValueGenesList TotalPop HitsPop TotalFold EnrichmentBonferroniBenjaminiFDR
REACTOME_PATHWAYR-HSA-9033241~Peroxisomal protein import50.52854122621564480.5356865166361865ECH1, UBE2D1, MPV17, RPS27A, PEX2667363110911.30793179084412371.00.95508717420834490.849272553638234
KEGG_PATHWAYhsa04146:Peroxisome60.63424947145877380.5834564829955426PRDX1, ECH1, MPV17, SOD2, ABCD1, PEX265448388401.17469879518072311.00.90926072649966830.615674974683161
REACTOME_PATHWAYR-HSA-9609507~Protein localization101.05708245243128960.6685754016952283CYB5A, SERP1, ECH1, UBE2D1, MPV17, TIMM10, RPS27A, BCS1L, ABCD1, PEX2667316411 0911.00487442467292421.01.00.8897228637413395

Annotation Cluster 87Enrichment Score: 0.19243894389081959
CategoryTermCount%PValueGenesList TotalPop HitsPop TotalFold EnrichmentBonferroniBenjaminiFDR
KEGG_PATHWAYhsa05332:Graft-versus-host disease40.422832980972515870.5129012496321531IL6, IFNG, HLA-DRA, FAS5444488401.47727272727272731.00.83055552605581560.562368882846571
KEGG_PATHWAYhsa05330:Allograft rejection30.31712473572938690.6871212623190268IFNG, HLA-DRA, FAS5443388401.28289473684210531.00.6871212623190268
KEGG_PATHWAYhsa04940:Type I diabetes mellitus30.31712473572938690.7509586490261492IFNG, HLA-DRA, FAS5444388401.13372093023255821.01.00.7509586490261492

Annotation Cluster 88Enrichment Score: 0.1870194531116601
CategoryTermCount%PValueGenesList TotalPop HitsPop TotalFold EnrichmentBonferroniBenjaminiFDR
REACTOME_PATHWAYR-HSA-3906995~Diseases associated with O-glycosylation of proteins60.63424947145877380.40754654460860984LFNG, NOTCH2, ADAMTS5, NOTCH1, THBS2, THBS167369110911.43303830996834411.00.81181397908818490.721872672691802
REACTOME_PATHWAYR-HSA-5083635~Defective B3GALTL causes PpS30.31712473572938690.6652190194152422ADAMTS5, THBS2, THBS167337110911.33621139713264531.01.00.8897228637413395
REACTOME_PATHWAYR-HSA-5173214~O-glycosylation of TSR domain-containing proteins30.31712473572938690.6795545138224132ADAMTS5, THBS2, THBS167338110911.30104793931336511.01.00.8897228637413395
REACTOME_PATHWAYR-HSA-5173105~O-linked glycosylation40.422832980972515870.9695183044931455ADAMTS5, GALNT11, THBS2, THBS1673112110910.58856930587985571.01.00.9695183044931455

Annotation Cluster 89Enrichment Score: 0.1482221744629180 2
CategoryTermCount%PValueGenesList TotalPop HitsPop TotalFold EnrichmentBonferroniBenjaminiFDR
REACTOME_PATHWAYR-HSA-446203~Asparagine N-linked glycosylation192.008456659619450.575924055681220 4NUS1, COPA, MCFD2, SEC24A, ALG9, UBXN1, RPN2, SEC16A, DERL1, MANEA, DYNLL2, COPZ1, GANAB, RNF139, LMAN2, MGAT5, GNPNAT1, TMED7, RPS27A673304110911.02999628528974731.01.00.8892775248544696
REACTOME_PATHWAYR-HSA-5694530~Cargo concentration in the ER30.31712473572938690.6026949642056106MCFD2, SEC24A, LMAN267333110911.49817641496690521.01.00.8897228637413395
REACTOME_PATHWAYR-HSA-204005~COPII-mediated vesicle transport40.422832980972515870.7889282232248581MCFD2, SEC24A, LMAN2, SEC16A67368110910.96940826850799761.01.00.8897228637413395
REACTOME_PATHWAYR-HSA-948021~Transport to the Golgi and subsequent modification101.05708245243128960.7966695255182389COPA, MCFD2, SEC24A, LMAN2, MGAT5, SEC16A, TMED7, DYNLL2, MANEA, COPZ1673185110910.89080759808843021.01.00.8897228637413395
REACTOME_PATHWAYR-HSA-199977~ER to Golgi Anterograde Transport80.84566596194503170.8319757128668597COPA, MCFD2, SEC24A, LMAN2, SEC16A, TMED7, DYNLL2, COPZ1673154110910.85610080855251721.01.00.8897228637413395

Annotation Cluster 90Enrichment Score: 0.14680452696419885
CategoryTermCount%PValueGenesList TotalPop HitsPop TotalFold EnrichmentBonferroniBenjaminiFDR
REACTOME_PATHWAYR-HSA-5693532~DNA Double-Strand Break Repair121.26849894291754750.45585243090879257CCNA2, RAD51, UBXN1, PARP1, RIF1, CHEK2, EYA4, CDK2, ATM, TOPBP1, RPS27A, TP53673170110911.16328992220959711.00.87631269212105840.7792255386950669
REACTOME_PATHWAYR-HSA-9675135~Diseases of DNA repair40.422832980972515870.6046274414745064MSH6, RAD51, ATM, TOPBP167351110911.29254435801066351.01.00.8897228637413395
REACTOME_PATHWAYR-HSA-9709570~Impaired BRCA2 binding to RAD5130.31712473572938690.635004118616014RAD51, ATM, TOPBP167335110911.41256633411165341.01.00.8897228637413395
REACTOME_PATHWAYR-HSA-5685938~HDR through Single Strand Annealing (SSA)30.31712473572938690.6652190194152422RAD51, ATM, TOPBP167337110911.33621139713264531.01.00.8897228637413395
REACTOME_PATHWAYR-HSA-5693616~Presynaptic phase of homologous DNA pairing and strand exchange30.31712473572938690.7067189299141672RAD51, ATM, TOPBP167340110911.2359955423476971.01.00.8897228637413395
REACTOME_PATHWAYR-HSA-9701190~Defective homologous recombination repair (HRR) due to BRCA2 loss of function30.31712473572938690.7195656241477922RAD51, ATM, TOPBP167341110911.20584930960750911.01.00.8897228637413395
REACTOME_PATHWAYR-HSA-9675136~Diseases of DNA Double-Strand Break Repair30.31712473572938690.7195656241477922RAD51, ATM, TOPBP167341110911.20584930960750911.01.00.8897228637413395
KEGG_PATHWAYhsa03440:Homologous recombination30.31712473572938690.7268889661760481RAD51, ATM, TOPBP15444188401.18902439024390261.01.00.7268889661760481
REACTOME_PATHWAYR-HSA-5693579~Homologous DNA Pairing and Strand Exchange30.31712473572938690.7438378494814336RAD51, ATM, TOPBP167343110911.149763295207161.01.00.8897228637413395
REACTOME_PATHWAYR-HSA-5685942~HDR through Homologous Recombination (HRR)40.422832980972515870.8050567656790457RAD51, ATM, TOPBP1, RPS27A67370110910.94171088940776911.01.00.8897228637413395
REACTOME_PATHWAYR-HSA-5693607~Processing of DNA double-strand break ends50.52854122621564480.8471028482417224CCNA2, CDK2, ATM, TOPBP1, RPS27A67397110910.84948147240391541.01.00.8897228637413395
REACTOME_PATHWAYR-HSA-5693538~Homology Directed Repair70.73995771670190270.8588836842543648CCNA2, RAD51, PARP1, CDK2, ATM, TOPBP1, RPS27A673140110910.82399702823179791.01.00.8897228637413395
REACTOME_PATHWAYR-HSA-5693567~HDR through Homologous Recombination (HRR) or Single Strand Annealing (SSA)60.63424947145877380.9150755093693792CCNA2, RAD51, CDK2, ATM, TOPBP1, RPS27A673134110910.73790778647623691.01.00.9150755093693792

Annotation Cluster 91Enrichment Score: 0.13017994129139707
CategoryTermCount%PValueGenesList TotalPop HitsPop TotalFold EnrichmentBonferroniBenjaminiFDR
REACTOME_PATHWAYR-HSA-1428517~Aerobic respiration and respiratory electron transport151.58562367864693440.4299162705842142PDHX, MDH2, SDHD, ACAT1, MKLN1, LDHB, LDHA, PKM, RMND5A, NDUFS6, UQCRFS1, UQCRC2, NDUFV3, RPS27A, PDK1673215110911.149763295207161.00.83998816627442880.7469254265602394
REACTOME_PATHWAYR-HSA-611105~Respiratory electron transport60.63424947145877380.8165996123246964NDUFS6, MDH2, UQCRFS1, SDHD, UQCRC2, NDUFV3673112110910.88285395881978351.01.00.8897228637413395
REACTOME_PATHWAYR-HSA-163200~Respiratory electron transport, ATP synthesis by chemiosmotic coupling, and heat production by uncoupling proteins.60.63424947145877380.9211558753623236NDUFS6, MDH2, UQCRFS1, SDHD, UQCRC2, NDUFV3673136110910.72705620138099811.01.00.9211558753623236
KEGG_PATHWAYhsa00190:Oxidative phosphorylation60.63424947145877380.9322965073466185NDUFS6, UQCRFS1, SDHD, UQCRC2, NDUFV3, NDUFC2-KCTD1454413388400.70652173913043481.01.00.9322965073466185

Annotation Cluster 92Enrichment Score: 0.10596596626172611
CategoryTermCount%PValueGenesList TotalPop HitsPop TotalFold EnrichmentBonferroniBenjaminiFDR
REACTOME_PATHWAYR-HSA-8856688~Golgi-to-ER retrograde transport90.95137420718816070.5606279214263503COPA, KIF1B, KIF20A, KIF22, TMED7, PAFAH1B3, DYNLL2, COPZ1, AGPAT3673133110911.11518394798288421.00.97940341515308980.8708947851996027
REACTOME_PATHWAYR-HSA-6811434~COPI-dependent Golgi-to-ER retrograde traffic60.63424947145877380.7242769003041136COPA, KIF1B, KIF20A, KIF22, TMED7, COPZ1673991109 10.99878427664460351.01.00.8897228637413395
REACTOME_PATHWAYR-HSA-6811442~Intra-Golgi and retrograde Golgi-to-ER traffic101.05708245243128960.8702397826470483TGOLN2, COPA, KIF1B, KIF20A, KIF22, TMED7, PAFAH1B3, DYNLL2, COPZ1, AGPAT3673202110910.81583864181366131.01.00.8897228637413395
REACTOME_PATHWAYR-HSA-983189~Kinesins30.31712473572938690.8803881647308966KIF1B, KIF20A, KIF2267359110910.83796307955776051.01.00.8897228637413395
REACTOME_PATHWAYR-HSA-6807878~COPI-mediated anterograde transport40.422832980972515870.9490250638229034COPA, TMED7, DYNLL2, COPZ1673101110910.65267091345092911.01.00.9490250638229034

Annotation Cluster 93Enrichment Score: 0.09589694136085183
CategoryTermCount%PValueGenesList TotalPop HitsPop TotalFold EnrichmentBonferroniBenjaminiFDR
REACTOME_PATHWAYR-HSA-2514859~Inactivation, recovery and regulation of the phototransduction cascade30.31712473572938690.6503710100433835NMT1, CALM3, GNB5673361109 11.37332838038632991.01.00.8897228637413395
REACTOME_PATHWAYR-HSA-2514856~The phototransduction cascade30.31712473572938690.6652190194152422NMT1, CALM3, GNB567337110911.33621139713264531.01.00.8897228637413395
REACTOME_PATHWAYR-HSA-2187338~Visual phototransduction40.422832980972515870.9556225789236152NMT1, CALM3, GNB5, LDLR673104110910.63384386787061381.01.00.9556225789236152
REACTOME_PATHWAYR-HSA-9709957~Sensory Perception90.95137420718816070.9999999995303335MYO1C, DNAJC5, EPB41L3, NMT1, TWF1, CALM3, ITPR3, GNB5, LDLR673609110910.24354591967442302 1.01.00.9999999995303335

Annotation Cluster 94Enrichment Score: 0.06059593252474318
CategoryTermCount%PValueGenesList TotalPop HitsPop TotalFold EnrichmentBonferroniBenjaminiFDR
REACTOME_PATHWAYR-HSA-9662360~Sensory processing of sound by inner hair cells of the cochlea40.422832980972515870.7971263613833957MYO1C, DNAJC5, EPB41L3, TWF167369110910.95535887331222941.01.00.8897228637413395
REACTOME_PATHWAYR-HSA-9662361~Sensory processing of sound by outer hair cells of the cochlea30.31712473572938690.8472982419171 53MYO1C, EPB41L3, TWF167354110910.91555225359088641.01.00.8897228637413395
REACTOME_PATHWAYR-HSA-9659379~Sensory processing of sound40.422832980972515870.8473288275754982MYO1C, DNAJC5, EPB41L3, TWF167376110910.86736529287557671.01.00.8897228637413395

REACTOME_PATHWAYR-HSA-9709957~Sensory Perception90.95137420718816070.9999999995303335MYO1C, DNAJC5, EPB41L3, NMT1, TWF1, CALM3, ITPR3, GNB5, LDLR673609110910.243545919674423021.01.00.9999999995303335

Annotation Cluster 95Enrichment Score: 0.0399827468407046
CategoryTermCount%PValueGenesList TotalPop HitsPop TotalFold EnrichmentBonferroniBenjaminiFDR
KEGG_PATHWAYhsa04514:Cell adhesion molecules80.84566596194503170.8563474256104451ITGB1, CDH5, VCAN, VCAM1, NLGN4X, CDH1, HLA-DRA, SIGLEC154415788400.82802547770700641.01.00.8563474256104451
REACTOME_PATHWAYR-HSA-216083~Integrin cell surface interactions40.42283298097251587 0.8957637930792332ITGB1, VCAM1, CDH1, THBS167385110910.7755266148063981 1.01.00.8957637930792332
REACTOME_PATHWAYR-HSA-198933~Immunoregulatory interactions between a Lymphoid and a non-Lymphoid cell40.42283298097251587 0.9890272527683762ITGB1, VCAM1, CDH1, SIGLEC1673133110910.49563731021461527 1.01.00.9890272527683762

Annotation Cluster 96Enrichment Score: 0.02540797860709424
CategoryTermCount%PValueGenesList TotalPop HitsPop TotalFold EnrichmentBonferroniBenjaminiFDR
REACTOME_PATHWAYR-HSA-5419276~Mitochondrial translation termination40.42283298097251587 0.9044491162195226MRPS27, MRPS14, MRPS34, MTRF1L67387110910.75769841676487161.01.00.9044491162195226
REACTOME_PATHWAYR-HSA-5368287~Mitochondrial translation40.42283298097251587 0.926716762953706MRPS27, MRPS14, MRPS34, MTRF1L67393110910.70881464794133151.01.00.926716762953706
REACTOME_PATHWAYR-HSA-5368286~Mitochondrial translation initiation30.31712473572938690.9716692611480479MRPS27, MRPS14, MRPS3467387110910.56827381257365371.01.00.9716692611480479
REACTOME_PATHWAYR-HSA-5389840~Mitochondrial translation elongation30.31712473572938690.9716692611480479MRPS27, MRPS14, MRPS3467387110910.56827381257365371.01.00.9716692611480479

Annotation Cluster 97Enrichment Score: 0.014243711851908319
CategoryTermCount%PValueGenesList TotalPop HitsPop TotalFold EnrichmentBonferroniBenjaminiFDR
REACTOME_PATHWAYR-HSA-211897~Cytochrome P450 - arranged by substrate type30.31712473572938690.9156783050105238RXRA, CYP1B1, CYP3A467366110910.74908820748345261.01.00.9156783050105238
REACTOME_PATHWAYR-HSA-211945~Phase I - Functionalization of compounds30.31712473572938690.9898493604301278RXRA, CYP1B1, CYP3A4673106110910.46641341220667804 1.01.00.9898493604301278
REACTOME_PATHWAYR-HSA-211859~Biological oxidations40.42283298097251587 0.9999006092100623SLC26A2, RXRA, CYP1B1, CYP3A4673222110910.29693586602947675 1.01.00.9999006092100623
