# Supplementary material for: Plasma microRNA Signature as Predictive Marker of Clinical Response to Therapy During Multiple Sclerosis
Source: Ann Clin Transl Neurol. 2025 Jun 11;12(8):1595–607. doi: 10.1002/acn3.70093 (PMC12343307; doi:10.1002/acn3.70093)
Supplement: Supplementary file 4 — File S3. Supplementary File 3. [file ACN3-12-1595-s002.pdf]

name	AverageShortestPathLength	BetweennessCentrality	ClosenessCentrality	ClusteringCoefficient	Eccentricity	EdgeCount	Indegree	NeighborhoodConnectivity	Outdegree	PartnerOfMultiEdgedNodePairs	shared name	Stress
IL6	1.595.959.596	255.043	0.626582278	0.166783544	4	93	44	3.917.204.301	49	0	IL6	379924
EGFR	1.496.350.365	1.919.105.734	0.668292683	0.167367929	3	93	19	3.939.784.946	74	0	EGFR	256222
GSK3B	1.580.357.143	1.869.458.762	0.632768362	0.198596491	3	76	23	4.292.105.263	53	0	GSK3B	239346
TP53	1	1.802.761.953	1	0.112580645	1	125	115	32.504	10	0	TP53	186484
PTEN	1.4	1.432.737.937	0.714285714	0.193946518	3	83	52	4.214.457.831	31	0	PTEN	203526
HIF1A	1.513.761.468	1.240.820.192	0.660606061	0.187917669	3	87	29	4.172.413.793	58	0	HIF1A	214062
SRC	147.826.087	1.176.265.094	0.676470588	0.165602656	2	89	77	3.931.460.674	12	0	SRC	128414
SMAD4	1.551.724.138	1.100.338.617	0.644444444	0.235791091	2	63	50	4.674.603.175	13	0	SMAD4	170530
KRAS	1.701.149.425	1.099.415.065	0.587837838	0.214735283	4	74	35	4.444.594.595	39	0	KRAS	196506
STAT3	1.368.421.053	1.086.528.795	0.730769231	0.16967354	2	97	85	3.962.886.598	12	0	STAT3	140417
PPARG	1.596.491.228	1.054.317.118	0.626373626	0.225703325	3	69	44	4.627.536.232	25	0	PPARG	186178
HSP90AA1	1.660.377.358	1.039.560.598	0.602272727	0.1907277	3	72	25	4.147.222.222	47	0	HSP90AA1	160783
SIRT1	1.621.621.622	1.023.427.334	0.616666667	0.213704206	3	67	50	4.440.298.507	17	0	SIRT1	147358
IFNG	1.721.153.846	0.969642717	0.581005587	0.228365385	3	65	26	4.604.615.385	39	0	IFNG	186839
IGF1	159.223.301	0.936650756	0.62804878	0.238461538	3	66	20	4.727.272.727	46	0	IGF1	149067
PPARGC1A	1.803.571.429	0.930877084	0.554455446	0.220629047	4	47	30	4.542.553.191	17	0	PPARGC1A	119019
CXCL8	1.794.326.241	0.930499581	0.557312253	0.231313131	4	55	12	4.634.545.455	43	0	CXCL8	129666
HMGCR	2.180.952.381	0.757154281	0.458515284	0.251811594	4	24	10	4.716.666.667	14	0	HMGCR	136570
SMAD2	153.125	0.662881675	0.653061224	0.249435028	3	60	44	4.813.333.333	16	0	SMAD2	124602
PTPN11	185.106.383	0.624412318	0.540229885	0.262121212	3	45	31	4.942.222.222	14	0	PTPN11	72373
NOTCH1	1.636.363.636	0.572138878	0.611111111	0.266666667	3	60	33	4.968.333.333	27	0	NOTCH1	85966
CDK2	1.829.931.973	0.560239821	0.546468401	0.267287234	4	48	8	4.966.666.667	40	0	CDK2	90202
RUNX2	1.717.948.718	0.54597389	0.582089552	0.259353741	3	49	35	4.959.183.673	14	0	RUNX2	80023
MEF2C	1.844.155.844	0.495183259	0.542253521	0.165322581	3	32	11	3.671.875	21	0	MEF2C	58666
CYP3A4	2.2	0.49259109	0.454545455	0.217948718	4	13	4	41	9	0	CYP3A4	52709
RUNX1	1.8	0.489231169	0.555555556	0.240646259	4	49	35	4.659.183.673	14	0	RUNX1	58719
PAX6	2.261.904.762	0.478049844	0.442105263	0.178461538	5	26	19	4.173.076.923	7	0	PAX6	51354
SMAD3	1.533.333.333	0.46359131	0.652173913	0.240559441	3	66	51	473.030.303	15	0	SMAD3	116669
MET	1.791.666.667	0.463577272	0.558139535	0.307254623	3	38	15	5.342.105.263	23	0	MET	83385
GATA3	1.829.059.829	0.449071793	0.546728972	0.265306122	4	49	14	4.991.836.735	35	0	GATA3	60990
IGF1R	1.75	0.445651539	0.571428571	0.26010929	4	61	24	4.929.508.197	37	0	IGF1R	99874
CXCL12	1.895.104.895	0.442318973	0.527675277	0.249275362	4	46	7	4.673.913.043	39	0	CXCL12	81990
MAPK14	1.820.512.821	0.437615573	0.549295775	0.301020408	3	49	26	5.410.204.082	23	0	MAPK14	60685
THBS1	1.692.307.692	0.431953102	0.590909091	0.310344828	2	30	26	5.453.333.333	4	0	THBS1	56784
CDH1	1.623.376.623	0.425581271	0.616	0.231055901	3	70	7	4.604.285.714	63	0	CDH1	72054
NFE2L2	184.057.971	0.42305741	0.543307087	0.305496829	4	44	24	5.545.454.545	20	0	NFE2L2	69875
CCND1	15.875	0.422434206	0.62992126	0.206487342	3	80	6	437.125	74	0	CCND1	69636
SLC2A4	1.96	0.339368715	0.510204082	0.313846154	3	26	22	5.430.769.231	4	0	SLC2A4	46860
PARP1	1.75	0.334674929	0.571428571	0.275420875	3	55	34	5.034.545.455	21	0	PARP1	73561
GATA2	1.925	0.31130352	0.519480519	0.248739496	4	35	10	4.685.714.286	25	0	GATA2	39085
SP3	1.833.333.333	0.309029857	0.545454545	0.357142857	2	7	5	5.385.714.286	2	0	SP3	48004
SP1	1.807.692.308	0.290275721	0.553191489	0.302564103	3	40	33	54.375	7	0	SP1	64782
CREB1	1.680.555.556	0.273007425	0.595041322	0.267318879	3	62	7	4.961.290.323	55	0	CREB1	48395
FOXO1	1.682.926.829	0.267955586	0.594202899	0.262021858	3	61	11	4.952.459.016	50	0	FOXO1	52107
FOXO3	1.702.479.339	0.247965148	0.587378641	0.249867795	3	62	15	4.846.774.194	47	0	FOXO3	66135
SCARB1	1.947.368.421	0.238101978	0.513513514	0.259139785	4	31	23	4.819.354.839	8	0	SCARB1	32196
PAX7	2.280.701.754	0.230676801	0.438461538	0.252380952	4	15	11	4.526.666.667	4	0	PAX7	13378
PINK1	2.051.724.138	0.224588193	0.487394958	0.292857143	4	21	13	510.952.381	8	0	PINK1	33171
FOXA1	1.950.819.672	0.222834091	0.512605042	0.275201613	4	32	7	51.375	25	0	FOXA1	32138
GRB2	1.894.736.842	0.21705527	0.527777778	0.320855615	4	34	6	5.479.411.765	28	0	GRB2	24488
MYH1	3.016.393.443	0.216661128	0.331521739	0.3	5	6	2	2.216.666.667	4	0	MYH1	20510
FBXW7	1.936.507.937	0.191993304	0.516393443	0.284090909	4	33	5	5.306.060.606	28	0	FBXW7	17633

TGFBR1		1.571.428.571	0.178814233	0.636363636	0.29374111	2	38	32		5.323.684.211	6	0	TGFBR1	18821
IRF1		2.154.929.577	0.175390332	0.464052288	0.327586207	4	29	18		5.393.103.448	11	0	IRF1	27181
LPAR1		2.262.295.082	0.175241588	0.442028986	0.3	4	10	4		51	6	0	LPAR1	15161
CARM1		2.213.793.103	0.17172039	0.451713396	0.219298246	4	19	1		4.373.684.211	18	0	CARM1	18768
CDH5		1.930.555.556	0.170123211	0.517985612	0.255793226	3	34	3		4.835.294.118	31	0	CDH5	32113
MYH2	2.6		0.167870472	0.384615385	0.261904762	5	7	3		2.857.142.857	4	0	MYH2	17256
STAT1	1.9		0.164371028	0.526315789	0.223188406	3	70	66	45.5		4	0	STAT1	21041
E2F1		1.833.333.333	0.163995455	0.545454545	0.305050505	3	45	9		5.406.666.667	36	0	E2F1	38409
LAMP1		2.197.183.099	0.158924825	0.455128205	0.236263736	4	14	6		4.771.428.571	8	0	LAMP1	18437
PAX3		2.586.206.897	0.150337374	0.386666667	0.242857143	5	15	10		4.646.666.667	5	0	PAX3	11267
PIK3CG		2.050.847.458	0.140809266	0.487603306	0.391304348	4	24	14		65.125	10	0	PIK3CG	18120
LRP5		2.116.666.667	0.140776873	0.472440945	0.274853801	3	19	9		4.647.368.421	10	0	LRP5	18190
RARA		1.785.714.286	0.139799493	0.56	0.289314516	3	32	18		5.309.375	14	0	RARA	32225
RXRA		1.909.090.909	0.13139378	0.523809524	0.213333333	3	25	16	41.16		9	0	RXRA	25694
GJA1		2.061.946.903	0.12682055	0.484978541	0.289473684	3	20	7		56	13	0	GJA1	23893
PRKACB		1.960.784.314	0.123908284	0.51	0.292436975	3	35	25		5.314.285.714	10	0	PRKACB	28865
CYP1B1		2.450.704.225	0.121827042	0.408045977	0.236111111	4	9	2		4.588.888.889	7	0	CYP1B1	22816
CFTR		2.154.929.577	0.121213768	0.464052288	0.201754386	4	19	3		4.794.736.842	16	0	CFTR	17150
CDC27		215.483.871	0.117974703	0.464071856	0.340659341	4	14	3		5.407.142.857	11	0	CDC27	20217
RET		190.625	0.110867265	0.524590164	0.315	3	25	18	55.6		7	0	RET	17371
NFIA		3.370.967.742	0.103532684	0.296650718	0.3	6	6	3	25.5		3	0	NFIA	11114
NOTCH2		1.901.960.784	0.095072144	0.525773196	0.394586895	3	27	15		6.174.074.074	12	0	NOTCH2	14918
MRC1		2.216.666.667	0.094905644	0.45112782	0.286549708	4	19	12		4.263.157.895	7	0	MRC1	15486
ETS1		18.359.375	0.092994154	0.544680851	0.299358974	4	40	6		52.925	34	0	ETS1	19879
NLRP3		2.084.745.763	0.090151577	0.479674797	0.328063241	4	23	14		5.173.913.043	9	0	NLRP3	18605
KHSRP		25.375	0.088619243	0.39408867	0.178571429	4	8	3	43.75		5	0	KHSRP	6389
STAT5A		1.833.333.333	0.085218569	0.545454545	0.334994463	2	43	41		5.658.139.535	2	0	STAT5A	17491
NFIX		2.573.770.492	0.078661195	0.388535032	0.236111111	5	9	3		4.755.555.556	6	0	NFIX	8794
LMO2		2.346.153.846	0.077591788	0.426229508	0.265151515	4	12	7		3.966.666.667	5	0	LMO2	7644
LDHA		2.033.333.333	0.072850239	0.491803279	0.277056277	4	22	12		5.540.909.091	10	0	LDHA	21318
LIF		2.651.162.791	0.064225972	0.377192982	0.346405229	6	18	13		5.666.666.667	5	0	LIF	10168
NR2F2	2.3		0.060075691	0.434782609	0.209090909	3	11	6		3.754.545.455	5	0	NR2F2	7035
HMGA2		2.294.117.647	0.05892416	0.435897436	0.324275362	5	24	8		5.545.833.333	16	0	HMGA2	11414
SFRP1	2.1		0.058192798	0.476190476	0.275	3	16	12		47	4	0	SFRP1	10721
HES1		2.336.448.598	0.05757604	0.428	0.319444444	4	9	1		56	8	0	HES1	4000
LAMP2		2.403.225.806	0.056518312	0.416107383	0.190909091	4	11	5		3.954.545.455	6	0	LAMP2	6467
THRB		1.909.090.909	0.055835364	0.523809524	0.333333333	2	7	6		5.714.285.714	1	0	THRB	2690
PLAU		2.034.482.759	0.044137603	0.491525424	0.363636364	4	23	14		5.965.217.391	9	0	PLAU	8874
SHC1		1.833.333.333	0.042577706	0.545454545	0.361742424	3	33	25		5.845.454.545	8	0	SHC1	9520
TAB2		21.875	0.041999965	0.457142857	0.318181818	3	12	8		5.083.333.333	4	0	TAB2	8548
HOXB4		253.164.557	0.04108036	0.395	0.267857143	4	8	2		38	6	0	HOXB4	4154
HIPK2		2.265.822.785	0.039178619	0.441340782	0.324175824	4	14	6		5.571.428.571	8	0	HIPK2	6813
PRDX3		2.552.631.579	0.032708924	0.391752577	0.227272727	4	11	9		3.590.909.091	2	0	PRDX3	6817
HSP90B1	2.12		0.031860209	0.471698113	0.367647059	4	17	6		6.782.352.941	11	0	HSP90B1	7466
MYL9	2.5		0.029941549	0.4	0.107142857	5	8	4	23.5		4	0	MYL9	3899
PHB		2.559.322.034	0.029364035	0.390728477	0.203296703	5	14	7		3.964.285.714	7	0	PHB	5862
YAP1		1	0.027504515	1	0.359879032	1	32	30		5.771.875	2	0	YAP1	3690
TERF2		1.833.333.333	0.026978326	0.545454545	0.3	2	5	3	53.2		2	0	TERF2	1422
THBS2		1	0.025888634	1	0.254545455	1	11	9		3.281.818.182	2	0	THBS2	4230
XIAP		1.666.666.667	0.025120513	0.6	0.313492063	2	36	35		5.497.222.222	1	0	XIAP	2659
IL6R		2.547.945.205	0.022206745	0.392473118	0.347953216	5	19	9		5.310.526.316	10	0	IL6R	5900
ECT2		2.534.883.721	0.021648647	0.394495413	0.233333333	4	6	3		4.083.333.333	3	0	ECT2	1300
TOP1		1.727.272.727	0.021545962	0.578947368	0.25	2	12	9		4.808.333.333	3	0	TOP1	2575

FAS	2.235.849.057	0.018915768	0.447257384	0.3125	4	17	4	5.635.294.118	13	0	FAS	4722
CXCL2	2.387.323.944	0.013590264	0.418879056	0.404761905	5	15	3	4.993.333.333	12	0	CXCL2	4222
PRKAA2	2.153.846.154	0.01317421	0.464285714	0.357142857	4	14	8	5.478.571.429	6	0	PRKAA2	4120
RHOB	2.166.666.667	0.013138715	0.461538462	0.214285714	3	8	6	52.5	2	0	RHOB	2174
DPYD	2.402.877.698	0.012535036	0.416167665	0.3	4	6	2	5.566.666.667	4	0	DPYD	612
FZD7	2.442.477.876	0.011397905	0.40942029	0.333333333	4	9	3	3.777.777.778	6	0	FZD7	3108
PPP2R5E	2.604.166.667	0.010849063	0.384	0.303571429	4	8	6	52.625	2	0	PPP2R5E	1856
SIGLEC1	2.714.285.714	0.010160877	0.368421053	0.357142857	4	8	6	46.5	2	0	SIGLEC1	3097
DKK2	2.234.782.609	0.010145278	0.447470817	0.281818182	4	11	2	4.072.727.273	9	0	DKK2	2318
WEE1	1	0.010137702	1	0.302380952	1	21	20	5.080.952.381	1	0	WEE1	1644
CDH11	2.337.931.034	0.00875192	0.427728614	0.333333333	4	9	2	4.177.777.778	7	0	CDH11	2353
TAB3	2	0.008340527	0.5	0.305555556	3	9	7	4.222.222.222	2	0	TAB3	2702
G6PC	2.203.539.823	0.008198712	0.453815261	0.368131868	4	14	4	4.914.285.714	10	0	G6PC	1304
CHUK	1.924.137.931	0.007809198	0.519713262	0.340463458	3	34	2	5.882.352.941	32	0	CHUK	1591
ATM	1.706.586.826	0.007749015	0.585964912	0.24956166	3	59	1	4.813.559.322	58	0	ATM	1606
PRDM1	2.390.243.902	0.006941005	0.418367347	0.395833333	5	16	11	604.375	5	0	PRDM1	1944
CCNG1	2.560.810.811	0.006423736	0.390501319	0.277777778	5	10	3	45.2	7	0	CCNG1	1006
TFPI	1.857.142.857	0.006177472	0.538461538	0.366666667	2	6	4	6.416.666.667	2	0	TFPI	1873
TSC1	1	0.005725538	1	0.31372549	1	18	17	5.622.222.222	1	0	TSC1	1420
PSAP	2.416.666.667	0.005676708	0.413793103	0.25	3	4	3	42.25	1	0	PSAP	745
MMP13	2.016.393.443	0.005522754	0.495934959	0.402631579	4	20	8	63	12	0	MMP13	2298
SMAD5	2.4	0.005274207	0.416666667	0.277777778	3	10	8	40.1	2	0	SMAD5	943
HMGN2	2.75	0.004999746	0.363636364	0	3	2	1	5	1	0	HMGN2	197
ROR1	2.3	0.00493856	0.434782609	0.25	3	5	4	45	1	0	ROR1	1350
MYH4	2.627.118.644	0.004854721	0.380645161	0.35	5	5	2	16.6	3	0	MYH4	672
SEMA3A	2.333.333.333	0.004427057	0.428571429	0.305555556	3	9	6	5.222.222.222	3	0	SEMA3A	1188
MAP2K4	2.392.405.063	0.003575658	0.417989418	0.363636364	4	11	5	5.754.545.455	6	0	MAP2K4	710
CCNA2	1.926.380.368	0.003291029	0.51910828	0.28219697	4	33	2	5.148.484.848	31	0	CCNA2	642
PLAG1	2.852.941.176	0.002815164	0.350515464	0.166666667	4	3	2	3.266.666.667	1	0	PLAG1	206
MAFB	2.394.366.197	0.002441712	0.417647059	0.321428571	4	8	3	48	5	0	MAFB	795
CYP27B1	2.517.730.496	0.002404172	0.397183099	0.355555556	4	10	1	62.4	9	0	CYP27B1	903
FZD5	249.122.807	0.002345482	0.401408451	0.404761905	4	7	2	3.085.714.286	5	0	FZD5	308
HOXA10	2.45	0.001916146	0.408163265	0.285714286	4	7	1	5.385.714.286	6	0	HOXA10	453
HNF1B	2.451.612.903	0.001812423	0.407894737	0.321428571	4	8	3	50.25	5	0	HNF1B	438
IFNAR1	2.361.904.762	0.001722076	0.423387097	0.41025641	4	13	3	4.730.769.231	10	0	IFNAR1	757
CCNT1	2.615.384.615	0.001478321	0.382352941	0.388888889	4	10	2	57.5	8	0	CCNT1	1393
STMN1	1.909.090.909	0.001417018	0.523809524	0.243589744	2	13	12	51	1	0	STMN1	269
ATG12	2.145.833.333	0.00136219	0.466019417	0.3125	4	16	1	535.625	15	0	ATG12	264
G3BP2	1.818.181.818	0.001275739	0.55	0.166666667	2	3	1	45	2	0	G3BP2	145
PLK2	1.727.272.727	0.001234709	0.578947368	0.303571429	2	8	5	36.75	3	0	PLK2	229
MARCKS	2.274.509.804	0.00117257	0.439655172	0.366666667	4	6	3	6.316.666.667	3	0	MARCKS	549
FANCG	2.824.074.074	0.001061706	0.354098361	0.214285714	5	7	1	47	6	0	FANCG	64
PTK2B	2.395.833.333	7.50E-04	0.417391304	0.406593407	4	14	9	6.721.428.571	5	0	PTK2B	269
LDLR	2.571.428.571	6.89E-04	0.388888889	0.25	3	4	3	44	1	0	LDLR	244
HAX1	2.598.130.841	5.27E-04	0.384892086	0.416666667	4	4	1	37.25	3	0	HAX1	83
MFF	2.491.525.424	4.74E-04	0.401360544	0.333333333	4	4	1	42	3	0	MFF	112
CX3CL1	2.395.833.333	2.97E-04	0.417391304	0.472727273	5	11	1	5.809.090.909	10	0	CX3CL1	199
EYA4	3.542.372.881	5.92E-05	0.282296651	0	6	2	1	37	1	0	EYA4	14
RRAS2	2.5	2.65E-05	0.4	0.333333333	3	3	2	53	1	0	RRAS2	8
ABCA1	248.630.137	0	0.402203857	0.352564103	5	13	0	4.746.153.846	13	0	ABCA1	0
ABCB1	2.012.048.193	0	0.497005988	0.303418803	4	27	0	5.633.333.333	27	0	ABCB1	0
ZEB1	0	0	0	0.37197724	0	38	38	5.931.578.947	0	0	ZEB1	0
ADORA2B	2.463.636.364	0	0.405904059	0.5	4	2	0	88	2	0	ADORA2B	0

APAF1		2.047.337.278	0	0.488439306		0.411255411		4	22	0	62.5		22	0	APAF1	0
APC		2.173.913.043	0	0.46		0.304761905		4	15	0	57.2		15	0	APC	0
ARTN		252.173.913	0	0.396551724		0.428571429		4	7	0		4.785.714.286	7	0	ARTN	0
VHL		0	0		0	0.454545455		0	11	11		69	0	0	VHL	0
BTG2		2.469.135.802	0	0.405		0.3		4	6	0		5.233.333.333	6	0	BTG2	0
CA2		3.055.944.056	0	0.327231121		0.5		5	2	0		54	2	0	CA2	0
CAPRIN1		2.951.807.229	0	0.33877551		0.05		5	5	0		38	5	0	CAPRIN1	0
WDR77		0	0		0	0.25		0	5	5		55	0	0	WDR77	0
CCL3		2.179.310.345	0	0.458860759		0.266304348		5	24	0		4.041.666.667	24	0	CCL3	0
VEGFC		0	0		0	0.37012987		0	22	22		6.027.272.727	0	0	VEGFC	0
YWHAZ		0	0		0	0.254926108		0	29	29		4.868.965.517	0	0	YWHAZ	0
ST14		0	0		0	0.5		0	2	2		54	0	0	ST14	0
TAL1		0	0		0	0.304761905		0	15	15		4.186.666.667	0	0	TAL1	0
RGS5		0	0		0		0	0	2	2		22	0	0	RGS5	0
SLC6A8		0	0		0		0	0	2	2	22.5		0	0	SLC6A8	0
WNK1		0	0		0		0	0	2	2	39.5		0	0	WNK1	0
TRIM27		0	0		0	0.155555556		0	10	10		38	0	0	TRIM27	0
NR5A2		2.596.491.228	0	0.385135135		0.5		5	3	1		66	2	0	NR5A2	0
VCAN		0	0		0	0.3		0	5	5	40.8		0	0	VCAN	0
CYB5A		2.972.027.972	0	0.336470588		0.333333333		5	3	0		1.533.333.333	3	0	CYB5A	0
PTBP2		0	0		0		0	0	2	2		7	0	0	PTBP2	0
EDNRA		2.345.454.545	0	0.426356589		0.25		4	4	0		71	4	0	EDNRA	0
SLC7A11		1.909.090.909	0	0.523809524		0.4		2	6	5		7.116.666.667	1	0	SLC7A11	0
SPRY2		2.416.666.667	0	0.413793103		0.430555556		3	9	8		6.977.777.778	1	0	SPRY2	0
EPB41L3		2.513.274.336	0	0.397887324		0.5		4	2	0	100.5		2	0	EPB41L3	0
FOXJ3		3.209.302.326	0	0.311594203			0	6	1	0		26	1	0	FOXJ3	0
FSTL1	2.55		0	0.392156863		0.5		5	2	0	65.5		2	0	FSTL1	0
KLF3		0	0		0		0	0	1	1		35	0	0	KLF3	0
TOX		0	0		0		0	0	1	1		49	0	0	TOX	0
SP4		0	0		0		0	0	2	2	13.5		0	0	SP4	0
GLS		2.418.181.818	0	0.413533835		0.416666667		4	4	0		60	4	0	GLS	0
HMGB2		3.102.803.738	0	0.322289157			0	5	3	0		1.233.333.333	3	0	HMGB2	0
SEMA6A		0	0		0		0	0	1	1		38	0	0	SEMA6A	0
MT2A		2.733.333.333	0	0.365853659		0.5		5	4	0	78.75		4	0	MT2A	0
MYT1	1.5		0	0.666666667			0	2	1	0		21	1	0	MYT1	0
NEK6	2.5		0	0.4			0	3	1	0		63	1	0	NEK6	0
POLR3G		0	0		0	0.5		0	2	2	7.5		0	0	POLR3G	0
POU4F2		1.909.090.909	0	0.523809524		0.5		2	2	1	75.5		1	0	POU4F2	0
PDS5B		2.583.333.333	0	0.387096774			0	3	1	0		12	1	0	PDS5B	0
PHLPP2		237.254.902	0	0.421487603			0	4	1	0		83	1	0	PHLPP2	0
TGIF1		0	0		0	0.433333333		0	6	6	52.5		0	0	TGIF1	0
ZBTB10		0	0		0		0	0	1	1		5	0	0	ZBTB10	0
