# Supplementary material for: Plasma microRNA Signature as Predictive Marker of Clinical Response to Therapy During Multiple Sclerosis
Source: Ann Clin Transl Neurol. 2025 Jun 11;12(8):1595–607. doi: 10.1002/acn3.70093 (PMC12343307; doi:10.1002/acn3.70093)
Supplement: Supplementary file 5 — Table S1. Demographic and clinical data of pwRRMS and healthy controls. [file ACN3-12-1595-s004.docx]

**Supplementary Table 1.** Demographic and clinical data of pwRRMS and healthy controls

|  | Healthy controls | pwRRMS | *p*-value |
| --- | --- | --- | --- |
| Number of subjects | 19 | 19 | - |
| Age (year) | 36.8 ± 2.9 | 34.6 ± 2.6 | 0.5928 |
| Gender (Women/Men) | 13/6 | 13/6 | - |
| BMI (kg/m^2^) | 23.9 ± 1.0 | 24.0 ± 0.9 | 0.9796 |
| EDSS (scorse) | - | 2.0 ± 0.84 | - |
