# Supplementary material for: Plasma microRNA Signature as Predictive Marker of Clinical Response to Therapy During Multiple Sclerosis
Source: Ann Clin Transl Neurol. 2025 Jun 11;12(8):1595–607. doi: 10.1002/acn3.70093 (PMC12343307; doi:10.1002/acn3.70093)
Supplement: Supplementary file 6 — Table S2. Absolute number (cells/mm3) and percentage (of the lymphocyte count, indicated in parentheses) of different immune cell subpopulations in peripheral blood of healthy controls and pwRRMS before (T0) and after (T1, 3 months; T2, 6 months, T3, 12 months; T4, 24 months) DMF treatment. Data are presented as mean ± SD. [file ACN3-12-1595-s001.docx]

**Supplementary Table 2:** Absolute number (cells/mm3) and percentage (of the lymphocyte count, indicated in parentheses) of different immune cell subpopulations in peripheral blood of healthy controls and pwRRMS before (T0) and after (T1, 3 months; T2, 6 months, T3, 12 months; T4, 24 months) DMF treatment. Data are presented as mean ± SD.

| RRMS subjects |  |
| --- | --- |

| Cell  subpopulations | Healthy  controls | T0 | T1 | T2 | T3 | T4 |
| --- | --- | --- | --- | --- | --- | --- |
| Leukocytes | 5792 ± 1664 | 6156 ± 2566 | 4873 ± 1341 | 5237 ± 1635 | 4904 ± 2003 | 4896 ± 1717 |
| Lymphocytes | 2069 ± 456 | 1849 ± 598 | 1415 ± 483 | 1542 ± 814 | 1290 ± 737 | 1305 ± 461 |
| CD3^+^ | 1578 ± 428  (76 ± 6) | 1342 ± 434  (73± 7) | 993 ± 373  (70± 8) | 1032 ± 502  (68± 8) | 911 ± 559  (66± 14) | 935 ± 335  (71± 8) |
| CD4^+^ | 951 ± 255  (46 ± 7) | 807 ± 308  (43 ± 8) | 566 ± 194  (40 ± 5) | 664 ± 334  (43 ± 7) | 588 ± 400  (42 ± 10) | 597 ± 210  (46 ± 7) |
| CD8^+^ | 513 ± 219  (24 ± 6) | 445 ± 183  (25 ± 7) | 356 ± 158  (25 ± 6) | 314 ± 152  (21 ± 5) | 281 ± 174  (21 ± 9) | 289 ± 133  (21 ± 6) |
| CD16^+^CD56^+^ | 216 ± 107  (11 ± 5) | 199 ± 114  (11 ± 6) | 194 ± 137  (14 ± 7) | 249 ± 254  (14 ± 6) | 158 ± 82  (14 ± 5) | 157 ± 93  (12 ± 5) |
| CD19^+^ | 222 ± 78  (11 ± 4) | 253 ± 158  (14 ± 5) | 195 ± 82  (14 ± 5) | 212 ± 103  (15 ± 7) | 190 ± 107  (17 ± 10) | 188 ± 128  (15 ± 7) |
| CD3^+^CD45RA^+^ | 758 ± 324  (35 ± 9) | 651 ± 219  (36 ± 7) | 534 ± 178  (38 ± 7) | 556 ± 201  (39 ±11) | 592 ± 350  (45 ± 13) | 639 ± 233  (50 ± 9) |
| CD3^+^CD45RO^+^ | 820 ± 190  (40 ± 8) | 691 ± 275  (37 ± 7) | 463 ± 238  (32 ± 9) | 475 ± 394  (28 ± 12) | 319 ± 310  (22 ± 10) | 296 ± 139  (22 ± 7) |
| CD4^+^CD45RA^+^ | 396 ± 198  (18 ± 7) | 328 ± 145  (18 ± 6) | 296 ± 115  (21 ± 6) | 345 ± 142  (24 ± 8) | 355 ± 219  (27 ± 8) | 373 ± 138  (30 ± 8) |
| CD4^+^CD45RO^+^ | 555 ± 133  (28 ± 7) | 479 ± 241  (25 ± 7) | 271 ± 142  (19 ± 6) | 402 ± 275  (19 ± 9) | 232 ± 239  (16 ± 8) | 224 ± 123  (16 ± 6) |
| CD8^+^CD45RA^+^ | 247 ± 140  (12 ± 5) | 233 ± 117  (13 ± 6) | 148 ± 96  (12 ± 6) | 158 ± 90  (12 ± 7) | 195 ± 135  (15 ± 8) | 216 ± 113  (16 ± 6) |
| CD8^+^CD45RO^+^ | 266 ± 116  (13 ± 4) | 212 ± 106  (12 ± 5) | 188 ± 118  (12 ± 6) | 156 ± 138  (9 ± 56) | 87 ± 90  (6 ± 5) | 72 ± 48  (5 ± 3) |
